# Transmission of HIV-1 drug resistance mutations within partner-pairs: A cross-sectional study of a primary HIV infection cohort

**DOI:** 10.1371/journal.pmed.1002537

**Published:** 2018-03-27

**Authors:** Joanne D. Stekler, Ross Milne, Rachel Payant, Ingrid Beck, Joshua Herbeck, Brandon Maust, Wenjie Deng, Kenneth Tapia, Sarah Holte, Janine Maenza, Claire E. Stevens, James I. Mullins, Ann C. Collier, Lisa M. Frenkel

**Affiliations:** 1 University of Washington, Seattle, Washington, United States of America; 2 Seattle Children’s Research Institute, Seattle, Washington, United States of America; 3 Fred Hutchinson Cancer Research Center, Seattle, Washington, United States of America; University of Melbourne, AUSTRALIA

## Abstract

**Background:**

Transmission of human immunodeficiency virus type 1 (HIV-1) drug resistance mutations, particularly that of minority drug-resistant variants, remains poorly understood. Population-based studies suggest that drug-resistant HIV-1 is less transmissible than drug-susceptible viruses. We compared HIV-1 drug-resistant genotypes among partner-pairs in order to assess the likelihood of transmission of drug resistance mutations and investigate the role of minority variants in HIV transmission.

**Methods and findings:**

From 1992–2010, 340 persons with primary HIV-1 infection and their partners were enrolled into observational research studies at the University of Washington Primary Infection Clinic (UWPIC). Out of 50 partner-pairs enrolled, 36 (72%) transmission relationships were confirmed by phylogenetic distance analysis of HIV-1 envelope (*env*) sequences, and 31 partner-pairs enrolled after 1995 met criteria for this study. Drug resistance mutations in the region of the HIV-1 polymerase gene (*pol*) that encodes protease and reverse transcriptase were assessed by 454-pyrosequencing. In 25 partner-pairs where the transmission direction could be determined, 12 (48%) transmitters had 1–4 drug resistance mutations (23 total) detected in their HIV-1 populations at a median frequency of 6.0% (IQR 1.5%–98.7%, range 1.0%–99.6%). Of 10 major mutations detected in five transmitters at a frequency >95%, 100% (95% CI 69.2%–100%) were detected in recipients. All of these transmitters were antiretroviral (ARV)-naïve at the time of specimen collection. Fourteen mutations (eight major mutations and six accessory mutations) were detected in nine transmitters at low frequencies (1.0%–11.8%); four of these transmitters had previously received ARV therapy. Two (14% [95% CI 1.8%–42.8%]) G73S accessory mutations were detected in both transmitter and recipient. This number is not significantly different from the number expected based on the observed frequencies of drug-resistant viruses in transmitting partners. Limitations of this study include the small sample size and uncertainties in determining the timing of virus transmission and mutation history.

**Conclusions:**

Drug-resistant majority variants appeared to be commonly transmitted by ARV-naïve participants in our analysis and may contribute significantly to transmitted drug resistance on a population level. When present at low frequency, no major mutation was observed to be shared between partner-pairs; identification of accessory mutations shared within a pair could be due to transmission, laboratory artifact, or apolipoprotein B mRNA-editing enzyme, catalytic polypeptides (APOBECs), and warrants further study.

## Abstract 2

In a cross-sectional study, Joanne Stekler and colleagues investigate transmission of HIV drug-resistance mutations between serodiscordant partners.

## Author summary

**Why was this study done?:**

- Previous studies have shown that virus factors like replication capacity (the ability of a virus to multiply) can impact the likelihood of HIV-1 transmission.
- Little is known about whether HIV-1 drug-resistance mutations impact the likelihood of HIV transmission.

**What did the researchers do and find?:**

- We identified 31 participants with primary HIV-1 infection and their partners.
- We found that five transmitting partners, none of whom had taken antiretroviral (ARV) therapy at the time of specimen collection, had 10 drug resistance mutations that made up more than 95% of the transmitter’s viral population; all of these mutations were found in their recipient partners.
- We identified another 14 mutations at low frequencies (1.0%–11.8%) in nine transmitting partners; two of these low frequency mutations were also detected in two recipient partners.

**What do these findings mean?:**

- Our findings suggest that HIV-1 drug resistance may be spread to and by people who have never taken ARV medications.
- Although we identified low-frequency mutations that were in both the transmitter and recipient in two partner-pairs, the mutation that we detected is considered an “accessory” mutation that does not have a significant impact on the effectiveness of HIV treatment. We are uncertain if these this mutation was transmitted within the two partner-pairs where it was identified or was a result of cellular restriction factors like APOBECs that acted independently in both members of the partner-pairs.
- Because of its small size, this study was not able to definitively address whether HIV-1 drug resistance mutations reduce the likelihood of HIV-1 transmission.

## Introduction

Transmission of human immunodeficiency virus type 1 (HIV-1) drug resistance mutations remains poorly understood. At the level of the partner-pair, it is unclear whether drug resistance mutations reduce "transmission fitness," defined as the relative ability of a viral variant to infect a susceptible host, similar to but independent of the impact of resistance on viral replication capacity [1,2]. In support of the theory that drug resistance mutations lead to reduced transmission fitness, historical population level analyses suggested that, when compared to hypothetical cohorts of potential transmitters, the prevalence of transmitted HIV-1 drug resistance should be higher than observed [3–6]. In contrast, empiric studies of transmission pairs report high concordance of HIV-1 drug resistance mutations in transmitting and recipient partners when tested by consensus sequencing [7–10].

Minority drug-resistant variants, defined as variants at a frequency below the limit of detection by consensus sequencing, can be found in antiretroviral (ARV)-naïve persons with both recent [11,12] and established HIV-1 infection [13,14]. Controversy remains as to whether these minority variants detected in recently infected persons were transmitted [15], especially given that almost all heterosexual transmission and most male–male sexual transmission leads to establishment of infection with a single founder variant [16–18]. Minority variants could also result from viral polymerase errors or cellular restriction factors (e.g., apolipoprotein B mRNA-editing enzyme, catalytic polypeptides [APOBECs] [19]) or artifacts of laboratory procedures (i.e., reverse transcription PCR and sequencing methodologies [20]). If minority drug resistant variants are indeed transmitted, it is unclear if they are transmitted with equal likelihood as drug-susceptible virus (and are therefore solely dependent on the frequency of the variant in the transmitting partner) or if drug resistance mutations impact transmission fitness independently of their impact on replication capacity. The objective of this analysis was to investigate the probability of transmission of HIV-1 drug resistance mutations and assess the role of minority variants in HIV transmission.

## Methods

### Population

Individuals with primary HIV-1 infection have been enrolled into an observational cohort at the University of Washington Primary Infection Clinic (UWPIC) since 1992 [21–25] and were followed according to protocol (S1 Text). At the time of cohort entry, all participants were either HIV-1 antibody-negative with detectable HIV-1 RNA (acute infection) or HIV-1 antibody-positive with a negative or indeterminate western blot, negative "detuned" antibody test, or negative HIV test within one year of screening (early infection). All participants were enrolled within 240 days of infection, estimated to be the date of onset of seroconversion symptoms [21], or, for asymptomatic participants, the midpoint between the last negative and first positive HIV-1 tests. As part of the PIC Partners Study protocol, we attempted to identify persons who were sex or needle-sharing partners in the three months prior to date of HIV-1 infection of the PIC enrollee. We estimated the date of infection for transmitting partners similarly, using the date of onset of seroconversion symptoms for symptomatic participants if HIV-1 test results were consistent with those symptoms or a midpoint for asymptomatic participants if the last negative test occurred in the prior two years. If neither of these conditions were met, we considered the date of infection to be unknown but earlier than the first HIV-1–positive test or the date of infection in the recipient partner, whichever was earlier. The UW Institutional Review Board approved these studies, and participants gave written informed consent for participation.

### Partner confirmation methods

Partial *env* gp120 sequences from the C2V5 region (HXB2 positions 7,021–7,646) were analyzed to assess genetic similarity of viruses from putative partner-pairs, as published previously [26]; sequences obtained from partners-pairs were aligned and assessed for monophyly using a phylogenetic tree and genetic distance. Directionality of transmission within confirmed partnerships was determined by epidemiological data (e.g., timing and duration of the partnership), HIV-1 test timing and results of HIV-1 serological and virological assays, and genetic diversity of C2V5; reports of external partnerships were not considered to assign directionality due to concerns about reliability. When neither the laboratory nor epidemiological evidence was definitive, the directionality was considered unable to be determined. These partner-pairs were not excluded from analysis in order to provide as complete a description as possible of the partner-pairs we identified over time, the prevalence of minority drug-resistant variants in the population, and the likelihood of transmission between partner-pairs.

### 454-pyrosequencing methods

A subset of confirmed partner-pairs was selected for study if the recipient partner acquired HIV-1 after 1995 and plasma and/or peripheral blood mononuclear cells (PBMCs) were available for study. For recipient partners, we analyzed the first available specimens closest to the estimated date of infection and no later than one week following initiation of ARV therapy. For transmitters, we selected specimens that were closest to the recipient’s estimated date of infection, preferentially studying specimens prior to the transmission event when available. To minimize the risk of specimen mix-up and contamination, laboratory work on specimens from known partner-pairs was temporally spaced.

RNA was isolated from 1 mL of blood plasma using silica (NucliSENS miniMAG; bioMérieux Clinical Diagnostics, Marcy l'Etoile, France) and reverse transcribed (BluePrint First Strand cDNA Synthesis Kit; Takara Laboratories Inc, Mountain View, CA) with the RTA primer [27]. DNA was isolated from 7–30 million PBMCs (ArchivePure DNA Purification Manual; 5 Prime Inc, Gaithersburg, MD). Extracted DNA was quantified by using a NanoDrop 1000 Spectrophotometer (Thermo Fisher Scientific, Wilmington, DE). An amount of 150,000 PBMCs was assumed to contain 1 μg of DNA. HIV-1 in the cDNA and in 2 μL (median = 1.81 μg, IQR 1.34–2.41 μg) of DNA were quantified by real-time PCR of the *gag* region [28] to estimate the amplifiable HIV-1 templates. A total of 1,000 amplifiable HIV-1 templates from cDNA or DNA (using a maximum of 7 μg DNA split in one or more first round reactions of approximately 1 μg of DNA) were submitted to nested PCR (FastStart Taq DNA Polymerase; Roche Applied Science, Penzberg, Germany) of HIV-1 *pol* (HXB2 2,095–3,328) using first round primers NEF10 (HXB2 2,071–2,095, 5′-GARAGACAGGCTAATTTTTTAGGGA-3′) and RTA. Three smaller (approximately 340-bp) regions were amplified in second round PCR using primers—A1: Forward (HXB2 2,250–2,272, 5′-TTCCCTCARATCACTCTTTGGCA-3′)/Reverse (HXB2 2,556–2,581, 5′-TTTACTGGTACAGTTTCAATAGGACT-3′), A2: Forward (HXB2 2,610–2,632, 5′-GTTAAACAATGGCCATTGACAGA-3′)/Reverse (HXB2 2,931–2,952, 5′-TACTAGGTATGGTRAATGCAGT-3′), A3: Forward (HXB2 2,923–2,947, 5′-GRAAGTATACTGCATTYACCATACC-3′)/Reverse (HXB2 3,252–3,275, 5′-CTGTACTGTCCATTTATCAGGATG-3′). Each primer pair was modified with the 454 adaptors A and B and one of 14 prespecified Multiplex Identifiers (MIDs) (454 Life Sciences; Branford, CT).

Second round amplicons were purified (High Pure PCR Product Purification Kit; Roche Applied Science, Mannheim, Germany) and quantified (Quant-iT PicoGreen dsDNA Assay Kit; Life Technologies, Grand Island, NY) separately. Amplicons diluted to 1 × 10^7^ molecules/μL were submitted to emulsion PCR as pools of barcoded amplicons from 14 participants on a 2-region gasket using the GS FLX Titanium System per manufacturer’s instructions (454 Life Sciences; Branford, CT).

Sequence read quality filtering and alignment generation for each sample were performed as previously described [29]. Forward and reverse reads were required to be in agreement to determine the frequency of “major” and “accessory” mutations at codons conferring resistance to protease inhibitors (PIs), nucleoside reverse transcriptase inhibitors (NRTIs), and non-nucleoside reverse transcriptase inhibitors (NNRTIs), including mutations for surveillance of transmitted drug resistance [30] as well as G-to-A mutations consistent with APOBEC effects, as defined in the Stanford University HIV Drug Resistance Database [31].

Errors introduced by PCR and 454-pyrosequencing were estimated at each nucleotide and averaged across the length of the amplicon by including an HIV-1 subtype B plasmid in each 454-pyrosequencing plate. Errors in the plasmid used as control varied across sites but were <0.15% at all nucleotide positions. Given that our estimated median input was 1,000 templates, we conservatively considered a mutation to be present if found in ≥1.0% of pyrosequencing reads in order to minimize the chance of false positive classifications for our prespecified analysis.

### Longitudinal analysis to evaluate potential transmission of major HIV-1 drug resistance mutations

After one mixture (M184M/V) was identified in a recipient partner (PIC 90629), specimens from two later time points were evaluated to assess the intrahost dynamics of the M184V mutation over time and determine whether this mixture arose from a single transmitted variant and evolution in the recipient or from initial infection by two transmitted variants. RNA was silica-extracted from plasma and reverse transcribed from primers BH2 (HXB2 7,697–7,725, 5′-CCTTGGTGGGTGCTACTCCTAATGGTTCA-3′) and NER10 (HXB2 3,303–3,328, 5′-AAYTTCTGTATATCATTGACAGTCCA-3′) using conditions identical to those for pyrosequencing. Nested single genome amplification (SGA) of C2V5 in *env* was performed for cDNA from PIC 90629 and PBMC DNA from PIC 52647. Additionally, an approximately 1-Kb region of *pol* encoding RT (HXB2 2,278–3,243) was amplified from 90629’s cDNA (MyTaq DNA polymerase, Bioline USA Inc.; Taunton, MA). Amplification of *env* was performed as described previously [23]; first-round *pol* primers: NEF10 and NER10, second-round *pol* primers: NEF11 (HXB2 2,256–2,278, 5′-CAAATCACTCTTTGGCARCGACC-3′) and NER11 (HXB2 3,243–3,265, 5′-CAYTTGTCAGGATGGAGTTCATA-3′). Positive reactions visualized in a 1% agarose gel were purified (ExoSAP-IT, Affymetrix, Inc.; Santa Clara, CA) and directly sequenced (BigDye Terminator v3.1, Life Technologies; Carlsbad, CA). *env* sequences were aligned to sequences used for partner-pair confirmation, and a maximum-likelihood tree was generated as described previously [23] except for the use of the Hasegawa, Kishino, and Yano (HKY85) model of evolution. A midpoint-rooted tree was assessed for monophyly.

### Statistical analysis

Our primary, prespecified objectives were to describe transmission of drug resistance mutations between partner-pairs and evaluate whether the likelihood of transmission of minority variants was different than would be expected by chance, conditional on the frequency of the HIV-1 drug resistance mutation in the viral population of the transmitter (see S1 Text). If a mutation was identified in both plasma and PBMCs, we used the mean of the two frequencies. The observed probability and exact binomial 95% CI were first calculated from apparent transmissions in the data using the predetermined 1% cutoff. The exact binomial distribution was then used to calculate the probability that two or more minority variants would have been transmitted by chance, based on the observed frequencies of minority variants identified in transmitters and assuming that mutations would be transmitted independently. Specifically, for each of the *i* mutations, conditional on each observed percentage mutant *p*_*i*_, we calculated the binomial probabilities B(1,*p*_*i*_). The probability of 2 or more mutants being transmitted by chance in the populations was obtained by assuming all transmissions were independent and calculating 1 – [P (T = 0) + P (T = 1)] where P (T = x) = Σ Π_ι_ B(1, p_i_) for all possible combinations resulting in x (x = 0,1) transmissions. When including the partner-pairs where the transmitting partner could not be determined conclusively, we calculated the range of possible probabilities that 2 or more transmissions occurred. A post-hoc analysis excluded data from these partner-pairs.

Given that many of the mutations detected at frequencies between 1% and 2% were consistent with APOBEC effects and were frequently detected in the PBMCs of recipients but not in transmitters, a second post-hoc analysis considered only mutations that were present in ≥2% of the viral population. All analyses were performed using Stata v14SE (StataCorp LP, College Station, TX), SAS v9.3 (SAS Institute, Cary, NC), and R (R Core Team, R: A Language and Environment for Statistical Computing, Vienna, Austria) software.

## Results

From 1992 to 2010, 340 persons were enrolled in the cohort, 50 partner-pairs were identified, and 36 (72%) HIV-1 transmissions were confirmed between putative partner-pairs. Thirty-one (86%) of these pairs met criteria for study; the remaining partner-pairs were enrolled prior to 1995 before modern, suppressive ARV therapy became available or had insufficient specimens for analysis. Demographic and other characteristics of these 62 participants are shown in **Table 1**. All transmissions occurred sexually (although one partner-pair reported injection drug use [IDU], they denied sharing needles or paraphernalia). In 25 partner-pairs where the directionality of transmission could be determined, plasma and PBMC specimens for pyrosequencing were obtained from 25 male recipient partners at a median of 22 (IQR 13–33) and 24 (IQR 19–41) days after infection, respectively. Plasma and PBMC specimens from the 24 male and one female transmitters were obtained at a median of 22 (IQR −1–50) and 23 (IQR 2–57) days after the estimated date of HIV-1 infection of the recipient, respectively. In the remaining 6 partner-pairs, similar levels of viral diversity within the partnership suggested that both members of the partnership acquired HIV-1 infection around the same time, and epidemiologic data regarding the timing of infections and other partnerships were inconclusive, precluding determination of the direction of transmission. Plasma and PBMC specimens were obtained from these 12 participants a median of 98 (IQR 77–124) and 164 (IQR 97–173) days, respectively, after the earliest possible date of infection of either partner in the pair.

**Table 1:** Demographic and other characteristics of transmitting and recipient partners^1^.

	Transmitting partners (*n* = 25)	Recipient partners (*n* = 25)	Partners for whom direction of transmission could not be determined (*n* = 12, 6 partner-pairs)
	*n* (% or IQR)	*n* (% or IQR)	*n* (% or IQR)
Sex			
Male	24 (96%)	25 (100%)	12 (100%)
Female	1 (4%)	0	0
Median Age	29 (24–33)	30 (25–36)	30 (27–32)
Ethnicity			
Alaska Native/American Indian	0	1 (4%)	0
Asian/Pacific Islander	2 (8%)	0	0
Black/African American	0	1 (4%)	0
White	18 (72%)	20 (80%)	10 (83%)
Multiracial	1 (4%)	3 (12%)	1 (8%)
Unknown	4 (16%)	0	1 (8%)
Hispanic	1 (4%)	2 (8%)	1 (8%)
HIV risk			
MSM	23 (92%)	23 (92%)	12 (100%)
MSM and IDU	1 (4%)	1 (4%)	0
Heterosexual	1 (4%)	1 (4%)	0
Days from HIV infection in recipient to specimen collection			
Plasma	22 (−1–50)^2^	22 (13–33)^3^	98 (77–124)^4^
PBMC	23 (2–57) ^5^	24 (19–41)^6^	164 (97–173)^7^
At the time of transmission			
Duration of infection (days)	336 (82–1,219)	(n/a)	(n/a)
CD4+ T-cell count	514 (372–618)	(n/a)	(n/a)
HIV-1 RNA level	21,558 (5483–57,733)	(n/a)	(n/a)
ARV history			
Naïve	15 (60%)	(n/a)	(n/a)
Treated in past	6 (8%)	(n/a)	(n/a)
Ongoing^8^	2 (8%)	(n/a)	(n/a)
Unknown	2 (8%)	(n/a)	(n/a)

**Abbreviations:** ARV, antiretroviral; IDU: injection drug use; MSM, men who have sex with men; n/a: not applicable.

^1^This analysis includes partner-pairs B–K from Truong et al.[32]. In the current analysis, we used different methods to determine linkage of infection. In this analysis, Truong partner-pair I was classified as linked and Truong partner-pair A was not confirmed as linked.

^2^ *N* = 20

^3^ *N* = 24

^4^ *N* = 8

^5^ *N* = 21

^6^ *N* = 19

^7^ *N* = 10

^8^HIV transmission is estimated to have occurred concurrent with ARV start in the transmitting partner.

454-pyrosequencing was conducted on an estimated median of 1011 (range 129–7550) HIV-1 templates from plasma and PBMC specimens. The average substitution error rate in the plasmid controls across all plates was 0.081+/- 0.041%. Only substitutions present at frequencies ≥1% in both the forward and reverse reads were considered for analysis and are shown in **Tables 2 and 3**.

**Table 2:** Frequency of mutations conferring at least low-level HIV-1 drug resistance detected by 454-pyrosequencing at levels >1% in partner-pairs for whom the directionality of transmission could be determined.

Pair # (PIC ID#) Transmitter/ Recipient	Transmitter ARV history?	Mode of HIV Acquisition^1^		Transmitter			Recipient		
			Codon	Percentage plasma/PBMC	Days^2^ plasma/PBMC	DNA load^3^	Percentage plasma/PBMC	Days^2^ plasma/PBMC	DNA load^3^
1 (11473/90770)	Naïve	cRAI	G73S	0**/1.1**	3/3	830	0	11/NA	
2 (55751/11286)	Treated in past	cRAI	K101E	0/**3.6**	−10/−10	204	0/0	14/19	404
			M184V	0/**2.0**			0/0		
			M184I	0/0.3			0**/2.4**		
			G190A	0/**6.0**			0/0		
			G73S	0**/1.1**			0.2**/1.1**		
3 (15620/25807)	Naïve	cRAI	T215A	**1.6**/NA	22/NA		0/0	22/22	210
			G73S	0/NA			0**/1.7**		
4 (89028/78569) Truong #B^4^	Treated in past	cVI	M184V	0.9/**11.8**	66/29	203	0/0.7	19/36	1433
7 (58368/87014) Truong #E^4^	Treated in past	cRAI	M184I	0/**1.5**	50/22	2226	0/0	8/16	6379
			V82T	0/**2.8**			0/0		
9 (41033/88787) Truong #H^4^	Naïve	cRAI	K101E	0/0	12/26	110	0/**5.3**	39/41	362
10 (26486/56710)	Naïve	cRAI	M41L	**97.8/99.7**	72/94	404	**99.5/99.8**	72/54	1013
Truong #I^4^			K70R	0/0			0**/1.1**		
			L74V	0/0			0**/1.1**		
			M184I	0/0.3			0**/4.2**		
			T215D	**97.1/99.3**			**99.7/99.8**		
12 (30627/72526) Truong #K^4^	Treated in past	cRAI^5^	D67N	0.2/**1.0**	70/65	2035	0/0	58/42	2262
19 (29244/53653)	Naïve	cAI	Y181C	**99.2/99.1**	111/111	49	**98.7/99.7**	37/37	722
20 (52647/90629)	Ongoing^6^	cRAI	K103N	**99.8/99.2**	−20/−20	117	**98.1/99.8**	52/52	497
			M184V	**97.5/95.3**			**38.2/67.9**		
22 (15332/51861)	Naïve	cRAI	G73S	0.4/NA	35/NA	NA	0**/1.6**	19/14	NA
24 (57604/40119)	Naïve	cRAI	Y181C	**99.9/99.2**	21/21	138	**99.8/99.1**	21/21	482
			K219Q	0/**1.3**			0/0		
25 (39522/99203)	Naïve	cIAI	M41L	NA/**99.0**	NA/57	4	**99.9/99.9**	27/27	NA
			M184I	NA/0			0**/3.7**		
			T215C	NA/**95.1**			**98.4/98.6**		
			D30N	NA/**98.6**			**99.9/100**		
			N88D	NA/**98.7**			**99.8/99.0**		
32 (20735/90114)	Naïve	cRAI	M184I	NA/**2.5**	NA/−41	10567	0/NA	42/NA	
			G73S	NA/**1.5**			0/NA		

**Abbreviations:** ARV, antiretroviral; cIAI, condomless insertive anal intercourse; cRAI, condomless receptive anal intercourse; cVI, condomless vaginal intercourse; NA, not available; PBMC, peripheral blood mononuclear cell.

Bold font indicates a mutation found at ≥1.0%.

^1^Refers to likely mode of HIV acquisition for the recipient partner

^2^Days from estimated date of transmission to specimen collection (plasma/PBMC); In pairs for whom the direction of transmission was determined, this was the estimated date of transmission in the recipient. In pairs with unknown direction, this was estimated as the earliest possible HIV acquisition in the pair.

^3^HIV-1 DNA Copies/10^6^ PBMC

^4^IDs from Truong et al [32]

^5^MSM/IDU but pair reported no sharing of needles or other works

^6^HIV transmission occurred concurrent with ARV start in the transmitting partner; specimens for pyrosequencing were obtained prior to this date.

**Table 3:** Frequency of mutations conferring at least low-level HIV-1 drug resistance detected by 454-pyrosequencing at levels >1% in partner-pairs for whom the direction of transmission could not be determined.

Pair # (PIC ID#) Transmitter/Recipient	Mode of HIV Acquisition		Partner A			Partner B		
		Codon	Percentage plasma/PBMC	Days^2^ plasma/PBMC	DNA load^3^	Percentage plasma/PBMC	Days^2^ plasma/PBMC	DNA load^3^
5 (97101/82323)	MSM	M184I	0.2/0	90/91	NA	0/**2.6**	97/97	10
Truong #C^4^		G73S	**1.0/1.2**			0/**2.0**		
29 (34287/26803) Truong #G^4^	MSM	Y181C	0.1/**1.5**	203/203	349	NA/0	NA/205	476
30 (25521/68398)	MSM	M184I	0.2/**1.1**	103/103	2420	0.1/0.1	98/97	599

**Abbreviations:** ARV: antiretroviral; IDU, injection drug use; PBMC: peripheral blood mononuclear cell; NA: not available; MSM: man who has sex with men.

Bold font indicates a mutation found at ≥1.0%.

^1^Refers to likely mode of HIV acquisition for the recipient partner

^2^Days from estimated date of transmission to specimen collection (plasma/PBMC). In pairs for whom the direction of transmission was determined, this was the estimated date of transmission in the recipient; in pairs with unknown direction, this was estimated as the earliest possible HIV acquisition in the pair.

^3^HIV-1 DNA Copies/10^6^ PBMC

^4^IDs from Truong et al [32]

^5^MSM/IDU but pair reported no sharing of needles or other works

^6^HIV transmission occurred concurrent with ARV start in the transmitting partner; specimens for pyrosequencing were obtained prior to this date.

In 25 partner-pairs where directionality of transmission could be determined, 12 (48%) transmitters had 1–4 drug resistance mutations (total = 23) detected at ≥1% of their viral population at a median frequency of 6.0% (IQR 1.5%–98.7%, range 1.0%–99.6%) (**Table 2**). Eleven (48%) of the 23 mutations detected in transmitting partners were also detected in the recipient partner. Ten of these mutations were both major mutations and majority variants present in the HIV-1 population of ARV-naïve transmitters at frequencies greater than 95%; 100% of these majority variants were detected in the recipient (95% CI, 69.2%–100%). All of these mutations were identified in transmitting partners who were ARV-naïve at the time of specimen collection, although one transmitter followed in the UWPIC cohort (PIC52647) later initiated ARVs around the estimated date of transmission. When we included partner-pairs for whom the directionality of transmission could not be determined with certainty (**Table 3**), there were between 14 and 17 mutations present as minority variants in the transmitter, with only one or two of these identified in plasma (depending on the direction of transmission in partner-pair #5). Four of these transmitters had previously received antiretroviral therapy. These minority variants in the confirmed or possible transmitter included between eight and 11 major and six accessory mutations and ranged from 1.0%–11.8% of the viral population. Two (14%, 95% CI, 1.8%–42.8%) G73S accessory mutations were detected in the PBMCs of the recipient at a level of 1.1% and either 1.0%/1.2% or 2.0%, depending on who was considered the transmitter and recipient among partner-pair #5. When major mutations were present in the transmitter as minority variants, none of these mutations was observed in the recipient partner.

If drug resistance does not impact “transmission fitness,” then the probability of transmission may be dependent on the frequency of the variant in the viral population of the transmitter (e.g., if a virus exists as 10% of the viral population, it would have a 10% chance of transmission, assuming that all infections were caused by a single founder virus). If this were true, based on the observed frequencies of drug-resistant viruses in transmitting partners, the likelihood that two or more minority variants would have been identified in recipient partners by chance alone ranges from 5.9% to 8.0% (depending on which partners are considered transmitter and recipient among the partner-pairs in which the direction of transmission could not be determined with certainty). These values are less than our observed point estimate of 14% but fall within the 95% CI.

In the first of our post-hoc analyses, in which we considered only the 25 partner-pairs in which the direction of transmission could be determined and used the 1% threshold, we observed one G73S in both the transmitter and recipient partner out of 13 low-frequency mutations identified in known transmitters (7.7%, exact 95% CI 0.19%–36.0%), with an expected likelihood of identifying one or more mutations in recipients of 32.3%. When we considered the 25 confirmed partner-pairs and low-frequency mutations present at levels ≥2% of the viral population in the transmitter, we observed no mutations in the recipient partner that had been identified in the transmitting partner (0%, exact 95% CI 0%–45.9%), with an expected frequency of observing one or more mutations of 25.8%.

Furthermore, among the 25 partner-pairs where directionality could be determined, seven recipient partners had a total of nine low-frequency mutations identified at a median concentration of 2.0% (IQR 1.4%–4.0%, range 1.1%–5.3%) that were not detected in plasma or PBMCs of the transmitter; all of these mutations were identified in the PBMCs of the recipient. The majority (92.6%) of all 27 low-frequency mutations identified in our study were detected in PBMCs, and many were consistent with APOBEC mutations (e.g., M184I and G73S) or regions of homopolymers, such as K101E and K219Q (**Tables 2 and 3**).

**Fig 1:**
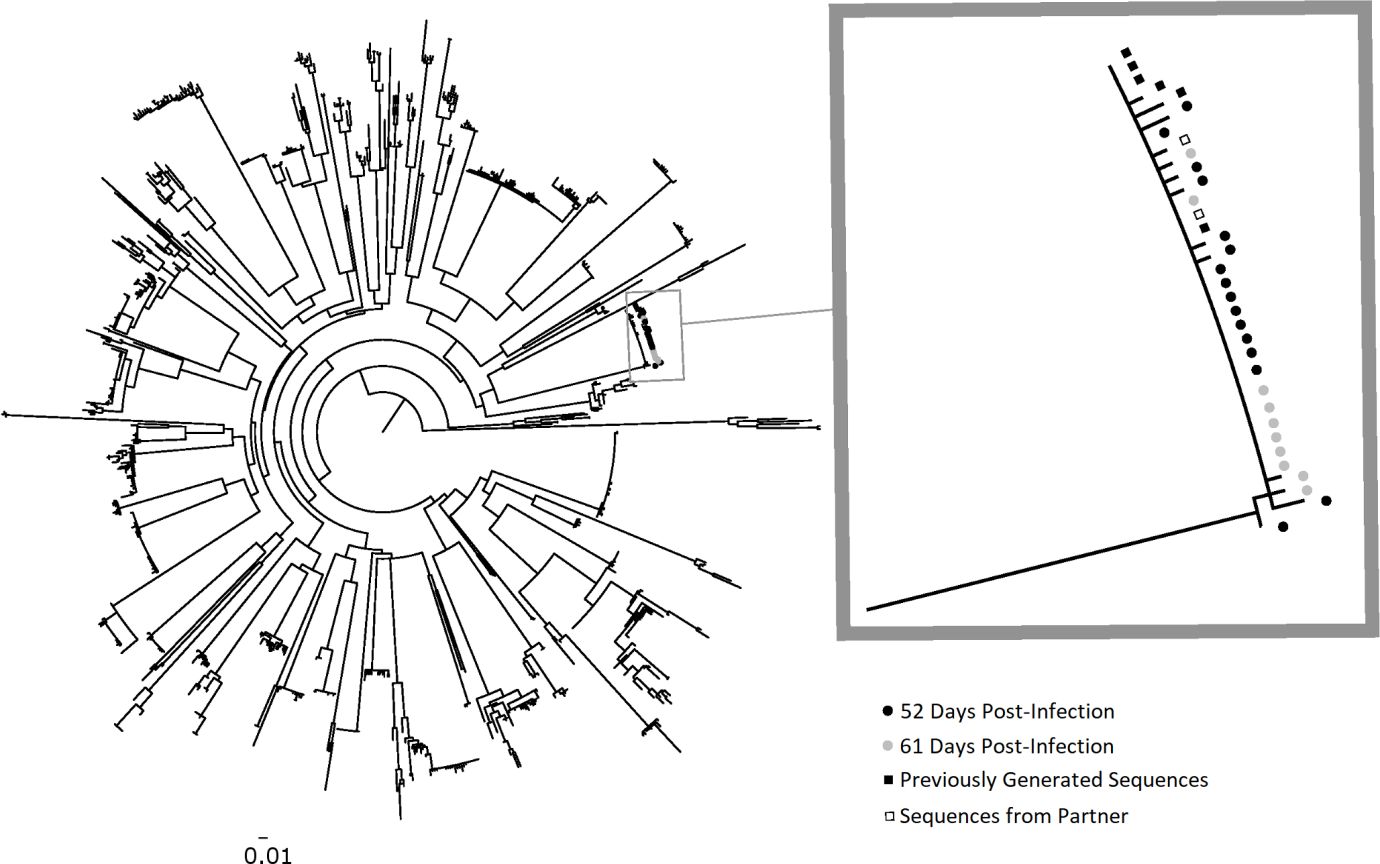
Phylogenetic analysis of HIV-1 *env* sequences from longitudinal specimens from PIC subject 90629. The first available specimen from PIC 90629 was observed to have a dual HIV *pol* population, with approximately 50% of viral variants resistant to lamivudine/emtricitabine (M184V) and 50% drug-susceptible (see Table 2). To determine if the dual population resulted from two founder populations, the HIV-1 *env* C2–V5 region was analyzed for viral diversity using plasma specimens collected at 52 (black circles) and 61 (gray circles) days post-infection. A total of 17 and 10 sequences were derived from these specimens, respectively, by SGA. Maximum-likelihood phylogenetic analysis with sequences from other PIC participants revealed PIC 90629’s sequences from both timepoints comprised a near-homogeneous monophyletic cluster with sequences previously generated from a separate aliquot of the day 52 specimen (black squares) and from his transmitting partner (open squares). The absence of two discrete viral populations suggests that his infection was established with a single HIV-1 *env* variant. The nearly equivalent mixture of genotypes at codon 184 in *pol* encoding reverse transcriptase may represent reversion of drug-resistant to -susceptible virus within the recipient. The 1 or 2 base pair differences in longitudinal HIV-1 *env* C2–V5 sequences likely resulted from reverse transcription errors in the recipient or PCR error and do not reflect infection by multiple variants. The scale bar (horizontal line) indicates the number of substitutions per site. PIC, Primary Infection Cohort; SGA, single genome amplification.

The observed frequencies of M184M/V in PIC90629 raised the possibility that at least two HIV variants founded infection in this individual. Longitudinal analysis of PIC90629 by SGA confirmed 454-pyrosequencing results and identified a mixture of M184M/V. At 52 and 61 days post-infection, 8 of 21 (38.1%) and 5 of 9 (55.6%) *pol* sequences from plasma contained the nonsynonymous M184V mutation, respectively. The *env* sequences from these timepoints clustered into one monophyletic group (**Fig 1**). Although not definitive, this suggests that only one viral variant was transmitted and that the drug-susceptible variant may have been generated by a random point mutation in the recipient.

## Discussion

While many studies have investigated the consequences of minority variants following interventions to prevent mother-to-child transmission [33–35], the results described here represent, to our knowledge, one of the first cross-sectional surveys to evaluate the detection of minority and majority HIV-1 drug-resistant variants among epidemiologically linked and phylogenetically confirmed partner-pairs from specimens collected around the time of sexual HIV transmission. In this analysis of HIV transmission events among 31 partner-pairs, most of the drug-resistant mutations detected were either at frequencies close to 100% of the viral population or at frequencies below 10%. All high-frequency mutations were shared between partners, and two low-frequency mutations were seen in each partner of two sets of partner-pairs. If all of the variants seen in recipient partners were indeed transmitted, this would be counter to prior studies that suggested that HIV-1 drug resistance confers reduced “transmission fitness.” However, the identification of high- and low-frequency mutations in recipient partners may represent different phenomena. High-frequency mutations transmitted from ARV-naïve partners likely represent transmission chains of drug-resistant variants, whereas there are alternate explanations for the presence of the G73S accessory mutation as minority variants in both transmitters and recipients. These mutations, as well as about half of the other minority variants seen in our study, could be due to the generation of variants with limited capacity to replicate and transmit by the ARV restriction factor APOBEC-3G [19]. In either case, we provide evidence that drug-resistant variants were detected in recipient partners with similar, if not higher, frequency than predicted by their presence in transmitting partners.

The strong association between the frequency of the viral variant in the transmitter and the likelihood of detection in the recipient supports the concept that replication capacity is associated with the likelihood of sexual transmission [36], as has been reported with mother-to-child transmission [37]. A person who has previously received ARV therapy is likely to transmit a drug-resistant variant if that variant remains the predominant strain in the individual’s viral population.

Our findings also suggest that persons with primary HIV-1 infection may account for a large proportion of transmitted drug resistance because of their disproportionate contribution to incident infections [38] and the small chance that transmitted majority variants will revert or be overgrown by drug-susceptible virus prior to onward transmission, either because the transmitted major mutation has minimal impact on replication capacity [39] or because infection is most often caused by a single founder virus, minimizing the chance that even variants with reduced replication capacity would be overgrown. Notably, one of the population-based models that suggested that drug resistance reduces “transmission fitness” found that drug-resistant viruses were equally likely to be transmitted as drug-susceptible viruses when the model was adjusted to increase the estimated proportion of transmission from persons with primary HIV-1 infection [6].

Our study has several limitations in addition to the relatively small numbers of transmitters identified to have major mutations present as minority variants. The specimens evaluated were collected, on average, nearly a month after transmission, with some specimens collected months after the estimated date of transmission. A prolonged interval between the transmission event and specimen collection could have resulted in overgrowth of minority variants by drug-susceptible virus, evolution of new mutations in recipient partners, or accumulation of mutations due to APOBECs; our results might have differed if we had collected specimens closer to transmission. Our results might also have differed if we had analyzed genital tract specimens from transmitters, although there is moderate concordance between drug resistance in blood and semen [40–42]. Our methods did not rigorously assess the number of HIV-1 variants sequenced, which could have been accomplished by use of Primer IDs [43]. Our control plasmid was not diluted to the same concentration as participants’ specimens and therefore may have underestimated sequencing errors. While we assessed the input number of amplifiable templates, we evaluated this by amplification of a relatively short region of *gag* and might have overestimated the templates evaluated and artificially inflated our estimates of minority variants. However, we used primers designed to anneal to highly conserved regions to minimize this effect. Our analyses did not account for the rate of random reverse transcription errors in the participant. Our results differed when we excluded partner-pairs where the direction of transmission could not be determined and when we used a threshold of 2%; in both cases, the observed frequency of minor variants in recipient partners was less than predicted based on the presence of variants in the transmitter, but confidence intervals around the observed frequency were wide and again included the expected number in both cases. Our results might also have differed had we used a lower cutoff to determine drug resistance. Finally, our statistical analysis did not account for genetically linked mutations or the possibility of multiple founder viruses, and numbers were too small to perform regression analyses to account for multiple mutations in the transmitter, evaluate the likelihood of identification of specific mutations, or identify other factors (e.g., demographic characteristics, HIV-1 RNA levels, or duration of infection) that might be associated with transmission of drug-resistant variants.

Controversy remains as to whether minority variants detected in recently infected persons are transmitted, especially given that almost all heterosexual transmission and most male–male sexual transmission leads to infection with a single founder variant [16–18]. Allele-specific PCR results from one study provide indirect evidence of transmission of minority drug resistance [15], and the authors suggest that their findings were due to transmission of multiple variants. Alternatively, given that G-to-A changes caused over half of minority mutations we identified and that most of these were in PBMCs, it is possible that some errors could have been introduced by APOBECs and arose de novo in both transmitters and recipients, or that other errors occurred during pyrosequencing or by misincorporation of nucleotide bases by the viral polymerase. Additional studies are needed to elucidate our findings.

On a population level, the proportion of HIV-1 transmission from persons with acute HIV-1 infection remains unclear, and recent work suggests that the rate of transmitted drug resistance may vary over calendar time along with both the proportion of transmission from persons with acute infection and with the availability of new antiretroviral therapies that more effectively suppress viremia and, therefore, transmission [44]. Strategies to decrease the incidence of transmitted drug resistance should continue to focus on increasing adherence and using ARV medications with high genetic barriers to resistance to limit selection of drug resistance among persons receiving ARV therapy. Potentially as important is work to increase the recognition of persons with acute HIV-1 infection, which might have a heretofore unrecognized impact on transmitted drug resistance if transmission chains can be interrupted by behavioral change or early ARV therapy.

## Supporting information

S1 STROBE Checklist STROBE checklist. (DOC)

S1 Text Grant application. (DOC)

## Abbreviations

APOBEC: apolipoprotein B mRNA-editing enzyme, catalytic polypeptide
ARV: antiretroviral
cIAI: condomless insertive anal intercourse
cRAI: condomless receptive anal intercourse
cVI: condomless vaginal intercourse; env, envelope
HIV-1: human immunodeficiency virus type 1
IDU: injection drug use
MID: Multiplex Identifier
MSM: men who have sex with men
NNRTI: non-nucleoside reverse transcriptase inhibitor
NRTI: nucleoside reverse transcriptase inhibitor
PBMC: peripheral blood mononuclear cell
PI: protease inhibitor
pol: polymerase gene
SGA: single genome amplification
UWPIC: University of Washington Primary Infection Clinic
