# Supplementary material for: Transmission of HIV-1 drug resistance mutations within partner-pairs: A cross-sectional study of a primary HIV infection cohort
Source: PLoS Med. 2018 Mar 27;15(3):e1002537. doi: 10.1371/journal.pmed.1002537 (PMC5870941; doi:10.1371/journal.pmed.1002537)
Supplement: S1 Text — (DOC) [file pmed.1002537.s002.doc]

**1. Introduction to Application**

This is a revision of an R01 application that was initially submitted September 7, 2007 and was re-submitted January 8, 2008. The overarching goal of this project is to define the natural history and clinical significance of transmitted low-frequency human immunodeficiency virus type 1 (HIV-1) drug resistance mutations using the oligonucleotide ligation assay, OLA.

The Summary of Discussion from March 5, 2008 relays varying degrees of enthusiasm among reviewers for our proposal. Among the scientific community, there is a clear and growing interest in understanding the relevance of low-frequency HIV-1 drug resistance, and a few studies have been presented or published since the last submission [2-6]. However, the aims of this application remain highly novel. In fact, the editors' summary and the editorial by Dr. Steven Deeks that accompany the most recent publication on this topic state "It remains to be determined if the prevalence of these presumably transmitted mutations will wane with time" and "…because data on minority drug-resistant viruses are limited, more studies - particularly with recent populations - are needed before testing for these variants can be considered appropriate in the clinical management of newly diagnosed HIV infection" [5, 7]. We concur. These are exactly the studies proposed in Aims #1 and #2 of this application.

It was "unclear to reviewers whether the body of data that would be produced by this study would be able to alter treatment of people who are acutely infected with HIV."

Although our data would not answer the question whether ARV therapy should be initiated during acute HIV-1 infection, it will help to answer other questions relevant to the care of persons with acute HIV-1 infection. If low-frequency mutations negatively impact the response to ARV therapy, use of more-sensitive assays should be considered at the time of HIV-1 diagnosis. If low-frequency mutations negatively impact the response to ARV therapy but this effect is attenuated by an increasing time interval between HIV-1 acquisition and the start of ARV therapy, treatment during acute HIV-1 infection should be deferred (given that the current data show no clear benefit of immediate treatment on long-term clinical outcomes).

"Reviewers are interested and excited about aim three, the partner study, but want to know the proportion of subjects that present with drug resistance mutations acquired from their partner."

We share the enthusiasm of the reviewers regarding Aim #3. While this is a particularly exciting area of our research, it is important to note that this analysis is inextricably linked to the other project aims. For example, because we cannot study partners at the exact moment of HIV-1 transmission, it is essential for us to determine the duration that low-frequency mutations are detected over time following HIV-1 acquisition (Aim #1). If the source and recipient partners do not have the same pattern of drug resistance mutations, data on the "persistence" of low-frequency mutations (i.e. whether the "prevalence of these presumably transmitted mutations will wane with time") will help us estimate whether the different patterns we observe were actually due to the lack of transmission of mutant virus from the source to recipient partner or due to the overgrowth of the low-frequency mutations by wild-type viruses in the recipient partner in the interval between HIV-1 acquisition and study enrollment.

To our knowledge, no data currently exist to answer "the proportion of subjects that present with drug resistance mutations acquired from their partner" as the only source partners who have had resistance testing performed to date were those who were also enrolled during primary HIV-1 infection. We are also unaware of any other studies that have compared detailed HIV-1 drug resistance patterns among large numbers of partner-pairs. Additional information is provided in **4.h The PIC Partners study** and **Table 7**. We presume that all transmitted HIV-1 drug resistance in ARV-naïve subjects was acquired from the confirmed source partners. Three confirmed source partners were already at least partially studied because the source partners were also identified during primary HIV-1 infection; transmitted HIV-1 drug resistance was identified in two of three source partners. In the first pair, OLA identified three mutations in the probable source (K65R, T215Y, K103N) but only two in the probable recipient (K65R and T215Y); in the second pair, consensus sequencing identified two mutations in the source (M184V and K103N) but only K103N in the recipient partner. Of the recipient partners fully or partially studied to date, 36% had transmitted HIV-1 drug resistance, and so the proportion of source partners who had HIV-1 drug resistance at the time of the transmission event must therefore be at least 36%. The quantification of the transmission of HIV-1 drug resistance from source to recipient partner is the objective of this aim.

"Some reviewers are concerned that though a small cohort has been studied to date, the data presented in the application has results pending. It is also a concern whether sufficient study subjects that remain as partners will be able to be recruited to provide sufficient power…"

At the recommendation of reviewers, we have deferred this resubmission until our pilot project was completed; the findings are not significantly different from what we previously described. The overall prevalence of transmitted HIV-1 drug resistance is somewhat lower (34% versus 46%) because subjects were studied in reverse chronologic order, and those recently studied were among those enrolled prior to 2000. However, we remain confident that the primary objectives of each of the aims will be sufficiently powered with continued enrollment of subjects with acute HIV-1 infection and their putative source partners during a planned five-year study period, as described in detail in **5. Research Design and Methods.**

Other concerns of reviewers are addressed below. Changes to the submission are indicated by italicized text.

Critique #1:

1. "…the proposed research … would not likely affect treatment options of HIV infected individuals." and "Most importantly, is this study relevant to clinical significance as patients can be diagnosed to be in virological failure and function normally with the diagnosis of acute HIV infection."

As mentioned above, results from these studies could modify current treatment practices. We agree with Reviewer #1 that low-frequency mutations may not, in fact, uniformly reduce the efficacy of ARV therapy, and there may be negative consequences if clinicians unnecessarily avoid effective ARV agents in favor of more complicated regimens. It also should be determined, even if low-level mutations are clinically relevant, if use of more-sensitive HIV-1 drug resistance assays prior to initiation of ARVs would be cost-effective.

We do not share the opinion of Reviewer #1 that virologic failure is of little consequence. Most experts would agree that it is not advisable to allow persons on ARVs to experience virologic failure. Individuals who acquire HIV-1 infection in 2008 are predicted to live for decades following infection, much of the time while receiving ARV therapy. Although several highly effective agents from different ARV classes were recently approved, the rapid development, for example, of the M184V mutation completely eliminates the ability to construct a standard-of-care ARV regimen that is once-a-day and easily tolerated. Increasing pill burden, dosing frequency, and/or increased medication side effects from subsequent regimens would reduce adherence and the chance of virologic success. Thus the initial virologic failure is not without long-term consequences, whether it is in acute or established HIV-1 infection.

2. "The partner studies proposed … addresses an important area of research, but the number of subjects studied to [date] is small and it is still unclear whether [there will be] an adequate number of subjects to adequately power the studies."

In **5.c.3** we explain in detail how the combination of our recruitment record, the likelihood of confirming putative partners, and the likelihood that source partners would have HIV-1 drug resistance will achieve a sample size that has adequate power for the proposed analyses. "… we will have 82% power to detect a transmission probability of all source partner mutations of 80% or less if we have 17 confirmed partner-pairs in which the source partner has at least one HIV-1 drug resistance mutation…" We would need to enroll 0-21 putative source partners in order to have a total of 17 confirmed partner-pairs in which the source partner has at least one drug resistance mutation. Notably, three partner-pairs have enrolled in the last six months. With continued enrollment, we will have enrolled the maximum number of needed partner-pairs by the end of year five.

3. re: Aim 1: "It is unclear how the determination that low-frequency mutations are rapidly lost to detection would support drug resistance testing at the time of entry into care."

Guidelines currently recommend HIV-1 drug resistance testing at the time of entry into care [8] even though transmitted mutations detected by consensus sequencing continue to be detected for years following HIV-1 acquisition [9]. HIV-1 surveillance activities of Public Health - Seattle & King County incorporate resistance testing into the care continuum at an even earlier time point, using remnant sera from the initial positive HIV-1 antibody test for consensus sequencing. Results are then communicated to providers of clinical care. If data indicate that low-frequency HIV-1 drug resistance mutations are clinically relevant and are rapidly lost to detection and if more-sensitive assays are developed for clinical use, our public health activities might be tailored to look for low-frequency mutations instead of mutations detected by consensus sequencing.

4. "Also, the PI is urged to be creative in obtaining more subjects since power is a question."

Details in **5. Research Design and Methods** explain that all of the primary objectives are adequately powered with the anticipated sample size. Depending on results of these studies, future grant applications could, for example, generate collaborations with other cohorts of partner-pairs in order to study the transmission of specific mutations (e.g. K103N) in greater depth.

5. "Specifically, the public health importance of the data that would be obtained is still not clear."

In our view, the proposed studies are highly relevant to public health and represent an interface between public health and HIV-1 clinical research and care. Explicitly, if more-sensitive assays are endorsed without a better understanding of the impact of low-frequency mutations (Aim #2), the detection of minority variants might lead care providers to avoid prescribing the convenient first-line ARV regimens that have fewer pills and less-frequent dosing. A greater complexity of ARV regimens could reduce patient adherence and lead to a paradoxical increase in the prevalence of drug resistance. If minority variants are clinically relevant, knowing the duration that these mutations may be detected (Aim #1) would support recommendations for HIV-1 drug resistance testing at entry to care and should lead public health departments to consider incorporating drug resistance testing into HIV-1 confirmatory testing and public health surveillance programs [10]. Furthermore, understanding the true prevalence of resistance to different classes of ARV medications would help care providers to select appropriate empiric ARV regimens for post-exposure prophylaxis (PEP) or for the treatment of acute HIV-1 infection."

Critique #2

1. "Although the oligonucleotide ligation assay (OLA) is more sensitive than consensus assays, there are several novel assays which can detect variants at a frequency of 1% of less. The rationale for using the OLA assay - that more sequences can be sampled - is undercut by the central question of the application - the import of transmitted minor variants over time in the newly infected patient."

We agree with Reviewer #2 that there are other more-sensitive assays, but polymorphisms hamper these assays to a greater degree than OLA. Furthermore, extremely sensitive assays may at times be so sensitive as to detect the low background levels of random mutations that were described in HIV-infected populations even prior to the first use of ARVs in the 1980's. For this reason, although the real-time PCR assay used by Johnson et al [5] can detect HIV-1 drug resistance in as little as 0.001% of the viral quasi-species, the authors used cut off levels between 0.4-2.0% (similar to the sensitivity of OLA) for their analyses.

Critique #3

1. "While history of exposure to PrEP or PEP is a very important variable, exclusion of these subjects may be an error, particularly if these prevention modalities become commonly used."

Although PEP and PrEP have been used by a limited number of individual providers, ongoing randomized clinical trials (RCTs) have just begun and widespread adoption is likely several years away if these RCTs show positive results and funding can be identified for implementation. At the suggestion of this reviewer, we will plan to enroll and study these subjects, but we will analyze their data separately.

2. "A biohazard comment was noted in the prior critique and a description continues to be lacking."

We believe that Reviewer #3 refers to a prior comment by Reviewer #2. Additional text was added to **7.b.2 Protection against risk** documenting the biohazard training that clinical and laboratory staff receives. Further details on this training and procedures are available through the UW Biosafety Manual (online at: http://www.ehs.washington.edu/rbsbiosafe/sectioniv.pdf)

3. "…the study section believes that the focus on aim 3 should be able to be completed in 3 years and recommend that the time and budget be reduced to 3 years."

As outlined in **5.g Timetable for all projects**, the laboratory work to be performed for this project will commence with specimens identified for Aim #3, and preliminary results are likely to be generated within the first two years. However, because the additional sample size for all aims will require additional enrollment, shortening the proposal to fewer than five years is not feasible and risks the success of these studies.

Public health surveillance programs using consensus sequencing (genotyping) estimate that at least one in ten antiretroviral (ARV)-naïve persons infected with HIV-1 in the United States acquires drug resistant HIV-1. Guidelines therefore recommend resistance testing at the time of entry into care. However, consensus sequencing cannot detect low-frequency variants at levels below 10-50% of the viral population. The oligonucleotide ligation assay (OLA) is a more-sensitive assay that can detect mutations occurring in as little as 5% of the viral quasi-species. In a pilot study conducted among subjects with primary HIV-1 infection enrolled at the University of Washington Primary Infection Clinic (PIC), consensus sequencing detected transmitted HIV-1 drug resistance in *6% of 100* subjects, and OLA detected low-frequency mutations in *28 (30%) of 94* subjects who did not have mutations identified by consensus sequencing.

We propose studies that will use OLA to address questions pertaining to the transmission and subsequent consequences of HIV-1 drug resistance. In **Aim #1**, we will study ARV-naïve PIC subjects to compare the duration of detection ("persistence") and level of detection of transmitted HIV-1 drug resistance over time in peripheral blood mononuclear cells (PBMCs), blood and seminal plasma. In **Aim #2**, we will study PIC subjects initiating ARV therapy and use OLA to determine whether additional mutations can be detected in PBMCs during successful treatment due to the selection of transmitted low-frequency drug resistance mutations or the development of new mutations. In **Aim #3**, we will use OLA to compare HIV-1 drug resistance patterns in PIC subjects and their source partners to determine whether HIV-1 drug resistance impacts "transmission fitness".

These novel investigations would broaden our understanding of the natural history and clinical impact of low-frequency HIV-1 drug resistance and inform guidelines for the testing and treatment of HIV-infected persons. If more-sensitive HIV-1 drug resistance assays were to be endorsed for clinical care before there is a full understanding of the relevance of low-frequency mutations, the potential increase in the complexity of initial ARV regimens and subsequent reduction in patient adherence could paradoxically increase the prevalence of drug resistance. Finally, empiric data from partner-pairs will generate information on correlates of HIV-1 transmission that could be incorporated into future models of the population dynamics of drug resistance. These models would estimate the overall proportion of HIV-1 drug resistance that is transmitted from ARV-naïve source partners with primary HIV-1 infection versus ARV-experienced source partners with established HIV-1 infection. This data could be used to design public health interventions targeted to these populations to reduce the spread of transmitted HIV-1 drug resistance.

**2. Specific Aims**

Reports of transmitted HIV-1 drug resistance are increasing worldwide. In Seattle, Washington, 11% of ARV-naïve persons have HIV-1 drug resistance detected by consensus sequencing [10], and this estimate is likely to be conservative because consensus sequencing cannot detect low-frequency viral variants [11-16]. In a pilot project at the University of Washington Primary Infection Clinic (PIC), low-frequency HIV-1 drug resistance was detected by the oligonucleotide ligation assay (OLA) in *28* (*30%) of 94* subjects with primary HIV-1 infection who had no mutations detected by consensus sequencing. *Our understanding of transmitted HIV-1 drug resistance is superficial and incomplete.* We have designed studies that will better define the clinical and public health implications of low-frequency HIV-1 drug resistance mutations. We propose the following specific aims:

**Aim #1: Determine whether the "level" of detection of HIV-1 drug resistance variants predicts the duration of detection of these variants over time since HIV-1 acquisition.**

Rationale: Studies using consensus sequencing have shown that majority variants are detected for years after HIV-1 acquisition [9, 17-25], but we could find no corresponding studies of low-frequency variants. Whether transmitted mutants continue to be detected ("persist") or are overgrown by wild-type viruses may be related to their levels at the time of HIV-1 acquisition, which may vary in PBMCs, blood, and seminal plasma [26, 27].

Methods: We will use OLA to quantify the level (<5%, 5-10%, 10-30%, 30-50%, and >50%) of mutant variants in ARV-naive subjects in the PIC cohort at baseline (the first available specimens) and 6 and 12 months after infection. Analyses will compare the detection of mutations in PBMCs compared to blood and seminal plasma and will evaluate the association between the baseline level of mutant variants and rate of continued detection.

Hypothesis: A greater proportion of untreated subjects will have mutations that remain detectable in PBMCs during follow-up compared to blood and seminal plasma, and a smaller proportion will have low-frequency mutations that remain detectable compared to subjects with mutations detected by consensus sequencing.

Implications: If *clinically-relevant* low-frequency mutations are rapidly lost to detection, these data would support the use of HIV-1 drug resistance tests at the time of entry into care and should lead public health departments *not only to expand HIV-1 testing programs but also* to consider incorporating drug resistance testing into HIV-1 confirmatory testing and public health surveillance programs.

**Aim #2: Quantify the increase in detection of low-frequency mutations in PBMCs during ARV therapy.**

*Rationale:* Transmitted low-frequency mutations have been associated with poor treatment outcomes in some studies [2, 3, 5, 6, 28, 29], but *others have found no association between minority variants and poor response to ARVs* [4]. HIV-1 replicates at low levels in PBMCs during ARV therapy [30, 31], and this may result in the selection or development of HIV-1 drug resistance mutations over time [23, 32-34]. Persons with low-frequency drug resistance who receive ARV therapy may be at greater risk for virologic failure if levels of drug resistance mutations increase in PBMCs at a higher rate than in persons without drug resistance at baseline.

*Methods:* We will use OLA to quantify the proportion of subjects in the PIC cohort who have *new* low-frequency mutations detected in PBMCs after 6 and 12 months of successful ARV therapy. We will examine whether relationships exist between changes in levels of HIV-1 drug resistance and the timing and response to ARVs.

Hypothesis: Over time, additional mutations will be detected in a greater proportion of treated subjects with transmitted drug resistance compared to treated subjects without HIV-1 drug resistance detected at baseline.

Implications: If low-frequency mutations reduce the efficacy of ARV treatment, these data would support the need for clinical trials to evaluate use of more-sensitive assays to select ARVs for drug-naïve persons. Also, if levels of mutations decay prior to the initiation of ARV therapy and do not subsequently reappear during therapy, this would be a *rationale* to defer rather than initiate ARV therapy during primary HIV-1 infection.

**Aim #3: Compare HIV-1 drug resistance patterns in PIC subjects and their source partners.**

*Rationale:* Little is currently known about "transmission fitness", the ability of HIV-1 to infect susceptible hosts. Analyses suggest that drug resistant viruses may be less likely to be transmitted than wild-type viruses [35-38], but data using consensus sequencing show high concordance of mutations in partner pairs [19, 21, 25, 39, 40].

Methods: We will use OLA to compare results of HIV-1 drug resistance testing in PBMCs and plasma from PIC subjects with results of testing in PBMCs, blood and seminal plasma from their confirmed source partners.

Hypothesis: HIV-1 drug resistant variants will be less common and occur at lower levels in the PBMCs and plasma from PIC subjects than in the PBMCs, blood plasma, and genital secretions from their source partners.

Implications: *Transmission probabilities will be quantified from empiric data.* Data from Aim #3 could inform the design of public health interventions to reduce drug resistance by providing data for population models to estimate the proportion of transmitted drug resistance from ARV-experienced and ARV-naïve source partners.

**3. Background and Significance**

**3.a Definitions of primary, acute, and early HIV-1 infection**

The term primary HIV-1 infection refers to the period of time spanning approximately the first six months following HIV-1 acquisition. Primary HIV-1 infection has traditionally been subdivided into two phases by the detection of antibodies to HIV-1, but a more detailed classification system is described in Fiebig et al. [41]. In the earliest phase, called "acute HIV-1 infection", HIV-1 RNA or p24 antigen can be detected, but antibodies to HIV-1 are absent (Fiebig stages I and II). HIV-infected persons who have very recent negative HIV-1 tests or those who have positive HIV-1 antibody tests but evolving Western Blot assays (Fiebig stages III and IV) are also sometimes considered to have acute HIV-1 infection. "Early HIV-1 infection" is the term used to describe the interval between HIV-1 seroconversion and six months following HIV-1 acquisition (Fiebig stage V). Early HIV-1 infection can be diagnosed when HIV-positive individuals have either a recent negative HIV-1 test or a non-reactive test result using a less sensitive enzyme-based immunoassay (LS-EIA) [42, 43]. Subjects with acute and very early HIV-1 infection (Fiebig stages I to IV) and their source partners are the focus of this application.

**3.b The epidemiologic iceberg of transmitted HIV-1 drug resistance [44]**

Transmission of HIV-1 drug resistance has been well-documented both nationally and internationally following the widespread availability and use of ARV therapy [45-55]. In the United States, cross-sectional surveys using consensus sequencing estimate that 11-24% of ARV-naïve, HIV-infected persons have HIV-1 drug resistance [10, 52, 53, 56]. National guidelines therefore recommend resistance testing for ARV-naïve persons at the time of entry to care (A-III) and consideration of repeat testing prior to initiating ARV therapy because of the chance that individuals may have become superinfected in the interval by a different, drug resistant virus (C-III) [8].

Due to incomplete public health surveillance, it is uncertain whether the incidence of transmitted HIV-1 drug resistance has been increasing in the United States over the last decade (as reviewed in [56]), but there has been a clear increase in the number of persons identified with multi-class drug resistant (MDR) HIV-1 infection [56]. In addition, the individuals with extremely drug resistant HIV-1 infection who were recently described in New York City [39, 57] and in Seattle [10] have had very limited ARV treatment options. Given that consensus sequencing cannot detect viral variants comprising less than 10-50% of the viral population [11-16], it is likely that the prevalence of HIV-1 drug resistance and MDR HIV-1 infection *detected by public health surveillance programs* would be even greater if more-sensitive assays were used to detect transmitted HIV-1 drug resistance [58, 59] and if resistance testing were performed at the earliest opportunity (discussed below).

Conventional testing may also underestimate the full extent of HIV-1 drug resistance because, at least for persons receiving and failing ARV therapy, viral variants detected in plasma may not be representative of the viral populations in lymph nodes and PBMCs [60] or compartmentalized virus in the male [61-63] and female genital tracts [64, 65]. Although the published data are limited, transmitted HIV-1 drug resistance mutations that are detected in plasma may similarly not be representative of other viral populations because dissemination occurs immediately following HIV-1 acquisition [66, 67]. In several case reports, transmitted HIV-1 drug resistance mutations identified by consensus sequencing in PBMCs of ARV-naïve individuals were not detected in the blood plasma [27, 68]. However, some studies have shown concordance of transmitted HIV-1 drug resistance mutations in plasma and PBMCs [23, 25], while others have shown that similar numbers of individuals may have transmitted HIV-1 drug resistance mutations detected only in plasma or only in PBMCs [69, 70]. Transmitted HIV-1 drug resistance poses a significant potential threat to the successes that have been achieved to date in the treatment of HIV-1 infected individuals, and additional studies are needed using more-sensitive assays and with specimens instead of or in addition to blood plasma to increase the accuracy of estimates of the true prevalence of transmitted HIV-1 drug resistance.

**3.c Dynamics of HIV-1 drug resistance during primary HIV-1 infection and over time**

Early studies suggested that the low efficiency of HIV-1 transmission produced a bottleneck that resulted in acquisition or outgrowth of a single HIV-1 clone [71-73]. Under these circumstances, viruses detected immediately following HIV-1 acquisition would be representative of viruses found throughout the body, and low-frequency viral variants would not exist. In reality, there is increasing evidence that many individuals are infected with a mixture of viruses, and this is not restricted to one gender or mode of HIV-1 acquisition [74-77].

The ability of consensus sequencing to detect a drug resistant virus among this viral mixture depends on what proportion of viral variants has the mutation, a characteristic that is likely determined by whether the mutation impacts viral fitness relative to wild-type variants. HIV-1 drug resistance mutations that can be detected by consensus sequencing either could represent a pure population of mutant viruses or may be associated with minimal reductions in relative viral fitness. A study of *fourteen* subjects with transmitted HIV-1 drug resistance found that the *median* replication capacity (RC) of mutants detected by consensus sequencing was *72%* (range 16-208%) of wild-type [9]. In contrast, viruses with drug resistance mutations that are associated with lower relative fitness will likely be overgrown by wild-type viruses over time [78]. It is possible that overgrowth could occur immediately after HIV-1 acquisition, and some HIV-1 drug resistance mutations could only ever be detected using more-sensitive resistance assays, even during primary HIV-1 infection. In two related studies, allele-specific real-time PCR was used to detect three low-frequency HIV-1 drug resistance mutations [M184V, encoding for nucleoside reverse transcriptase inhibitor (NRTI) resistance; K103N, encoding for non-nucleoside reverse transcriptase inhibitor (NNRTI) resistance; and L90M, encoding for protease inhibitor (PI) resistance]. At least one of these three mutations was detected by this method but not by consensus sequencing in five (10%) of 49 ARV-naïve subjects with primary HIV-1 infection [58] and seven (47%) of 15 ARV-naïve subjects with established HIV-1 infection [33]. In another study of 147 ARV-naïve subjects with established HIV-1 infection, a real-time PCR assay increased the detection of D67N (NRTI resistance) by 71%, K70R (NRTI resistance) by 56%, M184V by 20%, and L90M by 25% compared to consensus sequencing [59]. These studies provide additional evidence for the transmission of viral mixtures and the potential use of more-sensitive assays to identify greater numbers of individuals with transmitted HIV-1 drug resistance.

The continued detection (sometimes called "persistence"*)* of HIV-1 drug resistance mutations over time is also likely to be associated with viral fitness. In acquired HIV-1 drug resistance, mutations that impair viral fitness are quickly overgrown by wild-type virus after the removal of ARV selection pressures [79]. In contrast, the *fourteen* subjects described in the analysis above who were infected with mutant viral variants that had *median RC of 72%* at baseline were followed longitudinally for a median of *108 (range 15-226) weeks*; the mutant variant was lost to detection by consensus sequencing in only one subject *148 weeks* following HIV-1 infection [9]. Others have also found that transmitted HIV-1 drug resistance mutations identified by consensus sequencing can be detected in PBMCs, blood and seminal plasma for years after HIV-1 infection [17-25]. However, we are unaware of published data that have systematically evaluated the detection of low-frequency HIV-1 mutations in plasma and PBMCs over time in subjects with primary HIV-1 infection. It is likely that the same factors (e.g. low fitness) that result in the presence of transmitted HIV-1 drug resistance at low levels following HIV-1 acquisition would continue to affect the relative growth of viral variants in the absence of ARV therapy. With time, levels of low-frequency viral variants could fall below the limits of detection of even the most sensitive drug resistance assays. Further understanding of the dynamics of low-frequency HIV-1 drug resistance in different compartments will help guide recommendations for the timing and selection of HIV-1 drug resistance assays to maximize the identification of transmitted HIV-1 drug resistance.

**3.d Impact of low-frequency HIV-1 drug resistance mutations on response to ARV therapy**

A combination of multiple factors, including medication adherence [80, 81], ARV drug levels [82], and regimen potency predicts the long-term success of ARV therapy [83, 84]. Conversely, detection of resistance mutations by consensus sequencing during ARV therapy is associated with the failure to maintain virologic suppression [85]. In the setting of virologic failure, the use of HIV-1 drug resistance testing to select active ARVs for a subsequent treatment regimen is associated with modest improvement in virologic response [86, 87].

In contrast, the identification of transmitted HIV-1 drug resistance does not clearly have negative clinical implications. Rapid progression of HIV-1 disease has been reported in cases of transmitted HIV-1 drug resistance [17, 88], but transmitted HIV-1 drug resistance identified by consensus sequencing does not uniformly confer a poor prognosis on the natural history of HIV-1 infection [89-91] or on response to ARVs [50, 52, 53, 89, 92-98]. In some of these studies, results of resistance testing guided the selection of fully active ARV regimens, but, in others, treatment response was not dependent on the number of active ARV agents in the treatment regimens. However, because some of these studies do show that negative consequences of transmitted HIV-1 drug resistance can occur when subjects receive ARV regimens that are not fully active, national experts recommend resistance testing for ARV-naïve HIV-infected individuals prior to initiating ARV therapy [8].

Low-frequency HIV-1 drug resistance mutations that are identified by more-sensitive assays have also been associated with poor clinical outcomes in single case reports and in *a few larger studies* [2, 3, 5, 28, 29]. *Another recent study found no association between low-frequency mutations and treatment [4].* However, because these studies may have only identified some but not all clinically relevant mutations and because the control groups likely included subjects who had low-frequency mutations in other loci, there may be significant confounding in these analyses. One of the advantages of OLA over many other more-sensitive drug resistance assays is that OLA can screen large numbers of codons with significantly less cost. Our pilot study (described in **4. Preliminary Studies)** was underpowered to evaluate whether the presence of a mutation at any one individual codon negatively impacted the response to treatment. It is likely that all low-frequency HIV-1 drug resistance mutations do not uniformly affect the response to ARV therapy, and, if low-frequency viral variants comprise only a small fraction of the viral population or do not represent viable virus, it is possible that viral suppression can be achieved when the predominantly wild-type population responds to ARV therapy.

While continued suppression of plasma virus is usually successful in persons treated with ARVs during primary HIV-1 infection [99-105], ARV therapy has only a modest effect on HIV-1 DNA levels in PBMCs [30-32, 106]. Over time, this could lead to the selection of existing mutant viral variants and, with even a small amount of ongoing viral replication, to continued viral evolution and the potential development of new HIV-1 drug resistance mutations. One study of ten subjects with primary HIV-1 infection [32] found that combination ARV therapy had no impact on PBMC HIV-1 DNA levels, and two (22%) of nine subjects who had plasma HIV-1 RNA levels below 50 copies/mL throughout the study had new mutations detected by consensus sequencing in PBMC HIV-1 DNA within six months after the start of ARV therapy. In another study, six (40%) of 15 previously ARV-naïve subjects with established HIV-1 infection had low-frequency HIV-1 drug resistance mutations detected in plasma soon after initiating ARV therapy [33]; the detection of additional mutations was associated with delayed time to virologic suppression. Neither of these studies had sufficient follow-up to assess whether the detection of additional mutations was associated with a shorter time to virologic failure, but these findings suggest the potential need for caution in prescribing ARV therapy during acute HIV-1 infection [99, 100, 104, 107] when viral replication is rampant, HIV-1 RNA levels are frequently greater than 1,000,000 copies/mL [108], and individuals may therefore be at relatively increased risk of selecting for transmitted mutants or developing new HIV-1 drug resistance mutations.

Recent data suggests that the timing of treatment in relation to prior ARV exposure may also be an important factor associated with risk of virologic failure. Receipt of single-dose nevirapine (SD-NVP) for the prevention of mother-to-child transmission was associated with poor response to NVP-based ARV therapy when the treatment regimen was initiated within six months of the SD-NVP [109]. Although this study only reported results of HIV-1 drug resistance testing identified by consensus sequencing at baseline and at virologic failure, other studies using more-sensitive assays have shown that the K103N mutation can be detected at low levels for up to five years following SD-NVP [110-112]. It is possible that the interaction between the delayed initiation of ARV therapy and reduced risk of subsequent virologic failure following SD-NVP was mediated by decay in the level of low-frequency HIV-1 drug resistance over time. In contrast to HIV-1 drug resistance that is acquired during therapy, there are no published data to suggest whether the decay of transmitted HIV-1 drug resistance over time since HIV-1 acquisition could impact the risk of virologic failure and whether deferring ARVs during primary HIV-1 infection may be beneficial for this reason. We will address this concept in Aim #2 of this proposal.

These studies suggest that our current understanding of the dynamics of HIV-1 drug resistance based on results of consensus sequencing may be somewhat superficial. Additional data are needed to determine whether there is a threshold level where transmitted HIV-1 drug resistance impacts response to ARV therapy or whether all transmitted resistance is clinically relevant. If new drug resistance mutations are detected in the PBMCs of a high proportion of subjects during ARV therapy and if this is associated with increased risk of virologic failure, resistance testing in PBMCs could be developed as a component of monitoring strategies for HIV-infected individuals receiving ARV treatment. Finally, if levels of transmitted HIV-1 drug resistance mutations decay prior to the initiation of ARV therapy and do not subsequently reappear during therapy, this would suggest a new rationale *to defer* ARV treatment during primary HIV-1 infection *given the current absence of data showing a clear benefit of acute treatment in reducing the viral set point or delaying other long-term clinical outcomes*.

**3.e "Transmission fitness" of HIV-1 drug resistance**

Overall, the transmission dynamics of HIV-1 drug resistance mutations remains poorly understood, both at the individual level (between partner-pairs) and among HIV-infected populations. At the individual level, it is still unclear whether HIV-1 drug resistance mutations reduce "transmission fitness", the relative ability of a virus to infect a susceptible host. Using an *in vitro* assay, one study found that MDR HIV-1 was, in fact, more infectious than wild-type virus [113], although the viruses that were studied did not have the decreased RC that is typically associated with multi-drug resistance [114, 115]. Greater RC has been correlated with increased risk of transmission in an experimental model of vaginal transmission of simian immunodeficiency virus [116] and in analysis of mother-to-child transmission [117]. Prediction models for population-level transmission dynamics of HIV-1 drug resistance must be complex, because there is wide variability in RC for any given mutation [118], even between the virus transmitted from source to recipient partners [39], *and host factors likely play a significant role in the clinical progression of disease and risks for subsequent HIV-1 transmission [119]*.

It is possible that the presence of HIV-1 drug resistance is not independently associated with the risk of HIV-1 transmission, and the likelihood of transmission is proportional to the quantity of virus in the genital tract [120-123]. Given that HIV-1 RNA levels in seminal plasma are highest in the first few months following HIV-1 acquisition [124, 125] and sexual transmission from source partners with primary HIV-1 infection contributes to some significant proportion of overall HIV-1 incidence [126-130], it is possible that individuals with primary HIV-1 infection may contribute disproportionately to the transmission of HIV-1 drug resistance. Transmission of drug resistance from ARV-naïve sources has been previously documented following mother-to-child [131], heterosexual [132], and same-sex [25, 113] transmission; clusters of newly-infected subjects with transmitted HIV-1 drug resistance mutations have also been identified [133-135]. In contrast, even if individuals with established infection who are receiving ARV therapy have HIV-1 drug resistance in genital fluids, they are also likely to have lower levels of HIV-1 in the male [124] and female [136] genital tracts and, consequently, a lower risk of HIV-1 transmission [121].

The low transmission fitness of HIV-1 drug resistance has been suggested by population-level analyses that have found that HIV-1 drug resistance mutations occur in smaller proportions of newly-infected individuals compared to hypothetical cohorts of potential transmitters [35-38]. All but one of these models fails to account for transmission that occurs from source partners who also have primary HIV-1 infection. In this one model, when the authors increased the estimated proportion of transmission that occur from persons with primary HIV-1 infection from 30% to 50%, HIV-1 drug resistant viruses were no less likely to be transmitted than wild-type viruses [38]. In contrast, empiric studies of transmission pairs have reported high concordance of HIV-1 drug resistance mutations in source and recipient partners when tested by consensus sequencing [19, 21, 25, 39, 40]. These studies indicate that it may not be possible to infer individual-level estimates of the risk of transmission HIV-1 drug resistance from simple population-based models, and additional research with confirmed partner-pairs is needed to identify the factors associated with transmission of HIV-1 drug resistance between individuals.

Empiric measurements of individual-level estimates are necessary to develop more accurate models of the population-level transmission dynamics of HIV-1 drug resistance. These models will be essential to inform the design of public health interventions to reduce the transmission of HIV-1 drug resistance by quantifying the relative contributions of HIV-1 transmission from ARV-naïve and ARV-experienced individuals. If secondary transmission occurs in large part from ARV-naïve individuals with primary HIV-1 infection, recommendations that attempt to reduce transmitted HIV-1 drug resistance by targeting only ARV-experienced individuals (e.g. by increasing medication adherence or discontinuing non-suppressive ARV regimens [137, 138]) may not have the anticipated impact. In contrast, the growing number of public health pooled HIV nucleic acid amplification testing programs designed to identify highly infectious individuals with acute HIV-1 infection [139-142] may have a heretofore unrecognized impact on reducing the prevalence of transmitted HIV-1 drug resistance.

**3.f Oligonucleotide ligation assay (OLA)**

The range of studies proposed in this application have not previously been conducted using drug resistance assays that were able to detect viral variants comprising less than 10-50% of the viral population [11-16]. Examples of these more-sensitive assays include single genome sequencing [34, 143], primer-specific PCR [16], a ligation-amplification assay [144], and parallel allele-specific sequencing [16]. OLA was developed by Dr. Lisa Frenkel and has several advantages over the other more-sensitive assays (**Table 0**).

**Table 0: Comparison of the sensitivity and other characteristics of HIV-1 drug resistance assays**

|  | Sensitivity | Specificity | Tolerant to polymorphisms? | Cost | Technology required |
| --- | --- | --- | --- | --- | --- |
| Consensus Sequencing [11-16] | 10-50% | high | yes | $$$ | moderate |
| OLA [145-147] | 2-10% | high | yes | $$ | moderate |
| Single genome sequencing [143] | 2-10% | high | yes | $$$$$ | moderate-plus |
| Allele-specific PCR [143, 148] | 0.04 to 0.8% | low | no | $$ | moderate |
| Parallel allele-specific sequencing (PASS) [149] | 0.01 to 0.1% | high | yes | $$$$$ | complex, investigational |

OLA can reliably detect HIV-1 with reverse transcriptase mutations that occur in as little as 2-5% of the viral mixture ([145] and Frenkel, personal communication) and protease mutations that occur in as little as 5-10% of the population [146, 147]. OLA tolerates genetic polymorphisms near the sites of target mutations except for the two nucleotides to either side of the ligation site [150]. This is an important attribute of OLA given that polymorphisms and mutations commonly occur in regions of the genome that encode for HIV-1 drug-resistance. OLA is semi-quantitative and can be used to estimate the frequency or level of viral variants with HIV-1 drug resistance by comparing results of patient samples against a standard curve [146]. Most importantly, compared to single genome sequencing by cloning or end-point dilution methods, the OLA is one of the most rapid and economical methods that can be used to screen for low-frequency HIV-1 drug resistance mutations at multiple loci.

**Table 1: Mutations tested by OLA and associated**

**level of ARV resistance for subtype B viruses^1^**

|  | Level of resistance | |
| --- | --- | --- |
|  | High or intermediate | Low |
| **Reverse Transcriptase** | | |
| M41L |  | AZT, d4T |
| K65R | 3TC, *ABC,* ddI, FTC, TDF | d4T |
| K70R |  | AZT |
| L74V | ABC, ddI |  |
| M184V | 3TC, FTC |  |
| T215F | AZT, d4T | ABC, ddI |
| T215Y | AZT, d4T | ABC, ddI, TDF |
|  | | |
| K103N | EFV, NVP |  |
| Y181C | NVP, ETV | EFV |
| G190A | EFV, NVP | ETV |
|  | | |
| **Protease** | | |
| D30N | NFV |  |
| I50V | FPV | DRV, LPV |
| V82A | IDV, NFV | ATV, LPV |
| V82S | IDV, NFV | ATV, LPV, TPV |
| V82T | IDV, NFV, *TPV* | ATV, LPV |
| I84V | FPV, NFV, SQV | ATV, IDV, TPV |
| N88D |  | NFV |
| L90M | NFV, SQV | ATV, FPV, IDV |

from http://hivdb.stanford.edu/, accessed 8/13/08

^1^Probes for V106M (which confers resistance to NNRTIs) are available for use with subtype C viruses.

AZT: zidovudine, d4T: stavudine, 3TC: lamivudine, ddI: didanosine, FTC: emtricitabine, TDF: tenofovir, ABC: abacavir; EFV: efavirenz, NVP: nevirapine, ETV: etravirine, NFV: nelfinavir, FPV: fos-APV, DRV: darunavir, LPV: lopinavir, IDV: indinavir, NFV: nelfinavir, ATV: atazanavir, TPV: tipranavir, SQV: saquinavir

The mutations and associated controls for which oligonucleotide probes have been developed are shown in **Table 1** [151]. Increasing numbers of projects have used OLA to determine the presence of low-frequency HIV-1 drug resistance because of the reasons described above. Many of these projects have tailored OLA for use in developing countries to study non-subtype B viruses [152-154], HIV-2 [155], and resistance following prevention of mother-to-child transmission [156-158].

**3.g Summary**

Although results from many previous studies have increased our understanding of the prevalence and impact of transmitted HIV-1 drug resistance among HIV-infected individuals, most of these studies have been limited by their use of consensus sequencing, and many questions relevant to clinical and public health remain unanswered. Public health surveillance programs would benefit from more accurate estimates of the prevalence of transmitted HIV-1 drug resistance, as would clinicians who recommend empiric ARV therapy without the benefit of resistance testing results for individuals with acute HIV-1 infection or in cases of post-exposure prophylaxis. Studies are needed to understand whether there are differences in the time to loss of detection of HIV-1 drug resistance mutations that are detected by consensus sequencing and by more-sensitive methods in order to guide recommendations for the timing of HIV-1 drug resistance testing. If transmitted low-frequency HIV-1 drug resistance mutations are rapidly lost to detection following HIV-1 acquisition, consideration should be made as to whether resistance testing should be routinely incorporated into HIV-1 confirmatory testing and public health surveillance (*i.e. prior to entry into clinical care*), especially in areas where recent infections *and public health-based HIV testing account for* a large proportion of new HIV-1 diagnoses. Studies are needed to assess the impact of low-frequency HIV-1 drug resistance mutations on disease progression and response to ARV therapy to determine whether the routine use of more-sensitive resistance assays should be considered for future study and widespread use. Further understanding of the factors associated with the dynamics of HIV-1 drug resistance during ARV therapy is needed to help clinicians to make decisions about the timing and potency of ARV treatment regimens. Finally, "transmission fitness" is a concept that is poorly understood, and additional data is needed to determine whether transmitted HIV-1 drug resistance impacts the risk of HIV-1 transmission at both the individual and population level. The studies described in this application have been specifically designed to answer these and other important questions.

**4. Preliminary Studies**

**4.a Seattle Primary Infection Program**

The Seattle Primary Infection Program (SeaPIP) is a highly productive, interdisciplinary collaboration of nationally-recognized HIV-1 investigators who have published widely on the clinical, immunologic and virologic consequences of primary HIV-1 infection [34, 74, 99, 107, 108, 124, 159-180]. Our recent research has provided insight into host-pathogen relationships, the impact of immune escape on viral fitness, HIV-1 dynamics in different viral reservoirs and compartments, therapeutic interventions, HIV-1 drug resistance, and the formulation of HIV-1 vaccine strategies. The strength of SeaPIP has been its focus on the performance of in-depth, multifaceted, longitudinal analyses of subjects with acute HIV-1 infection and their source partners. A letter of support from the SeaPIP Principal Investigator (Dr. James Mullins, PhD) is included with this proposal.

## Table 2: Demographic characteristics of all PIC enrollees and King County HIV/AIDS patients

| Characteristics, # (%) | *PIC*  *N=319* | King Co.  N=6268 |
| --- | --- | --- |
|  | *n (%)* | % |
| Male | *310 (97%)* | 90% |
| Caucasian | *282 (88%)* | 68% |
| African/African-American | *11 (3%)* | 16% |
| Hispanic | *23 (7%)* | 9% |
| Nat. Amer./Pac.Is./Asian | *9 (3%)* | 5% |
| Other/Mixed | *17 (5%)* | 2% |
| MSM | *285 (89%)* | 69% |
| MSM/IDU | *15 (5%)* | 8% |
| IDU | *5 (2%)* | 5% |
| Heterosexual | *14 (4%)* | 10% |
| *Fiebig^1^ pre-stage I* | *1 (0.3%)* | *NA* |
| *stage I* | *13 (4%)* | *NA* |
| *stage I/II^2^* | *74 (23%)* | *NA* |
| *stage II* | *6 (2%)* | *NA* |
| *stage III* | *1 (0.3%)* | *NA* |
| *stage IV* | *37 (12%)* | *NA* |
| *stage V* | *56 (18%)* | *NA* |
| *stage V/VI^2^* | *113 (35%)* | *NA* |
| *stage VI* | *6 (2%)* | *NA* |

###### NA: Not Available, MSM: men who have sex with men, IDU: injection drug user

^1^stage used to determine study eligibility

^2^stages could not be further resolved due to lack of available data or specimens for retesting

**4.b University of Washington Primary Infection Clinic (PIC)**

Since 1992, *319* subjects with HIV-1 infection have been enrolled and followed for a median of *42 (range 0-184)* months in an observational cohort at the PIC. **Table 2** shows the demographic characteristics of subjects with primary HIV-1 infection enrolled in the PIC cohort compared to persons with predominantly established HIV-1 infection reported to Public Health - Seattle & King County [181]**.** *Seventy-eight subjects* were enrolled within 30 days of their estimated dates of HIV-1 infection. *Women and African/African-American subjects are under-represented relative to the number of individuals diagnosed with HIV-1 infection in King County because the majority of individuals comprising these categories are African immigrants who did not acquire their infection locally. Additional details regarding the inclusion of women and minorities are provided in* ***8. Protection of Human Subjects.***

ARV treatment was started in some subjects through non-randomized research protocols or through primary care providers. Despite the existence of national guidelines that have recommended the consideration of therapy during acute HIV-1 infection, the proportion of PIC subjects starting therapy immediately following HIV-1 acquisition has decreased over time, likely due to an increasing number of studies that have found no direct evidence of sustained clinical benefits in subjects treated during primary HIV-1 infection*. Forty-eight (67%) of 72 subjects enrolled in 1999-2001, 40 (68%) of 59 subjects enrolled in 2002-2004, and only 22 (39%) of 57 subjects enrolled in 2005-mid 2008 chose to receive ARV therapy in the first year after HIV-1 acquisition.* Plasma and PBMCs are stored and available for each of these subjects throughout study follow-up, and records of CD4^+^ T-cell counts, HIV-1 RNA levels, and clinical endpoints are available at regular intervals.

**4.c Identification and recruitment of persons with acute and very early HIV-1 infection**

The projects proposed in this application will study subjects with acute and very early HIV-1 infection (Fiebig stages I to IV) and their source partners. Referrals to the PIC for possible study enrollment come from diverse sources, including the clinic website (http://depts.washington.edu/hpic), private health care providers, emergency departments, urgent care clinics, other research cohorts, and a pooled HIV-1 RNA testing program through Public Health - Seattle & King County (PHSKC). Since September 2003, *Dr. Stekler has been the lead author on several articles originating from the PHSKC HIV testing program that* has identified *37* *MSM* who were HIV-1 antibody-negative and RNA-positive [139, 173, 182] and *17 additional* HIV-1 antibody-positive persons who had false-negative rapid HIV-1 antibody test results during early HIV-1 infection [183]. Nearly all of these persons were urgently referred to the PIC for education about primary HIV-1 infection and discussion of study enrollment at the PIC. These data demonstrate the continued ability of the PIC to recruit subjects with acute HIV-1 infection, the collaboration *that Dr. Stekler has nurtured* between the PIC and PHSKC *as her public health role has expanded*, and the clinical resource that the PIC provides for PHSKC. A letter of support from Dr. Robert Wood, MD, Director of the PHSKC HIV/AIDS Program, is included with this application.

**4.d Clinical benefits of initiating ARVs during primary HIV-1 infection**

An early analysis of the PIC that compared 20 subjects treated with a PI-containing regimen suggested that use of ARV therapy in primary HIV-1 infection was associated with a delay in the development of HIV-associated conditions compared to untreated historical controls [99]. An updated analysis (n=250) of the cohort evaluated the impact of initiating ARV therapy during acute and early HIV-1 infection and had a different conclusion [184]. For the retrospective analysis, treated subjects were divided *a priori* into groups that began ARV therapy within 30 days after HIV-1 infection (acute treatment, n=41), 31-180 days after HIV-1 infection (early treatment, n=82), or greater than 180 days after HIV-1 infection (delayed treatment, n=35). Because of differences at baseline, the untreated control subjects were divided into historical (n= 29) and contemporary (n=63) control groups based on enrollment into the PIC before or after 1996. We compared the time to the development of an HIV-associated diagnosis [99]. Cox proportional hazard models were adjusted for age, gender, race/ethnicity, acute retroviral symptom severity, baseline CD4^+^ T-cell count and HIV-1 RNA level.

The median time to the first HIV-related diagnosis in these groups was 3.8 [interquartile range (IQR) 2.9-7.8] years with acute treatment, 5.6 (IQR 3.9-6.6) years for early treatment, 7.3 (IQR 4.4-10.2) years for treatment delayed for more than 180 days, 4.6 (IQR 3.4-6.7) years for contemporary controls, and 1.8 (IQR 1.1-2.9) years for historical controls (**Figure 1**). Treatment during acute infection was not associated with a delay in the time to an HIV-associated diagnosis compared to treatment initiated later during primary HIV-1 infection.

**Figure 1: Time to HIV-related diagnoses by treatment group over time since HIV-1 infection (1a) and start of ARVs (1b)**


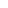


25

50

75

100

0

1000

2000

3000

4000

5000

Days following HIV infection


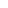


group 1 (<30 days)

group 2 (31-180 days)

group 3 (>180 days)

historical controls

contemporary controls

Figure 1a

25

50

75

100

0

1000

2000

3000

4000

Days following start of ARV therapy

group 1 (<30 days)

group 2 (31-180 days)

group 3 (>180 days)

Figure 1b

**Figure 1a**: Compared to contemporary controls, adjusted hazard ratios for the development of HIV-1 associated diagnoses were 0.58 (95% CI 0.26-1.3, p=.2) for the acute treatment group, 0.56 (0.30-1.0, p=.07) for the early treatment group, and 0.27 (0.13-0.57, p<.001) for the delayed treatment group. Time to HIV-1 associated diagnosis was shorter in historical versus contemporary control subjects (p=.002). **Figure 1b** shows results of the analysis when adjusted for time since the start of ARV therapy.

Results from this study do not support the routine use of ARV therapy during acute or early HIV-1 infection, underscore issues of confounding and the importance of the choice of control groups in observational studies, and suggest that data from randomized clinical trials are still needed to address definitively whether treatment during acute HIV-1 infection has clinical benefit. These data also demonstrate our ability to perform the complex analyses needed to evaluate clinical outcomes in the studies described in this proposal and our ability to understand the context of any risks and benefits of treatment during primary HIV-1 infection that we may identify.

**4.e HIV-1 dynamics in seminal plasma from men with primary HIV-1 infection**

In order to understand the risk of sexual HIV-1 transmission over time, we compared blood and seminal HIV-1 dynamics in men with primary HIV-1 infection. Between 1993 and 2005, 110 men in the PIC cohort provided 327 semen specimens [124] (see **Appendix**). The initial blood and seminal plasma HIV-1 RNA levels in untreated men were only moderately correlated (*Spearman r=0.38, p=.0002*). The estimated peak and set point HIV-1 RNA levels were lower in seminal plasma than blood plasma by *0.8* (p=.001) and *0.7* (p<.001) log_10_ copies/mL, respectively, and HIV-1 RNA decay rates were similar in the two compartments (p=.4).

Another paper described seminal HIV-1 dynamics of subtype C virus in men seeking attention for sexually transmitted infections in Malawi [125]. Further studies with more intensive sampling are still required in order to further evaluate HIV-1 dynamics in seminal plasma, including the timing and level of peak HIV-1 RNA levels in seminal plasma following HIV-1 acquisition, the timing of HIV-1 set point in the male genital tract, levels of HIV-1 drug resistance that can be detected over time, and the impact of ARV therapy with variable penetration into the genital tract on HIV-1 RNA levels and drug resistance in seminal plasma. Studies to address these questions are currently being proposed in this and another separate application. These data demonstrate our ability to obtain genital secretions and quantify HIV-1 RNA levels in semen. A letter of support from Dr. Robert Coombs, MD PhD, Director of the University of Washington Clinical Retrovirology Laboratory, describes our continued collaboration and is included with this application.

**4.f The oligonucleotide ligation assay (OLA)**

**Figure 2: OLA in mixtures of wild-type and L90M**

**Figure 2:** DNA from prepared mixtures of wild-type and drug resistant genomes was PCR amplified and evaluated by OLA. The mean absorbance readings (+/- SD) are shown for wild-type (white bars, OD_450_) and mutant (shaded bars, OD_490_) genomes.

Over the last decade, the Frenkel laboratory has developed extensive expertise in evaluation of HIV-1 genotypic resistance, including the development of OLA as a sensitive and high throughput method to detect mutations associated with HIV-1 drug resistance. The sensitivity of OLA was determined by using serial dilutions of mutant virus in wild-type mixtures [146]; one example is shown in **Figure 2.** Mutant genomes for all protease mutations were detected consistently by OLA in concentrations as low as 5%. These data also show that OLA can be used to generate semi-quantitative results evaluating the proportion ("level") of viral variants with HIV-1 drug resistance.

This same manuscript presented results of a study that used OLA and consensus sequencing to evaluate 312 codons from 54 subjects with known protease sequences (**Table 3**) [146]. OLA failed to identify three mutant codons that were identified as wild-type (n=2) or indeterminate (n=1) by consensus sequencing. Consensus sequencing failed to identify seven mutant codons in mixtures of wild-type and mutant virus.

**Table 3: OLA and consensus sequencing in identification of previously detectable** protease mutations

|  | | Consensus sequencing | | | Total |
| --- | --- | --- | --- | --- | --- |
|  |  | mutant | mixture | wild-type |  |
| OLA | mutant | 80 | | 0 | 57 |
|  | mixture |  |  | **7** | 30 |
|  | wild-type | 0 | **2** | 212 | 214 |
|  | indeterminate | **1** | 0 | 10 | 11 |
|  | Total | 68 | 15 | 229 | 312 |

Another study investigated whether acquired low-level HIV-1 drug resistance could be detected in PBMCs after mutations were lost to detection in plasma. Sixty subjects were selected for study if they previously had evidence of HIV-1 drug resistance by consensus sequencing and subsequently had those mutations recede below the level of detection [147]. These mutations were in codons conferring resistance to NRTIs (n=58 codons), NNRTIs (n=18 codons), and PIs (n=15 codons). Samples were tested a mean of 13.7 (range 3.7-32) months following the specimen in which the mutant had last been detected. Fifty-four percent of mutant codons were detected by OLA in PBMCs, 28% of mutant codons were detected by OLA in plasma, and 23% of mutant codons were detected by consensus sequencing in PBMCs. These data demonstrate our ability to compare results of OLA and consensus sequencing in PBMCs and plasma and indicate that acquired HIV-1 drug resistance mutations can be detected in PBMCs when they are lost to detection in plasma.

The semi-quantitative nature of OLA has been used to describe the transmission, selection, and levels of resistance mutations over time in infants who became HIV-infected despite receiving SD-NVP to prevent mother-to-child transmission in Mozambique [157]. HIV-1 drug resistance was evaluated for 46 HIV-1 infected infants using dried blood spots that had been collected at 0, 2, 4, 6, and 8 weeks of age. Relative concentrations of drug resistant mutants were determined using a standard curve with 0%, 2%, 5%, 10%, 25%, 50% and 100% mutants. We hypothesized that, if infants acquired HIV-1 around the time of delivery, receipt of SD-NVP during peri-partum and immediate post-partum infection would result in the transmission or selection of mutant viruses that would populate long-lived viral reservoirs and persist at stable levels over time. In contrast, we hypothesized that, if infants were HIV-infected *in utero*, receipt of SD-NVP after HIV-1 infection had been established would also select for mutants but that the level of detection of the mutant population would decrease after ARV selection pressure was removed. Consistent with our hypotheses, only two infants who acquired HIV-1 infection around the time of birth had mixtures of mutant and wild-type virus; the remaining infants had 100% wild-type virus (n=9) or 100% mutant virus (n=7). Decay in levels of mutant viruses was not observed in these infants. In contrast, all infants who were infected *in utero* (n=22) had wild-type HIV-1 at birth, and 20 (91%) of these infants had mutant virus subsequently detected. Decay was observed in some of these infants (**Figure 3**). These data demonstrate our ability use the semi-quantitative nature of OLA to compare levels of drug resistance mutants over time.

**Figure 3: Changes in levels of K103N following *in utero* HIV-1 infection and post-partum use of NVP Figure 3:** The selection and decay of K103N is shown for an infant who was infected *in* utero and received NVP after birth. At birth (0 wks), only wild-type virus was detected. Levels of K103N subsequently increased until 4 weeks of life and then decreased until the mutation was no longer detected at 8 weeks of life.

**4.g Transmitted HIV-1 drug resistance among subjects enrolled in the PIC Cohort study**

A New Investigator Award from the UW Center for AIDS and STD to Dr. Stekler provided supplemental funding to SeaPIP for a pilot project to evaluate the prevalence and impact of transmitted low-frequency HIV-1 drug resistance in subjects with primary HIV-1 infection. Consensus sequencing and OLA were performed on the first available plasma and PBMC specimens from ARV-naïve subjects enrolled in the UW PIC cohort between 1996 and 2005. Subjects were preferentially selected for study if they had enrolled in the cohort within 30 days of HIV-1 infection or if they initiated ARV therapy during follow-up. Demographic and other characteristics of subjects were otherwise similar to the entire PIC cohort.

*Baseline resistance test results are available from 100 subjects tested to date. Consensus sequencing and OLA were performed using stored frozen plasma and PBMCs obtained a median of 29 (IQR 19-66) and 30 (IQR 19-66) days after HIV-1 infection, respectively. Consensus sequencing detected transmitted HIV-1 drug resistance in 6 (6%) of 100 subjects, and OLA detected low-frequency mutations in 28 (30%) of the 94 subjects who had no mutations detected by consensus sequencing. OLA identified significantly more subjects with transmitted resistance in both plasma and PBMCs compared to consensus sequencing (****Table 4****).*

**Table 4: Number of ARV-naïve subjects with transmitted** HIV-1 drug resistance at baseline

| **4a:** OLA v consensus sequencing in plasma | | | |
| --- | --- | --- | --- |
|  | | OLA | |
|  |  | - | + |
| sequencing | - | 74 | **21** |
|  | + | **0** | 5 |

| **4b**: OLA v consensus sequencing in PBMCs | | | |
| --- | --- | --- | --- |
|  | | OLA | |
|  |  | - | + |
| sequencing | - | 78 | **16** |
|  | + | **2** | 4 |

| **4c**: consensus sequencing in plasma v PBMCs | | | |
| --- | --- | --- | --- |
|  | | PBMCs | |
|  |  | - | + |
| plasma | - | 94 | **1** |
|  | + | **0** | 5 |

| **4d:** OLA in plasma v PBMCs | | | |
| --- | --- | --- | --- |
|  | | PBMCs | |
|  |  | - | + |
| plasma | - | 66 | **8** |
|  | + | **14** | 12 |

+ = subjects w/ ≥1 mutation or mixture.

- = subjects w/o mutations or w/ indeterminate results

Compared to consensus sequencing, OLA identified more subjects with transmitted resistance in both plasma (**4a**, p<.0001, by McNemar's exact test) and PBMCs (**4b**, p=.001). There was not a significant difference in the number of subjects with mutations detected by OLA in plasma and PBMCs (**4d,** p=.3).

***Table 5*** *shows the specific mutations that were detected. No subject had the L74V (NRTI) or D30N (PI) mutations. Four subjects had one HIV-1 drug resistance mutation detected by both consensus sequencing and OLA (one with M184V, two with G190A, and one with L90M). The subject with L90M and two other subjects had intermediate genotypes between wild-type and mutant (T215Q and T215D) that were detected by consensus sequencing; screening for T215F and T215Y by OLA yielded indeterminate results. With the use of OLA, the detection of NRTI resistance increased from 4% to 17% of subjects, the detection of NNRTI resistance increased from 2% to 9% of subjects, the detection of PI resistance increased from 1% to 19% of subjects, and the detection of multi-drug resistant HIV-1 increased from 1% to 9% of subjects. Overall, 34% (95% CI 25-44%) of subjects had transmitted HIV-1 drug resistance. These data show our ability to use OLA to identify transmitted low-frequency HIV-1 drug resistance and demonstrate a high prevalence of drug resistance that was not detected by consensus sequencing in our cohort of subjects with primary HIV-1 infection.*

Table 5: Number of subjects with HIV-1 drug resistance mutations detected by OLA in PBMCs, plasma, or both, by mutation

|  | *NRTI* | | | | *NNRTI* | | | *PI* | | | | |
| --- | --- | --- | --- | --- | --- | --- | --- | --- | --- | --- | --- | --- |
|  | *K65R* | *K70R* | *M184V* | *T215F/Y* | *K103N* | *Y181C* | *G190A* | *I50V* | *V82A/S/T* | *I84V* | *N88D* | *L90M* |
| *PBMCs only* | *4* | *2* | *5* | *5* | *2* | *-* | *1* | *1* | *1* | *-* | *4* | *-* |
| *Plasma only* | *2* | *1* | *2* | *3* | *-* | *4* | *-* | *7* | *-* | *2* | *3* | *-* |
| *Both* | *2* | *-* | *-* | *-* | *-* | *-* | *2* | *-* | *1* | *2* | *-* | *1* |

*Eighty-three (88%) of the 94 subjects initiated ARV therapy a median of 45 (IQR 24-107) days after HIV-1 infection. Four (5%) subjects who initiated ARV therapy had HIV-1 drug resistance mutations identified by consensus sequencing, 24 (29%) subjects had low-frequency mutations identified only by OLA, and 55 (66%) subjects had no mutations that were detected by either assay. At the start of ARV therapy, the mean CD4^+^ T-cell count was 451 (SD 197) cells/mm^3^ in subjects with low-frequency mutations and 479 (SD 168) cells/mm^3^ in subjects with no detectable mutations (p=.5). The mean HIV-1 RNA level at the start of treatment was 5.0 (SD 0.8) log_10_ copies/mL in subjects with low-frequency mutations and 4.8 (SD 1.0)* *log_10_ copies/mL in subjects with no detectable mutations (p=.4).*

*Survival analyses evaluated the time to virologic suppression (HIV-1 RNA less than 50 copies/mL) among treated PIC subjects. The HIV-1 drug resistance mutations that were detected in either plasma or PBMCs from each subject were evaluated with the Stanford University HIV Drug Resistance Database [151] in order to estimate the number of active agents in a subject's treatment regimen. An ARV agent was considered to be inactive if the mutation conferred high or intermediate-level resistance to that ARV. Cox proportional hazard models compared subjects with low-frequency HIV-1 drug resistance mutations receiving fewer than three active ARV agents ("inadequate" regimens) to subjects with low-frequency HIV-1 drug resistance mutations receiving at least three active ARV agents and to subjects without detectable mutations. Analyses were adjusted for HIV-1 RNA level at the time of initiation of ARV therapy.* ***Figure 4*** *shows the Kaplan-Meier curve indicating time to virologic suppression. After adjustment for the HIV-1 RNA level at the start of ARV therapy, the hazard ratio for time to virologic suppression was 1.1 (95% CI 0.54-2.3, p=.8) for 14 subjects with low-frequency HIV-1 drug resistance mutations who received fewer than three active ARV agents and 2.3 (95% CI 1.1-4.6, p=.02) for ten subjects with low-frequency mutations treated with at least three active agents, compared to 55 subjects without detectable mutations. (Note: a hazard ratio less than 1 indicates a longer time to virologic suppression, and a ratio greater than 1 indicates a shorter time to virologic suppression.).*

**Figure 4**: Time to virologic suppression among subjects with and without low-frequency HIV-1 drug resistance

Figure 4: *The median time to virologic suppression was 109 (IQR 63-145) days for 55 subjects with no detectable HIV-1 drug resistance mutations, 105 (IQR 57-163) days for 14 subjects with low-frequency mutations that resulted in regimens with <3 active ARV agents, and 82 (IQR 57-105) days for 10 subjects who had low-frequency mutations but initiated ARV regimens containing at least three active ARVs (p=.1).*

*Only four subjects in the pilot study experienced virologic failure during follow-up. The first subject had the G190A mutation at baseline that was identified retrospectively by consensus sequencing and OLA; he initially started mega-HAART (3TC, ABC, RTV, APV, and EFV) but switched to 3TC, ABC, and NVP due to side effects and subsequently developed virologic failure. The second subject with virologic failure had the T215Y and I50V mutations detected by OLA at baseline and received a triple-nucleoside (AZT, 3TC, ABC) regimen that is no longer considered first-line therapy because of the increased risk of virologic failure compared to potent ARV regimens [185]. Two other subjects experienced virologic failure after one and eight years on a three class ARV regimen; neither had drug resistance mutations identified at baseline by either consensus sequencing or OLA. These preliminary data demonstrate our ability to evaluate the impact of transmitted low-frequency HIV-1 drug resistance on response to ARV therapy.*

**4.h The PIC Partners study and HIV-1 drug resistance in transmission pairs**

In 1997, Liu et al. [159] reported a partner-pair enrolled in the PIC Cohort study who concurrently acquired HIV-1 infection from a common source partner and subsequently experienced widely divergent courses of HIV-1 disease progression. Following identification of these subjects, SeaPIP investigators developed a greater interest in studying HIV-1 transmission pairs to understand host-virus interactions and the relationships between viral diversity, viral pathogenesis, and immune system control. An early analysis of the study described the results of partner referral for 38 subjects enrolled in the PIC cohort [186]. Eighteen (47%) of these subjects had potential source partners who were identifiable. In two instances, both members of the partner-pair developed primary HIV-1 infection and were counted in this total of 18 until the direction of transmission could be determined. Four partners were not evaluated. One partner was repeatedly HIV-negative. In ten (91%) of the remaining 11 partnerships, the transmission relationship was confirmed; these confirmed transmission pairs represent 26% of PIC enrollees during this study period.

The PIC Partners Study remains an active collaboration funded by SeaPIP (NIH P01 AI-57005: Immunology and Virology of Acute HIV Infection) and a Mentored Career Development Award to Dr. Stekler (NIH K23 AI-65243: Primary HIV-1 Infection: The Public Health Perspective) [159, 173, 186, 187]. To date, *36* potential source partners have been enrolled in either the PIC Cohort or PIC Partners studies; *32* of these partners were enrolled after 1996. *Nineteen* potential source partners have been confirmed as the source of HIV-1 infection in the PIC enrollee. **Table 6** shows demographic and other characteristics of confirmed source partners. Confirmatory studies on the remaining partner-pairs are pending.

**Table 6: Characteristics of 16 confirmed source partners at time of HIV-1 transmission**

|  | Number (range) |
| --- | --- |
| Male | 94% |
| Median age | 30 (22-35) |
| Caucasian | 88% |
| Mean plasma HIV-1 RNA (copies/ml) | 21250  (<50 - 623,532) |
| Median CD4 (cells/mm^3^) | 598 (89-1195) |
| Median duration of HIV-1 infection | 3 months  (0–10 years) |
| On ARV therapy | 1 (6%) |

**Table 7:** HIV-1 drug resistance mutations in partner-pairs tested by OLA, to date

| Pair | Source | Recipient | Confirmed? |
| --- | --- | --- | --- |
| *#1* | *Pending* | *No mutations* | *Pending* |
| *#2* | *Pending* | *T215Y* | *Yes* |
| *#3* | *Pending* | *No mutations* | *Yes* |
| *#4* | *Pending* | *No mutations* | *Pending* |
| *#5* | *Pending* | *No mutations* | *Pending* |
| #6 | Pending | I84V | Pending |
| *#7* | *Pending* | *No mutations* | *Yes* |
| #8 | K65R, T215Y, K103N | K65R, T215Y | Yes^1^ |
| #9 | No mutations | No mutations | Yes |
| #10 | Pending | No mutations | Pending |
| #11 | Pending | T215Y | Yes |
| #12 | Pending | No mutations | Yes |
| *#13* | *Pending* | *No mutations^2^* | *Yes* |
| *#14* | *K103N, M184V^2^* | *K103N^2^* | *Yes* |
| *#15* | *No mutations^3^* | *Pending* | *Pending* |

^1^Confirmed, but the direction of transmission is not clear.

*^2^Results of consensus sequencing only*.

*^3^The sample from the putative source partner in #15 was obtained during primary HIV-1 infection, prior to the initiation of ARVs and approximately four years prior to the possible transmission event. Evaluation of a specimen proximal to the transmission event will be evaluated if this relationship is confirmed.*

*Only* *two* of the recipients *in* *16* partner-pairs tested to *date* *have* had evidence of HIV-1 resistance by consensus sequencing. *In another partner-pair recently enrolled, the source partner had the K103N and M184V mutations detected by consensus sequencing at the time of enrollment in the PIC Cohort study; he transmitted HIV-1 to his partner a few months later, who was found by consensus sequencing to have only the K103N mutation*. **Table 7** shows the *resistance testing* results in the *fifteen* partner-pairs who have had at least partial testing of the transmission pair completed to date. Blood is available from all source partners, and semen is available from a total of *12* source partners. These data demonstrate our ability to identify and enroll the source partners of subjects in the PIC cohort, collect the specimens required for the study proposed in Aim #3 of this application, use OLA to characterize the HIV-1 drug resistance patterns in our partner-pairs, and detect mutations in recipient partners that were not detected by consensus sequencing.

**5. Research Design and Methods**

**5.a Overview of study design and rationale**

The studies proposed in this section will provide a better understanding of the prevalence and dynamics of low-frequency HIV-1 drug resistance mutations in ARV-naïve subjects during primary HIV-1 infection (**Aim #1**), the impact of low-frequency drug resistance mutations on response to ARV therapy (**Aim #2**), and the relative "transmission fitness" of mutant viruses (**Aim #3**). The project will be powered to perform the longitudinal analyses described in the first two studies with use of samples from *approximately 100* subjects who previously enrolled in the PIC Cohort study within 30 days after HIV-1 infection and from *80* subjects who will be enrolled prospectively. Eligibility will be restricted to subjects who acquired HIV-1 infection after 1996, when highly active ARV therapy became widely available in Seattle. OLA will be used to evaluate HIV-1 drug resistance in the PBMCs, blood and seminal plasma from ARV-naïve PIC subjects at baseline (the first available specimen), and 6 and 12 months after HIV infection (**Aim #1)** and in PBMCs 6 and 12 months after the start of ARV therapy (**Aim #2)** to detect changes in the levels of low-frequency HIV-1 drug resistance over time.

The third aim will study PIC subjects and their source partners enrolled in the PIC Cohort and PIC Partners studies. After the transmission relationships are confirmed by molecular methods, this cross-sectional analysis will compare the results of HIV-1 drug resistance testing performed on PBMCs and blood plasma from PIC subjects with the results of resistance testing performed on PBMCs, blood plasma, and genital secretions from their confirmed source partners. The partner-pairs studied in this third aim will also include retrospective analysis of previously collected specimens and continued prospective enrollment in order to maximize the precision of estimates of "transmission fitness" to be described in **5.c.3 Aim #3**.

The studies proposed in this application require access to a cohort of subjects with acute HIV-1 infection to identify transmitted low-frequency HIV-1 drug resistance that may quickly fall below the limits of detection after HIV-1 acquisition and to study transmission pairs as proximal as possible to the transmission event. The Seattle Primary Infection Program and the UW Primary Infection Clinic are ideally suited to perform these studies. Established in 1992, the PIC has enrolled and followed over 300 subjects with acute and early HIV-1 infection, and our ongoing PIC Partners study was specifically designed to provide insights into the biologic and behavioral correlates of HIV-1 transmission. Given the high prevalence of transmitted low-frequency mutations identified by our pilot project, these two established cohorts are logical populations in which to study the transmission of HIV-1 drug resistance and dynamics following HIV-1 acquisition. The OLA, developed by collaborator Dr. Lisa Frenkel, is a relatively inexpensive, high-throughput assay that can screen for HIV-1 drug resistance at 17 codons associated with primary drug resistance. It is an obvious choice, from among the more-sensitive HIV-1 drug resistance assays, for these studies.

**Aim #2:**

Detection of new mutations during ARV therapy

Source partners

OLA in:

PBMCs

Blood plasma

Genital secretions

Baseline HIV-1 drug resistance mutations detected

**Aim #3**:

Transmission of HIV-1 drug resistance

PIC enrollees

Baseline consensus sequencing and OLA in:

PBMCs

Blood plasma

Semen

OLA in PBMCs, blood and/or seminal plasma 6 and 12 months after HIV-1 infection

**Figure 5: Overview of the proposed research project and specific aims**

Baseline HIV-1 drug resistance mutations not detected

ARV therapy

OLA in PBMCs 6 and 12 months after ARV start

**Aim #1:**

Detection of mutations in untreated subjects over time

**Figure 5:** Baseline HIV-1 drug resistance testing will be performed using OLA and consensus sequencing on the first available specimens from subjects with acute and very early HIV-1 infection who enroll in the PIC cohort and from subjects previously enrolled in the cohort who meet eligibility criteria for study. ARV-naïve subjects with mutations detected at baseline will provide specimens to determine the detection of mutations over time (**Aim #1**). Subjects both with and without HIV-1 drug resistance at baseline who initiate ARV therapy during follow-up will provide specimens to determine whether increased levels of HIV-1 drug resistance mutations are detected in PBMCs during ARV therapy and whether low-frequency mutations impact the response to ARV therapy (**Aim #2**). Subjects enrolled in the PIC Cohort study will refer potential source partners to the PIC Partners study. Partners will be asked to provide blood and genital specimens in order to compare patterns of HIV-1 drug resistance within transmission pairs (**Aim #3**).

**5.b Study objectives**

**Aim #1: Determine whether the "level" of detection of HIV-1 drug resistance variants predicts the duration of detection of these variants over time since HIV-1 acquisition.**

Primary objectives:

1) Quantify the proportion of subjects with transmitted HIV-1 drug resistance that have mutations that remain detectable in PBMCs compared to blood plasma 6 and 12 months after HIV-1 infection.

2) Compare the proportion of subjects with low-frequency HIV-1 drug resistance that have mutations that remain detectable 6 and 12 months after HIV-1 infection compared to subjects with mutations detected by consensus sequencing at baseline.

Secondary objectives:

1) Identify subject characteristics associated with transmitted low-frequency HIV-1 drug resistance.

2) Compare the detection of mutations in blood and seminal plasma in male subjects over time.

3) Describe differences in the detection of specific drug resistance mutations (e.g. M184V) over time.

**Aim #2: Quantify the increase in detection of low-frequency mutations in PBMCs during ARV therapy.**

Primary objectives:

1) Compare the proportion of subjects with transmitted HIV-1 drug resistance that have new HIV-1 drug resistance mutations detected in PBMCs 6 and 12 months after the start of ARV therapy compared to subjects without transmitted HIV-1 drug resistance at baseline.

2) Evaluate time to virologic suppression in subjects with transmitted HIV-1 drug resistance compared to subjects without transmitted HIV-1 drug resistance.

Secondary objectives:

1) Explore whether the timing of ARV therapy impacts the increased detection of low-frequency mutations, time to virologic suppression, or time to virologic failure.

2) Explore whether specific mutations (e.g. M184V) are selected for by ARV therapy and are found at a greater level over time within treated subjects with and without HIV-1 drug resistance at baseline.

3) Explore whether specific HIV-1 drug resistance mutations are associated with significant differences in time to virologic suppression and time to virologic failure compared to subjects without transmitted HIV-1 drug resistance.

**Aim #3: Compare HIV-1 drug resistance patterns in PIC subjects and their source partners.**

Primary objective:

1) Quantify the probability of transmission of HIV-1 drug resistance mutations from source partners.

Secondary objectives:

1) Explore whether the probability of transmission differs for specific drug resistance mutations.

2) Explore whether HIV-1 drug resistance mutations are found at different levels in recipient partners compared to confirmed source partners.

**5.c Study design and data analysis**

**5.c.1 Aim #1: Determine whether the "level" of detection of HIV-1 drug resistance variants predicts the duration of detection of these variants over time since HIV-1 acquisition.**

Aim #1 will evaluate *the level* of detection of low-frequency HIV-1 drug resistance mutations in PBMCs, blood and seminal plasma from ARV-naïve subjects at baseline and over time. Because we anticipate that no more than 50% of male subjects will provide semen specimens and we will have few female subjects available for study, the study will be powered to compare transmitted HIV-1 drug resistance in PBMCs and blood plasma. We hypothesize that a smaller proportion of subjects will have low-frequency mutations that remain detectable compared to subjects with mutations detected by consensus sequencing at baseline, and a greater proportion of subjects will have mutations that remain detectable in PBMCs compared to blood and seminal plasma.

All subjects selected for study will have baseline testing to detect transmitted HIV-1 drug resistance using both OLA and consensus sequencing. Results of baseline testing will be combined with results from our pilot study to obtain a more precise estimate of the prevalence of transmitted HIV-1 drug resistance in Seattle. Logistic regression analyses will explore subject characteristics potentially associated with transmitted drug resistance including subject age; race/ethnicity; level of education; risk factor for HIV-1 acquisition; year of HIV-1 acquisition; use of methamphetamine, other illicit drugs, or erectile dysfunction medications; number of recent sex partners; and locations for meeting new partners (e.g. bathhouses, internet, etc) [188, 189].

**Table 7: Precision estimates for proportions of subjects with transmitted HIV-1 drug resistance**

| Sample size | 100 | 150 | 200 | 300 | 400 |
| --- | --- | --- | --- | --- | --- |
| 95% confidence interval (+/-) | .10 | .08 | .07 | .06 | .05 |

The sample size required for this study will be motivated by a longitudinal analysis that will compare the proportion of subjects with transmitted HIV-1 drug resistance who have mutations that remain detectable in PBMCs and blood plasma 6 and 12 months after HIV-1 infection. In contrast to a strategy that tests every specimen until mutations can no be longer detected, this testing framework will minimize the numbers of tests to be performed as part of this study and will inform the design of future testing strategies to determine more precisely the time to loss of detection of transmitted low-frequency HIV-1 drug resistance mutations.

**Table 8: Estimated number of subjects with mutations in PBMCs and plasma over time**

| **8a**: Distribution of subjects at baseline | | | |
| --- | --- | --- | --- |
|  | | plasma | |
|  |  | - | + |
| PBMCs | - |  | *14 (41%)* |
|  | + | *8 (24%)* | *12 (35%)* |

| **8b**: Distribution of subjects at follow-up | | | |
| --- | --- | --- | --- |
|  | | plasma | |
|  |  | - | + |
| PBMCs | - | 12 (30%) | 4 (10%) |
|  | + | 18 (45%) | 6 (15%) |

| **8b**: Distribution of subjects at follow-up | | | |
| --- | --- | --- | --- |
|  | | plasma | |
|  |  | - | + |
| PBMCs | - | *20 (25%)* | *16 (20%)* |
|  | + | *40 (50%)* | *4 (5%)* |

+ = subjects w/ ≥1 mutation or mixture.

- = subjects w/o mutations or w/ indeterminate results

**Table 8**: The number of subjects who would have transmitted drug resistance identified in PBMCs and plasma at follow-up (**Table 8b and 8c**) was estimated for power calculations *based on the distribution of mutations identified at baseline (****Table 8a****) from the pilot project.*

Power calculations were performed using a two-sided McNemar's exact test with significance level of 0.05. We assumed that the baseline distribution of subjects with mutations in PBMCs, plasma, or both would be similar to that found in the pilot study *(****Table 8a):*** *25% in PBMCs, 40% in plasma, and 35% having mutations in both PBMCs and plasma).* In these power calculations, we estimated the proportion of these subjects who would have mutations detected in PBMCs and/or plasma *at the* follow-up *evaluation* (**Table 8b and *8c***). If *mutations initially detected in PBMCs and plasma remain detectable in 100% and 25% of subjects, respectively, in the follow-up specimen,* 30% of subjects who had transmitted HIV-1 drug resistance *will* no longer have detectable mutations, 10% of subjects *will* have mutations detected in plasma only, 45% of subjects *will* have mutations detected in PBMCs only, and 15% of subjects *will* continue to have mutations detected in both PBMCs and plasma (***Table 8b***). *With this distribution,* we would have 84% power to determine whether a significant difference exists in the detection of mutations in PBMCs versus plasma over time with 40 subjects who had transmitted HIV-1 drug resistance detected at baseline.

*If the difference in loss of detection between PBMCs and plasma is less extreme, and 67% and 13% of mutations initially detected in PBMCs and plasma remain detectable in follow-up (****Table 8c****), then we would have 89% power to determine whether a significant difference exists in the detection of mutations in PBMCs versus plasma over time with 80 subjects who had transmitted HIV-1 drug resistance detected at baseline. Note that the McNemar's exact test takes account only of the distribution at follow-up and not changes over time; these calculations are therefore extremely conservative compared to the planned analysis described below.*

This sample size will be generated from previously enrolled subjects and from new enrollment. *There are 73 subjects among the entire PIC cohort who enrolled within 30 days of HIV-1 infection, who enrolled after 1996, and who have a median of 36 (range 0-139) months in follow-up.* Nine subjects studied through the pilot project were enrolled within 30 days of HIV-1 infection, had at least one low-frequency mutation identified, and have at least one year of follow-up in the cohort without ARV treatment. *Fifty-four subjects have enrolled in the cohort since the end of the pilot project, and we* anticipate that there will be an additional *15* subjects who will enroll before the start of this project, all of whom *will have been* enrolled within 30 days of HIV-1 infection. If we assume that *34*% of these subjects would have HIV-1 drug resistance mutations and *66% will remain ARV-naïve and in follow-up for at least one year*, we would expect that *15 previous-enrolled subjects will be eligible for study in Aim #1*. Prospective enrollment of *16* subjects with transmitted HIV-1 drug resistance in addition to the *24* previously-enrolled subjects would achieve the desired total sample size of 40. If *34*% of newly-enrolled subjects have transmitted HIV-1 drug resistance but *33*% of subjects initiate ARVs within 30 days after HIV-1 diagnosis, we would need to enroll *72* subjects by the end of the project period to achieve this sample size *(****Figure 5****).* Given our rate of enrollment in recent years, this number would be reasonably expected to accrue within the five years of the project.

Data obtained for this aim will be analyzed by logistic regression using generalized estimating equations (GEE) to account for correlated data and adjusting for the presence of mutations in PBMCs and/or plasma at baseline and the level of detection (<5%, 5-10%, 10-30%, 30-50%, or >50% of the viral quasi-species) of the mutation at baseline. We will also explore differences in the detection of mutations in seminal versus blood plasma and the detection of specific mutations (e.g. M184V) over time in PBMCs, blood and seminal plasma; however, we are unlikely to have sufficient power for these comparisons to perform definitive analyses.

**5.c.2 Aim #2: Quantify the increase in detection of low-frequency mutations in PBMCs during ARV therapy.**

The primary objectives of the study described in this aim will evaluate changes in the detection of low-frequency mutations in PBMCs over time during ARV therapy and the virologic consequences of ARV therapy in the setting of transmitted low-frequency HIV-1 drug resistance. We hypothesize that, over time, additional mutations will be detected in a greater proportion of treated subjects with transmitted drug resistance compared to treated subjects without HIV-1 drug resistance detected at baseline, because the relative level to which viral replication is suppressed will be diminished in the former group. Secondary analyses will explore associations between specific HIV-1 drug resistance mutations, the interval between HIV-1 acquisition and the start of ARV therapy, the increased detection of HIV-1 drug resistance mutations, and treatment outcomes including time to virologic suppression and time to virologic failure.

This longitudinal study will evaluate subjects enrolled in the PIC cohort within 30 days of the estimated date of HIV-1 infection, who initiate ARV therapy at any point following the baseline evaluation, and who achieve undetectable HIV-1 RNA levels (less than 50 copies/mL) within six months after the start of ARV therapy. OLA will be performed using PBMCs collected at the visit immediately prior to the start of ARV therapy (unless this visit is within 4 weeks from the baseline specimen) and in specimens obtained 6 and 12 months following the start of treatment. The detection of a new mutation will be defined as any mutation detected during follow-up that was never previously detected. Additional analyses will use the semi-quantitative nature of OLA to evaluate the changes in levels of mutations during ARV treatment (i.e. <5%, 5-10%, 10-30%, 30-50% >50% of the viral quasi-species) and the reappearance of mutations that had been detected at baseline but were lost to detection prior to the start of ARV therapy in the absence of ARV selection pressures.

**Table 9: Sample size needed in each arm to detect a difference in proportion of subjects with new low-frequency mutations**

|  | | Percent of subjects with low-frequency mutations at baseline who have new mutations detected after start of ARVs | | | |
| --- | --- | --- | --- | --- | --- |
|  |  | 20% | 30% | 40% | 50% |
| Percent of subjects with no mutations at baseline who have new mutations detected after start of ARVs | 10% | *316/158* | *102/51* | *54/27* | *35/18* |
|  | 20% | *-* | *461/231* | *133/67* | *66/33* |
|  | 30% | *-* | *-* | *559/280* | *153/77* |
|  | 40% | *-* | *-* | *-* | *609/305* |

alpha = 0.05, power = 80%

The sample size for this aim will be motivated by a two-sample test of proportions that will compare the percentages of subjects with low-frequency mutations at baseline who have new mutations detected during ARV therapy compared to the percentage in subjects who had no mutations detected at baseline (**Table 9**). Estimates for the sample size calculations were informed by two small studies that identified increased numbers of HIV-1 drug resistance mutations in two (22%) of nine [32] and six (40%) of 15 [33] ARV-naïve subjects during ARV therapy. Based on these calculations and assuming that one-third of subjects will have transmitted drug resistance, a sample size of *53* treated subjects (*18 subjects with and 35 without drug resistance detected at baseline*) will have 80% power to detect a significant difference in the two groups if 50% of the subjects with baseline mutations and 10% of the subjects without baseline mutations have additional HIV-1 drug resistance mutations detected in PBMCs after the start of ARV therapy. There were 20 subjects who were studied as part of the pilot project who enrolled in the PIC Cohort study within 30 days of HIV infection, received ARV therapy for at least six months, and achieved virologic suppression. Another *twenty* subjects have enrolled in the PIC Cohort since the pilot study began and have received treatment *for at least six months*. If we estimate that *33*% of newly-enrolled subjects will initiate ARV therapy *in the first year of* follow-up *and assume a 10% loss-to-follow-up rate in the first year*, we would need to enroll *42* subjects in addition to these *40* subjects in order to achieve the desired sample size of *53* treated subjects (***Figure 5***)*.*

If no difference is identified between groups with and without transmitted HIV-1 drug resistance, an estimate of the proportion of all subjects with primary HIV-1 infection who have new HIV-1 drug resistance mutations detected in PBMCs during ARV therapy would be a novel finding of this study. **Table 10** shows the maximum 95% confidence intervals associated with varying sample sizes used to estimate the proportion of subjects who have new HIV-1 drug resistance mutations detected. If new HIV-1 drug mutations are not detected in any subjects during study follow-up, a sample size of 35 subjects would exclude the possibility that the true proportion of subjects with new mutations detected in PBMCs during virologic suppression is greater than 10%. If we identify significant numbers of subjects who have new HIV-1 drug mutations detected during follow-up, we will use logistic regression analyses to explore whether the detection of additional HIV-1 drug resistance mutations is associated with transmitted HIV-1 drug resistance, the timing of ARV therapy, the HIV-1 RNA level at the start of ARV therapy or rate of the initial viral decay, or treatment adherence.

**Table 10: Precision estimates for proportions of subjects with new drug resistance mutations detected**

| Sample size | 10 | 20 | 30 | 40 | 50 | 100 |
| --- | --- | --- | --- | --- | --- | --- |
| 95% confidence interval (+/-) | .31 | .23 | .19 | .16 | .14 | .10 |

With the increased study size compared to the pilot project, we will perform a definitive analysis to assess the impact of low-frequency mutations on time to virologic suppression (defined as HIV-1 RNA less than 50 copies/mL). Survival analyses will evaluate the time to virologic suppression among subjects with transmitted low-frequency HIV-1 drug resistance mutations detected who are on ARV regimens with fewer than three active agents compared to subjects without transmitted HIV-1 drug resistance, controlling for the baseline HIV-1 RNA level. If a significant difference is detected, we will use additional Cox proportional hazard models to explore characteristics (e.g. HLA type, specific mutation or class of ARV therapy, and timing of ARV treatment) that might impact time to virologic suppression.

We will also perform a survival analysis evaluating the time to virologic failure among subjects with transmitted low-frequency HIV-1 drug resistance mutations who are on ARV regimens with fewer than three active ARV agents compared to subjects without transmitted HIV-1 drug resistance, controlling for baseline HIV-1 RNA level and the timing of initiation of ARV therapy. For purposes of this analysis, virologic failure will be defined as any one of the following:

1) Failure to suppress viral replication (HIV-1 RNA greater than 400 copies/mL after 24 weeks or greater than 50 copies/mL on two occasions after 48 weeks)

2) Addition or substitution of ARVs because of delay in viral suppression

3) Detectable HIV-1 RNA (greater than 400 copies/mL) on two consecutive occasions following viral suppression

In the pilot study, only *four subjects were* observed to have virologic failure during follow-up. It is likely that, even with a larger sample size and longer follow-up, we will have limited power to detect associations between the time to virologic failure with either transmitted HIV-1 drug resistance or the timing of ARV therapy. Time to virologic suppression has been evaluated by other studies [52, 53, 56, 190], and this outcome and other markers of early treatment response have been associated with longer-term treatment outcomes [191-193]. This issue will be discussed further under **D.6 Limitations, alternatives, and approaches to anticipated problems.**

If significant numbers of any one specific mutation (e.g. M184V) are identified, we will explore the impact of that mutation on the virologic outcomes described above. Further evaluations of the clinical impact of low-frequency HIV-1 drug resistance mutations will also be performed.

**5.c.3 Aim #3: Compare HIV-1 drug resistance patterns in PIC subjects and their source partners.**

This cross-sectional study will evaluate the prevalence and patterns of HIV-1 drug resistance in partner-pairs and compare results of HIV-1 drug resistance testing in the PBMCs, blood plasma, and genital secretions (when available) from the confirmed source partner with test results in PBMCs and blood plasma from the recipient partner. We hypothesize that HIV-1 drug resistant variants will have lower "transmission fitness" compared to wild-type variants, and drug resistance will occur less frequently and at lower levels in the PBMCs and plasma from PIC subjects than in the PBMCs, blood plasma, and genital secretions from their confirmed source partners. Because the most common risk for HIV-1 acquisition reported among our cohort is receptive anal intercourse, the ideal comparison would evaluate the results of HIV-1 drug resistance testing in semen specimens from every source partner. However, not all potential source partners collect genital secretions for study. As previous studies have shown that there is moderate concordance between HIV-1 drug resistance testing results in blood and semen [61-63], our proposed comparison using the results of resistance testing in the blood of source partners should have relevance.

For every subject enrolled in the PIC Cohort study, we will attempt to identify and study all source partners through the methods described below in **5.d.2 PIC Partners study** and **5.e Laboratory methods**. If OLA is not performed as part of the other studies previously described in this application, OLA will be performed on the first available PBMC and plasma specimens from PIC enrollees and on the PBMC, blood plasma, and genital secretions from confirmed source partners. We hypothesize that many but not all mutations in the source partner will be identified in the recipient partner and that low-frequency mutations detected in the recipient partner will be present at lower levels compared to their levels in the source partners. Secondary analyses will explore whether the probability of transmission differs between specific HIV-1 drug resistance mutations.

Analysis for the primary objective will be calculated as a binomial probability of the number of mutations transmitted from each source partner to the recipient partner, conditional on the number of mutations in the source partner. This accounts for the fact that source partners will undoubtedly have different numbers of HIV-1 drug resistance mutations. For the purpose of sample size calculations, we assume that the number of mutations in the source partners follows a Poisson distribution with the expected number of mutations being two. Power calculations are based on simulations, repeated 1000 times. Each simulation estimates the binomial probability of transmitting a mutation conditional on the number of mutations in the source partners, and calculates the associated 95% confidence intervals based on normal approximation for the estimated probability. The type II error is calculated as the number of simulation replicates where the 95% confidence interval contains the value of 1, when the simulated transmission probability is less than 1.

Using the simulation analyses described above, we will have 82% power to detect a transmission probability of all source partner mutations of 80% or less if we have 17 confirmed partner-pairs in which the source partner has at least one HIV-1 drug resistance mutation. *Overall, the subset of recipient partners who had resistance identified by OLA (4/12=33%) was similar to the population studied in the pilot project. Of the 18 confirmed partner pairs without complete OLA testing, we would expect 6 to 18 source partners to have HIV-1 drug resistance. Based on our prior experience with confirming putative source partners, we would expect that the relationship of 14 of the remaining 17 partner-pairs would be confirmed, and 5 to 17 of these source partners would have HIV-1 drug resistance. We would therefore need to enroll at most 21 (and likely fewer) new putative source partners in order to have at least 6 additional confirmed source partners with HIV-1 drug resistance.*

To further explore which specific mutations are more likely to be transmitted and result in subsequent HIV-1 infection, we will conduct a logistic regression analysis with generalized estimating equations to account for correlation of the presence of different mutations within the same source partner. In this analysis, each individual mutation will be evaluated, with the outcome in the regression model; transmitted to partner Yes/No. Indicator covariates will be used to identify specific mutations. To account for the potential loss of detection of mutants over time, an exploratory analysis will adjust for the estimated time between the transmission event and the dates samples are obtained from the transmission pair for HIV-1 drug resistance testing.

We will also use the semi-quantitative nature of OLA to categorize HIV-1 drug resistance mutations as comprising <5%, 5-10%, 10-30%, 30-50%, and 50-100% of the viral quasi-species. We will compare the level of specific HIV-1 drug resistance mutations in the source and recipient partners to identify, for example, whether a mutation exists as 5-10% of the population in the source partner but 10-50% in the recipient. Analyses will control for HIV-1 RNA levels at the time of HIV-1 transmission.

**5.d Study populations and evaluations**

**5.d.1 UW Primary Infection Clinic (PIC) Cohort study**

As described in **4. Preliminary Studies**, 319 subjects with acute and early HIV-1 infection have been enrolled in an observational, natural history study through the PIC. When available, we will use stored samples from subjects who were enrolled in the PIC Cohort study after 1996 and who otherwise meet criteria for one of the studies described in **5.c Study design and data analysis**. Subjects previously enrolled into the cohort who meet these criteria and who remain in active follow-up will continue to be followed.

In order to be eligible for ongoing enrollment in the PIC, individuals must be at least 16 years old and have acute or very early HIV-1 infection, defined as one of the following:

• plasma HIV-1 RNA greater than 2000 copies/mL with a negative HIV-1 enzyme immunoassay (EIA) or negative/indeterminate Western blot

• positive EIA with a documented negative HIV-1 antibody test within the last four months, a credible history of exposure, symptoms consistent with the acute retroviral syndrome [164], and a plasma HIV-1 RNA greater than 400,000 copies/mL.

Results of rapid HIV-1 antibody testing will not be sufficient to document acute HIV-1 infection, because we have identified several persons who tested HIV-negative by a rapid HIV-1 antibody test but who were HIV-positive by both a 1^st^ or 2^nd^ generation EIA and Western Blot confirmatory assays (Fiebig stage V *and VI*) [44]. Subjects must be screened for enrollment in the cohort within 30 days of the estimated date of HIV-1 infection, defined as the onset date of symptoms consistent with the acute retroviral syndrome or, for asymptomatic subjects, the midpoint between the last negative and first positive HIV-1 tests.

The schedule of evaluations (**Table 11**) for newly-enrolled subjects will be similar to the schedule of evaluations that has previously been employed for all subjects enrolled in the PIC cohort with the removal of the study visit (V10) 32 weeks after study enrollment to minimize blood volume and to reduce costs. Study visits will occur at more frequent intervals than required as a part of this application in order to have specimens available for more detailed analyses related to this and other projects in the future.

**Table 11: PIC Cohort study (The virologic and immunologic events in acute/early HIV-1 infection): schedule of e**valuations

| Visit # | V1  Screen | V2  Entry | V3  Wk 1 | V4  Wk 2 | V 4.5  Wk3 | V5  Wk 4 | V6  Wk 8 | V7  Wk 12 | V8  Wk 16 | V9  Wk 24 | V11  Wk 48 | Every 6  Months |
| --- | --- | --- | --- | --- | --- | --- | --- | --- | --- | --- | --- | --- |
| Clinic Visit | X | X | X | X | X | X | X | X | X | X | X | X |
| Informed Consent | X | X |  |  |  |  |  |  |  |  |  |  |
| H&P |  | X |  |  |  |  |  |  | X |  | X | X |
| HIV-1 antibody | X | X |  | X^1^ |  | X^1^ | X^1^ |  | X^1^ | X^1^ |  |  |
| HIV-1 RNA testing | X | X | X | X | X | X | X | X | X | X | X | X |
| CBC/T Cell Subsets | X | X |  | X |  | X | X | X | X | X | X | X |
| Genital secretions^2^ |  | X | X | X | X | X | X | X | X | X | X | X |
| STI screening^3^ |  | X |  |  |  |  |  |  |  |  |  | X |
| Blood volume (mL) | 118 | 118 | 8.5 | 64 | 8.5 | 118 | 118 | 58 | 118 | 118 | 180 | 180 |
| Length of visit (min) | 60 | 90 | 20 | 30 | 20 | 30 | 30 | 30 | 45 | 45 | 45 | 45 |

H&P: history and physical exam; CBC: complete blood count

Note: If participants initiate ARV therapy, they will restart the schedule of evaluations with a repeat Entry visit.

^1^After the Entry Visit, HIV-1 antibody testing to be followed only until a participant has a confirmed positive result.

^2^Optional procedure. Participating subjects will be asked to collect genital secretions at every study visit except for day 3.

^3^Subjects will be offered screening for sexually transmitted infections (STIs) at study entry and at yearly intervals. Screening could include: blood for syphilis and HSV serologies, pharyngeal swab for gonorrhea culture, urine for NAAT for gonorrhea and chlamydia, and rectal swab for culture for gonorrhea and chlamydia. These will be transported by courier to the PHSKC laboratory.

At the initial visit, after obtaining informed consent, subjects will be asked questions about their medical history and about risk factors for HIV-1 acquisition, sex partners, and injection drug use. Subjects will be screened for HIV-1 infection, including HIV-1 antibody and HIV-1 RNA testing, and blood will be stored for future testing. At the Entry visit, if the subject is eligible for enrollment, s/he will be asked to refer potential source partners for enrollment into the PIC Partners study. A physical examination will be performed, including documentation of circumcision status. Blood will be drawn at this visit and at subsequent visits for clinical tests (i.e. CD4^+^ T-cell count and HIV-1 RNA level) as indicated in the schedule of evaluations. Blood, semen, and other specimens that are not utilized for the projects detailed in this application will be available for immunologic and virologic investigations to be funded by other project and program grant applications that are currently in process. If subjects initiate ARV therapy during the study, they will restart the schedule of evaluations, beginning with the Entry visit (V2). During follow-up, subjects will be asked questions about continued risk behaviors, ARV treatment and adherence, other concomitant medications, signs/symptoms, and diagnoses.

Men will be instructed not to ejaculate for 48 hours prior to collection of semen specimens, to ejaculate directly into the sterile specimen container, and to use no lubricant except for water. If collected at home, the subject will bring the specimen to the PIC within one hour after collection. Whether collected in the PIC or brought to the PIC after collection, the specimen will be sent immediately to the UW Clinical Retrovirology Laboratory.

**5.d.2 PIC Partners study**

As part of the UW PIC Cohort and PIC Partners studies (described in **4. Preliminary Studies**), we have, to date, previously identified a total of *36* partner-pairs and have confirmed *19* transmissions; confirmation of remaining 17 unconfirmed partner-pairs is pending. PIC Partners continue to be recruited and enrolled through referrals from subjects with primary HIV-1 infection enrolled into the UW PIC Cohort study (**Figure 6**).

**Figure 6: Methods for recruitment and identification of transmitting (source) partners for PIC Partners study**

PIC enrollee presents with primary HIV-1 infection, is eligible, and consents to PIC Cohort study.

PIC enrollee identifies all partners with whom s/he had contact in the 3 months prior to the estimated date of HIV-1 acquisition.

Potential source partner is notified by PIC enrollee or by PIC staff and consents to PIC Partners study.

PIC Partner is found to be infected with HIV-1, and viral sequences of PIC enrollee and Partner show high degree of homology.

When available, we will use stored samples for Aim #3 from subjects who previously enrolled in the PIC Partners study. We will also continue to identify putative source partners of new subjects enrolled into the PIC Cohort study. Persons will be eligible for screening if they are at least 18 years old and were identified by the PIC enrollee to be a sex partner or needle-sharing partner in the three months prior to the PIC enrollee's estimated date of HIV-1 acquisition. Enrollees can refer partners or have referral orchestrated by PIC clinicians. If referral is performed by PIC staff, partner notification follows procedures outlined in the Washington State HIV Partner Notification Guide, and the identity of the PIC enrollee is not revealed to the potential source partner. Contact with all eligible partners of PIC enrollees will be attempted, and the schedule of evaluations (**Table 12**) will be similar to the current PIC Partners schedule of evaluations. Blood and other specimens not utilized for the aims detailed in this application will be stored to be available for future immunologic and virologic investigations.

**Table 12: PIC Partners study schedule o**f evaluations

|  | Visit #1  Screening | Visit #2  Entry |
| --- | --- | --- |
| Informed Consent | X |  |
| Screening Questionnaire | X |  |
| HIV-1 testing (antibody, LS-EIA, Western blot, & plasma HIV-1 RNA) | X |  |
| Vital signs/weight |  | X |
| History and Physical Exam |  | X |
| CD4+ T-cell count/subsets |  | X |
| Immunology panel (HLA typing, CTL assays) |  | X^1^ |
| Serum storage |  | X^1^ |
| Plasma/PBMCs storage |  | X^1^ |
| Genital secretions |  | X^2,3^ |
| *STI screening^4^* |  | *X* |
| Blood volume (mL) | 15.5 | 111 |
| Length of visit (min) | 60 | 60 |

^1^Specimens will be collected and stored for future testing. More detailed laboratory studies will only be performed if sequence analysis confirms that transmission occurred from the PIC Partner to the PIC enrollee.

^2^Collection of semen specimens is recommended but optional.

^3^An additional 17mL of blood will be collected during a 20 minute visit if the collection of semen specimens is >4 days after Visit #2.

*^4^Subjects will be offered screening for sexually transmitted infections (STIs) at study entry, including blood for syphilis and HSV serologies, pharyngeal swab for gonorrhea culture, urine for NAAT for gonorrhea and chlamydia, and rectal swab for culture for gonorrhea and chlamydia. These will be transported by courier to the PHSKC laboratory.*

At the initial visit, partners not known to be HIV-positive will be informed of their exposure, asked about symptoms consistent with acute or established HIV-1 infection, and screened for HIV-1 infection. Partners will complete a behavioral questionnaire to ascertain their knowledge of their HIV-1 status and to quantify sexual and needle-sharing exposures to the PIC enrollee. If the partner is found to meet the above criteria for acute or early HIV-1 infection, s/he will be screened for additional studies available through the PIC.

Only HIV-positive partners will complete the additional evaluations and specimen collections listed in **Table 12** as Visit #2 (Entry). This visit may be combined with Visit #1 (Screening) for partners previously known to be HIV-positive. HIV-positive partners will be asked detailed information about their past medical history, including dates of prior HIV-1 testing (to estimate the date of HIV-1 infection), ARV history and previous opportunistic conditions. A physical examination will be performed, including documentation of circumcision status. Blood will be drawn for a CD4^+^ T-cell count and storage of serum, plasma, and PBMCs. Viral sequencing will be done in the Mullins laboratory to confirm the transmission relationship between partners. Collection of genital secretions will be recommended but optional for both male and female source partners. There is no longitudinal follow-up; we anticipate that all partners should complete the evaluations within a few weeks.

Collection of semen specimens for the PIC Partners Study will be performed as described under **5.d.1 UW Primary Infection Cohort**, above. Blood will be drawn for plasma and PBMCs if semen is collected more than four days after Visit #2 (Entry). For women, the methods for collection and processing are as described by Reichelderfer et al [194]. After removing visible blood or clots with a swab, endocervical canal fluid is collected using three filter paper strips (TearFlo^TM^). Each strip is placed into the cervical os, and cervical canal fluid is absorbed past the 10mm mark on each strip (15μL). Strips are removed, cut at the 10mm mark, and placed into vials containing 225 μL of guanidinium solution. Genital secretions are collected by cervical-vaginal lavage (CVL) using 10ml of sterile non-bacteriostatic saline, and endocervical canal cells are collected by cytobrush. The CVL fluid is put on ice and sent immediately to the UW Clinical Retrovirology laboratory.

**5.e Laboratory methods**

**5.e.1 Routine laboratory testing**

Routine tests including complete blood counts, lymphocyte subsets, HIV-1 EIA and Western Blot assays will be done by the UW Department of Laboratory Medicine Laboratory using standard techniques. Lymphocyte subsets will be determined using flow cytometry in a lab certified by the NIAID’s Immunology Quality Assurance Program. The SeaPIP Virology Core Laboratory will continue to separate plasma and cells and serve as the central storage facility for the specimens collected for this protocol.

**5.e.2 HIV-1 RNA quantification in blood plasma and genital secretions**

Blood plasma HIV-1 RNA quantification is performed by an independently-validated real-time RT-PCR amplification assay with lower limits of detection equal to 50 copies/mL [195]. Results of specimens collected between 1996 and 2002 were initially tested with branched DNA (bDNA) assays with lower limits of detection of 50 and 500 copies/mL (Chiron Corporation, Emeryville, CA). When specimens were available, results censored at 500 copies/mL have been re-tested using an ultra-sensitive reverse transcription polymerase chain reaction (RT-PCR) assay (Roche, Branchburg, NJ) or the real-time RT-PCR assay. Since 2002, all specimens have been evaluated by an RT-PCR assay.

Semen is processed within two hours of collection [196]. Specimens are diluted to reduce viscosity and centrifuged to separate the seminal plasma and cell pellet. Boom (silica) extraction is performed to minimize effect of inhibitory factors, and HIV-1 RNA is quantified using the real-time RT-PCR assay (lower limit of detection equal to 300 copies/mL) or Roche Amplicor HIV-1-1 Monitor versions 1.0 or 1.5 (lower limit of detection equal to 200 copies/mL) [197]. For both blood and semen, specimens with HIV-1 RNA levels below the lower limits of detection will be assigned censored values.

For women, CVL fluid is transported on ice and centrifuged after arrival at the laboratory, and the supernatant is frozen at –80^o^C. The cytobrush is put into PBS and agitated, the cells are separated by centrifugation, and the cell pellet is stored at –80^o^C. These specimens will be used for RNA quantitation and OLA.

**5.e.3** RT-PCR and PCR for genotyping of HIV-1 *pol*

RNA is extracted from 0.2 ml of plasma, as previously described [145]. DNA is extracted from PBMC using the Puregene Cell and Tissue kit (Gentra Systems, Inc., Minneapolis, MN) according to manufacturers' instructions. Ten μl of RNA extracted from plasma is reverse transcribed using the GeneAmp RNA PCR Core kit (Applied Biosystems, Foster City, Calif.). The first-round PCR of cDNA or of DNA extracted from PBMC is carried out in a 50-μl reaction mixture containing 10 μl of cDNA or 1 μg DNA, 1× PCR buffer, 1.5 mM MgCl_2_, 0.2 mM deoxynucleoside triphosphates, 20 pmol of the primers PRA and RTA [145], and 2.5 U of Taq DNA polymerase (Sigma-Aldrich Corp., St. Louis, Mo.). Cycling conditions as described previously [145] with a final extension at 72°C for 7 min. Second-round PCR contains 2 μl of first-round product and 20 pmol of primers PRB and RT3 [145]. The amplicon, a 1,193-bp DNA fragment extending from nucleotide 1of HIV-1-pro to nucleotide 711 of the RT gene according to the HXB-2 numbering system, is visualized in a 1% agarose gel with ethidium bromide staining. The amount of DNA resulting from nested PCR is estimated based on the band intensity relative to a DNA Mass Ladder on the same gel. Further testing is performed on samples having DNA concentrations between12 to 20 ng/μl.

**5.e.4 Consensus sequencing**

The PCR amplicon is purified by treatment with ExoSAP-IT (Amersham Biosciences, Piscataway, N.J.). Four primers are used in bidirectional sequencing of the PCR products, including protease primers PRB and PR2 and RT primers RT4 and RT3. PCR amplicon is added to a sequencing reaction using fluorescence-labeled dideoxynucleotide chain terminators (AB PRISM Big Dye Terminator Cycle Sequencing Ready Reaction kit, version 1.1; Applied Biosystems). Cycling conditions were 96°C for 30 s, 50°C for 15 s, and 60°C for 4 min for 25 cycles. Samples are submitted to a core sequencing facility where excess dye terminators are removed and samples are submitted to a 3730XL automated sequencer (Applied Biosystems). Sequences are analyzed by Sequencher, version 3.0 (Gene Codes Corp, Ann Arbor, Mich.), with the presence of major and minor peaks recognized when visible in each of the bidirectional strands. The Stanford HIV-1seq Sequence Analysis Program [151] identified mutations within the sequences. As part of quality control procedures, assembled sequences are reviewed by a second person or at a later date to confirm results. To monitor for cross-contamination, genotypes generated are compared to all of those produced as part of this study by aligning them in ClustalX and reviewing the neighbor-joining phylogenetic tree. If a specimen more closely matches an unrelated specimen, a new aliquot is extracted and sequenced. Biannual verification is performed in the laboratory using blinded specimens provided by Rush's Virology Quality Assurance Laboratory.

**5.e.5 Oligonucleotide ligation assay (OLA)**

OLA targets specific wild-type or mutant codons for primary HIV-1 drug resistance mutations. Wild-type probes are labeled with digoxigenin *(D),* and mutant probes are labeled with fluorescein (*F*). In the following example, K103N, which confers resistance to non-nucleoside reverse transcriptase inhibitors (NNRTIs), is a mutation from the wild-type sequence AAA (lysine: K) to AAC (asparagine: N). The oligonucleotide probes, labeling, and sequence of steps used to detect this mutation by OLA are as follows:

5' 3'

common probe: **AAA**-TCA-GTA-ACA-GTA-CTG-GAT-GTG-GGT-*Biotin*

wild-type probe: *D*-AC-ATC-CCG-CAG-GGT-TAA-AAA-AAG-**AAA**

mutant probe: *F*-AC-ATC-CCG-CAG-GGT-TAA-AAA-AAG-**AAC**

The mutant or wild-type probe and common probe anneal to adjacent complementary sequences in the PCR product of the region of *pol* encoding reverse transcriptase. When the probes are perfectly complementary at the two bases on either side of the ligation site, the probes are covalently bound by DNA ligase.

*F*-AC-ATC-CCG-CAG-GGT-TAA-AAA-AAG**-AAC AAA**-TCA-GTA-ACA-GTA-CTG-GAT-GTG-GGT -*Biotin*

**↓ ↓**

…TG-TAG-GGC-GTC-CCA-ATT-TTT- TTC- TTG - TTT -AGT-CAT- TGT-CAT-GAC-CTA-CAC-CCA…

**Figure 7: Capture of biotinylated**

**probes on the ELISA plate [1]**

*F*-AC-ATC-CCG-CAG-GGT-TAA-AAA-AAG**-AAC - AAA**-TCA-GTA-ACA-GTA-CTG-GAT-GTG-GGT -*Biotin*

…TG-TAG-GGC-GTC-CCA-ATT-TTT- TTC- TTG - TTT -AGT-CAT- TGT-CAT-GAC-CTA-CAC-CCA…

The ligation products are then captured onto a streptavidin-coated ELISA plate (**Figure 7**), and alkaline phosphatase (AP) labeled anti-fluorescein antibodies and horseradish peroxidase (POD) labeled anti-digoxigenin antibodies are added sequentially. The AP (magenta) and POD (yellow) labeling is quantified by optical density (O.D.) using an ELISA plate reader. In the example above, only mutant virus (magenta) would be detected; however, mixtures of both wild type and mutant viruses are detectable by OLA. Indeterminate reactions occur 2-7% of the time and are typically a result of failure of the ligation reaction due to polymorphisms usually occurring at or adjacent to the two bases to either side of the ligation site. Under those circumstances, no color is produced.

**Figure 8: Examples of results of OLA testing for M184V**

**Figure 8:** Examples of optical densities (ODs) from negative controls; 2%, 5%, and 100% dilutions of cloned mutant virus; and subject samples are shown. All samples are tested in duplicate. A sample is identified as mutant (grey bar) if the mean OD for the two samples at 490nm is greater than the OD for the 5% mutant. This threshold is indicated by the horizontal line. If this requirement is met, but the OD is less than 0.2, the sample is re-tested. If the re-tested sample is again greater than the OD for the 5% mutant, a mutant variant is presumed present. 95% confidence intervals for ODs are indicated by vertical hash marks.

The mutations to be tested include K65R, K70R, L74V, M184V, T215F/Y (NRTI resistance); K103N, Y181C, and G190A (NNRTI resistance); and D30N, I50V, V82S/A/T, I84V, N88D, and L90M (PI resistance). For the semi-quantitative assays, comparisons will include 2%, 5%, 10%, 30% and 50% dilutions of known mutant virus after DNA viral quantification (**Figure 8**). For quality control purposes, duplicates of test specimens and all controls [water (negative control), mutant (positive control), and wild-type (positive control)] are run on the same plate. Biannual verification of the assay is performed using blinded specimens previously identified by consensus sequencing.

**5.e.6 Molecular cloning**

The molecular cloning techniques for the 625 base-pair HIV-1 *env* C2V5 region (HXB2 positions 7021-7646) have been previously described [198]. Briefly, for each patient, plasma will be used to isolate viral RNA, which is then reverse transcribed to cDNA and used as a template for DNA amplification by a nested PCR. The two sets of primers used, ED5 and ED12 and ES7 and ES8, have also been previously described [199].

**5.e.7 Heteroduplex tracking assay (HTA)**

The technique for HTA has also been previously described [198]. PCR reactions containing multiple variants from the potential source partner are radio-labeled to serve as the comparison clone (driver) in a mixture containing a 100 fold excess of PCR products from the potential source partner. The heteroduplex products are then examined on an acrylamide gel to determine whether closely related sequences (fast migrating heteroduplexes) are found in the two individuals.

**5.e.8 DNA cloning, sequencing, and analysis**

In instances in which homoduplexes or nearly identical heteroduplexes are demonstrated, DNA sequencing will be performed to precisely define these relationships. Ten to fifteen clones per individual, derived from PCR products used for HTA, will be inserted into plasmid vectors for amplification and sequencing. Quality control for the presence of sample mix-ups and carry-over contamination will be strictly enforced. Sequences are aligned with the CLUSTALW program and then manually edited. Pairwise evolutionary nucleotide distances and maximum-likelihood phylogenetic trees will be estimated, and partnerships will be evaluated for linkage as described by Gottlieb et al. [200].

**5.f Limitations, alternatives, and approaches to anticipated problems**

**5.f.1 Limitations of the assays**

Although OLA is more sensitive than consensus sequencing, the assay does have limitations. In contrast to other methods of HIV-1 drug resistance testing, the loci of interest must be pre-specified, and other codons in addition to those mentioned in **3.f** may need to be added in the future. The assay is tolerant of a moderate number of non-specific polymorphisms in the region complementary to the probes, but indeterminate reactions can occur when polymorphisms occur within two bases to either side of the ligation site. This may occur more frequently in non-subtype B virus [152, 153], as the OLA probes were designed based on subtype B sequences. However, the vast majority of newly-infected individuals in Seattle & King County and all subjects previously tested in the PIC cohort have subtype B virus. Other probes have been optimized for subtypes C and A/E and could be used if we enroll subjects with these types of viruses.

Although there are other assays that have greater sensitivity than OLA, the use of OLA has many advantages. OLA has relatively low cost, high throughput, and high specificity. If it becomes clearer that the low-frequency HIV-1 drug resistance mutations detected by OLA are clinically relevant, OLA could be modified [201] or other more-sensitive assays could be used in future studies to determine the prevalence and clinical impact of HIV-1 drug resistance mutations that were at concentrations below that detectable by standard OLA. Further validation of OLA may also be performed to confirm the low-level detection of mutations using real-time PCR [202-204] or another more-sensitive assay.

With the intermediate sensitivity of OLA, the analyses described in Aim #2 will not be able to distinguish whether the detection of additional mutations during ARV therapy was due to the selection of transmitted HIV-1 drug resistance mutations that were at extremely low levels at the start of therapy or whether new mutations developed through incomplete viral suppression and continued viral replication. Future studies could use ultra-sensitive assays to re-test baseline specimens in order to determine whether the additional HIV-1 drug resistance mutations had been transmitted or were acquired during ARV therapy.

**5.f.2 Use of ARV therapy**

The projects proposed in this application will evaluate both treated and untreated subjects with acute HIV-1 infection. If a substantial fraction of untreated enrollees start ARV therapy or treated subjects stop ARV therapy, these subjects will not be able to contribute to the projects as planned. However, our experience suggests that most PIC participants continue with their initial choice or deferral of ARVs. The estimates for numbers and choice of subjects for the prospective studies assume a 10% per year loss to analysis (whether from loss to follow-up or change in ARV status). From an ethical viewpoint, it is important that the pros and cons of ARV therapy for acute HIV-1 infection are presented in an unbiased fashion and allow for use of ARV therapy when indicated (progressive HIV-1 infection or severe CD4^+^ T-cell depletion).

**5.f.3 HIV-1 superinfection**

Superinfection, the acquisition of a second virus following acute HIV-1 infection, is a rare phenomenon that is thought to occur with greater frequency during primary HIV-1 infection [205-207]. Superinfection could lead to a sudden increase in HIV-1 drug resistance mutations if superinfection occurs with a virus having HIV-1 drug resistance mutations, or it could lead to a decrease in HIV-1 drug resistance if superinfection occurs with a wild-type virus that overgrows a drug resistant virus that resulted in the initial acquisition of HIV-1 infection [18, 206, 207]. This cohort will be screened for superinfection in collaboration with another project (NIH AI 44734: Occurrence and Outcome of Dual HIV-1 Infection; PI: James Mullins). If subjects are found to have superinfection and not a dual infection that was present at the time of HIV-1 acquisition, they will be censored at the time of detection of the second viral population.

**5.f.4 Power**

As previously mentioned, this study has limited power for the analysis comparing the time to virologic failure among subjects with and without transmitted HIV-1 drug resistance who initiate ARV therapy. We will instead perform an analysis comparing the time to virologic suppression, which has been used as a surrogate outcome by other seminal studies that evaluated the impact of transmitted drug resistance on the response to ARV treatment [52, 53, 56, 190]. In addition, the time to virologic suppression and other markers of early treatment response have been associated with longer-term treatment outcomes [191-193]. If our results suggest that transmitted low-frequency HIV-1 drug resistance has an adverse impact on response to ARV therapy, a randomized clinical trial will be designed to definitively answer these questions.

These studies will also not have sufficient power to identify specific mutations that may be particularly clinically relevant (e.g. K103N) because of the infrequent transmission of any one particular HIV-1 drug resistance mutation and the frequent co-transmission of mutations. Similarly, we will not be able to determine if there are differences in the rate of loss of detection or compare rates of transmission between specific mutations.

**5.f.5 Ability to enroll subjects with primary HIV-1 infection and their partners**

As described in detail in **4.c**, the PIC has had continued success in enrolling subjects with acute and early HIV-1 infection. However, it is possible that a large, successful public health effort to decrease HIV-1 transmission could reduce HIV-1 incidence and consequently the numbers of subjects with acute and very early HIV-1 infection referred to the PIC. Although Dr. Stekler is working with Public Health - Seattle & King County to implement the recent recommendations issued from the Centers for Disease Control and Prevention (CDC) that advocate HIV-1 testing for all adults and increased frequency of testing for persons at high risk of HIV-1 acquisition [208], it is unclear what effect this is likely to have on HIV-1 transmission [209]. Even if increased HIV-1 testing is successful in reducing HIV-1 transmission, it is actually likely to increase the number of individuals who are identified during acute HIV-1 infection because the expansion of pooled HIV-1 RNA testing [139, 182] is planned to coincide with the widespread implementation of HIV-1 antibody testing.

**5.f.6 Enrollment of epidemiologically-linked clusters**

The ability to extrapolate our results to make conclusions about the prevalence of transmitted HIV-1 drug resistance in Seattle and elsewhere may be limited if a cluster of epidemiologically-linked subjects are enrolled in the PIC Cohort study. Although a detailed history of sexual behavior and risk factors for HIV acquisition is taken from all individuals referred to the PIC Cohort, the anonymous nature of many partnerships precludes identification of all transmission links. However, results of consensus sequencing could be analyzed for phylogenetic linkages [133, 210, 211] in order to identify clusters of new infected subjects whose referrals might not be independent events and could result in overestimation of the prevalence of transmitted HIV-1 drug resistance. In the situation in which no discrete clusters are identified, the phylogenetic linkages of newly infected subjects could be compared against a control population of *pol* gene sequences from chronically infected individuals to analyze the relative similarity of the newly infected subjects.

**5.f.7 Routine use of more-sensitive HIV-1 drug resistance testing assays**

Given the current interest in this topic at recent national meetings, it is possible that more-sensitive HIV-1 drug resistance testing assays may be routinely used as part of clinical care of HIV-infected patients prior to the anticipated completion of this project. If true, we will have less power to detect differences in the clinical outcomes associated with low-frequency HIV-1 drug resistance, as subjects would be less likely to initiate ARV regimens with fewer than three active ARV agents. However, no such assay is FDA-approved, and none are currently being evaluated for commercial development, making such a possibility unlikely.

**5.g Timetable for all projects**

Y1 0-6 months Submission of modification to IRB application

Confirmation of yet-to-be confirmed transmission pairs in PIC Partner Study

Continued enrollment in PIC Cohort and PIC Partners Studies (through Y5)

Y1-2 OLA of source and recipient partner-pairs (Aim #3)

Y2-3 OLA of untreated subjects (Aim #1)

Data analysis and manuscript preparation of Aim #3

Y3-4 OLA of treated subjects (Aim #2)

Data analysis and manuscript preparation of Aim #1

Y4-Y5 Data analysis, manuscript preparation of Aim #2 and future grant submission

**5.h Future studies**

Results of the studies proposed within this application have a high likelihood of leading to future funded multidisciplinary projects between the Seattle Primary Infection Program (SeaPIP) and Public Health - Seattle & King County. Examples of these potential projects include:

**5.h.1 Studies of viral dynamics**

Drs. Sarah Holte and Ann Collier will submit an R01 proposal in parallel with this application to support an intensive viral dynamics study. If both applications are funded, we will integrate the HIV-1 drug resistance testing results provided by this study into the viral dynamics study to understand the relationship between transmitted HIV-1 drug resistance, the rate of viral decay following HIV-1 acquisition, and the viral set point.

**5.h.2 Studies of viral fitness**

Studies evaluating the replicative viral fitness of transmitted HIV-1 drug resistance mutations have not been proposed as part of this application because of their prohibitive costs and the descriptive nature of these analyses. However, our SeaPIP collaborators (Dr. James Mullins and Dr. Eric Arts) have proposed these evaluations in a separate program grant application for a subset of partner-pairs who have previously enrolled in the PIC Cohort and PIC Partner studies. Additional applications could be submitted, for example, to fund studies to determine whether the continued detection of low-frequency viral variants with HIV-1 drug resistance is associated with replicative viral fitness or to perform additional studies including limiting dilution cultures with cryopreserved cells to see if drug resistant viruses represent non-viable virus or grow less well.

**5.h.3 Clinical trials to evaluate the use of assays to detect low-frequency HIV-1 drug resistance**

There is significant confounding inherent in most observational studies, especially those that evaluate the use of ARV therapy in primary HIV-1 infection. If results from this project suggest that transmitted low-frequency HIV-1 drug resistance has a negative impact on the response to ARV therapy, a randomized clinical trial should be performed to evaluate whether use of more-sensitive HIV-1 drug resistance assays to select drug regimens for ARV-naïve individuals is associated with improved clinical outcomes.

**5.h.4 Public health surveillance and mathematical modeling**

Estimates of the transmission probabilities of HIV-1 drug resistance obtained in Aim #3 of this project will be used in mathematical models currently being planned through ongoing collaborations within PHSKC and the UW Center for AIDS and STD. Coupled with PHSKC surveillance efforts, these mathematical models will increase our understanding of the population-level dynamics of transmitted HIV-1 drug resistance and will be instrumental to develop public health interventions to reduce the spread of transmitted HIV-1 drug resistance.

**8. Protection of Human Subjects**

These studies are currently approved under UW HSD # 03-788-B (PI: Ann Collier, MD): The Virologic and Immunologic Events in Acute/Early HIV-1 Infection and UW HSD # 06-1319-D (PI: Joanne Stekler, MD MPH): A Study of Partners of Subjects with Primary HIV-1 Infection. The consent forms for the active protocols are included as Appendices with this application. If this application is funded, amendments to these approvals will be submitted to account for the new funding and the relevant changes to the consents and protocols.

**8.a Risks to the subjects**

**8.a.1 Study population**

The study population for this project will consist of persons with documented HIV-1 infection currently enrolled in the PIC and persons at risk for HIV-1 infection evaluated as potential source partners. We plan to continue follow-up of *70* high priority subjects, with duration of follow-up and visit frequency as specified in the PIC Cohort and PIC Partners studies schedules of evaluations (**Tables 11 and 12**).

Subjects will be both men and women who are at least 16-18 years old (*the* age limit is protocol-dependent). The UW Human Subjects Division grants approval for enrollment of adolescents on a study-by-study basis, depending upon the potential risks versus benefits for the protocol. As described in detail in the research plan, approximately 90% of enrollees will be male, and approximately 80% will be Caucasian. Concerted efforts have been made to enroll women and people of color, as described below in the sections called Inclusion of Women and Inclusion of Minorities. We do not recruit or study subjects from prison populations.

At entry into SeaPIP studies, subjects have/had acute or early HIV-1 infection or are*/were* partners of subjects with acute or early HIV-1. Over the period of follow-up, some subjects may develop progressive HIV-1 infection. Thus, while most subjects will be in an early stage of HIV-1 infection, and be asymptomatic or minimally symptomatic, some may develop symptomatic HIV-1 or AIDS. Subjects will have varying immune status as measured by CD4^+^ T-cell counts, ranging from within normal limits to below normal, depending upon their choices with respect to ARV therapy and the success of that therapy. The health status of participants will also vary depending upon their choice of ARV treatment and its outcome and any concomitant health problems.

**8.a.2 Sources of Materials**

Research materials for this project include clinical data obtained from medical histories, reviews of systems, physical examinations, and standardized questionnaires; laboratory test results from specimens including blood and genital secretions; and selected data from medical records. Most clinical and laboratory data collected as part of the PIC studies will be obtained specifically for research. We may also obtain written permission from subjects to access information from outside providers that is pertinent to these studies.

**8.a.3 Potential Risks**

The major risks of participation in SeaPIP protocols include those related to the study evaluations. The protocol visits are frequent during the first year and require a substantial time commitment. Subjects may experience increased stress secondary to having discussions about HIV-1 infection, risk factors for acquisition of HIV-1 infection, and discussion of past medical and sexual history. This stress is in addition to the psychological stress for new enrollees of learning that they have recently acquired HIV-1 infection. Physical examinations may be uncomfortable. These activities may involve an invasion of privacy. Blood draws can be uncomfortable and cause bruising. Frequent blood draws are required during the first year of enrollment in the PIC and may result in a decrease in red blood count. Other procedures, such as collection of genital secretions may invade subjects' privacy.

Subjects may perceive a loss of confidentiality by having records accessible to the study staff; study monitors, UW regulatory or fiscal oversight staff, or by having to register as a patient at one of the involved institutions, if they have not received care there previously. In addition, subjects may be concerned about the loss of confidentiality if a diagnosis is made (e.g. *syphilis or other bacterial STI*) that is reportable by state regulations to the health department.

## 8.b Adequacy of protection against risks

**8.b.1 Recruitment and informed consent**

PIC subjects have been recruited from several sources, including Public Health - Seattle & King County (including the Sexually Transmitted Disease Clinic at Harborview Medical Center); UW clinics and clinical research programs, including the UW HIV-1 Prevention Trials Unit; community medical providers who provide care for persons with and at risk for HIV-1, and community-based HIV-1 social service organizations. The PIC staff works with a Community Advisory Board to inform the community about the PIC, its protocols and their results. Subjects are recruited only for IRB-approved protocols.

Written informed consent is obtained from all study participants, both for screening and protocol participation. Consent is obtained by a clinical staff member (health care specialist or physician). During the consent process, subjects are presented information verbally as well as in writing. The information provided verbally is similar to that contained in the consent form. The consent form describes the study in detail, including the purpose of each study, the study procedures, duration and frequency of visits, types of information and specimens to be collected, study therapies (if any), the potential risks and benefits of study participation and alternatives to study participation. No relevant information is withheld, unless required by a specific study protocol. Subjects are encouraged to ask questions and to discuss their participation with their personal physician, family or friends before they enter a PIC study. Subjects are provided information at one visit and asked to carefully consider it before returning for another visit, at which time the information is discussed again. The signing of the consent form is witnessed and dated by a PIC staff member (who is a co-investigator for that protocol). Each subject is given a copy of the informed consent document. The consent forms are updated as necessary to reflect protocol revisions or new information. When new consent forms are available and approved by the IRB, study participants are counseled verbally about the changes and given the revised consent form to read before being asked to sign it.

## 8.b.2 Protection against risk

The procedures required for PIC research protocols are standard medical procedures that are carried out by highly qualified and experienced personnel. To protect confidentiality, all specimens and research records are identified only by code numbers, with the records linking the code to subject identifiers kept in a separate locked file. Identifying information (names, addresses, social security numbers, etc.) is excluded from computerized databases, other than one separate clinic-management database with limited access (maintained on a separate computer with limited access, in a password-protected file, which is accessible only to selected staff). The purpose of this local database is to efficiently record and update the data necessary to contact subjects and their medical care providers. To maximize the confidentiality of the data we collect, we also have a Federal Confidentiality Certificate for the PIC cohort (expiration date 3/31/09), *and one has been submitted for the PIC Partners protocol.*

Stress, anxiety, and depression as a result of study participation will be minimized by periodic reassurance, careful explanation of study procedures and results, maintenance of an open, supporting attitude by all project personnel, and referral back to the subjects’ primary care providers for further evaluation and care as needed. Routine clinical tests will be performed in CAP-certified laboratories (Washington State is CLIA-exempt) to ensure accuracy of the results. The potential of anemia from study phlebotomy will be decreased by adhering to eligibility requirements for each protocol, monitoring of hemoglobin/hematocrit, and ensuring adherence to blood-draw volume requirements. A record of all study phlebotomies and blood volumes drawn is maintained for each subject. Immediate care for any medical complications due to the study procedures will be provided by the investigators within their areas of competence. Subjects will be responsible for costs of care if other medical services or hospitalization should be required. In the event that study procedures lead to a clinically significant diagnosis requiring intervention, the subject will be referred back to his or her primary care provider, or the investigators will assist the subject in identifying a source of medical care if they have not been in care (i.e. at a first PIC visit).

All participating staff and faculty at all sites will maintain the confidentiality of PIC subjects. Risks to seronegative personnel of acquisition of HIV-1 through accidental exposure will be minimized by rigorous adherence to the institution’s infection control procedures *including mandatory training in bloodborne pathogens. Laboratory staff receive additional training in appropriate personal protective equipment, and infectious specimens are processed in a BSL 2+ room within a biosafety cabinet.*

Other procedures to minimize risk include careful attention to the recognition and prompt reporting, to the relevant regulatory agencies (e.g. IRB) and sponsor (if applicable) of adverse experiences that occur to participants at the PIC. A UW Adverse Effect Report form is completed for each serious, severe or unexpected, or more severe than expected) adverse experience and sent promptly to the UW IRB. Per the UW IRB’s request, expected adverse events and less serious adverse events are summarized annually for each study rather than being communicated real-time, unless there is something unusual about the pattern or frequency of such adverse events. Additionally, when a staff member learns that an adverse experience which meets reporting requirements has occurred in a study participant, an adverse experience report (AER) is completed and communicated promptly to other relevant individuals or groups. Reports will be made for serious AEs within 1-2 working days of site awareness. Both Adverse Effects or Adverse Event Reports are completed by the sub-investigator managing the subject (physician or health care specialist) and then promptly reviewed and signed by a physician prior to submission. The reports include the nature of the serious adverse experience, an assessment of its relationship to study procedures, and the action taken by the investigators. If needed, follow-up reports are sent with additional details about the event, its evaluation and management, and its resolution.

## 8.c Potential benefits of the proposed research

Subjects will receive extensive education about HIV-1 infection and the PIC enrollees will receive emotional support through their longitudinal relationships with the study staff. The subjects will have laboratory and clinical assessments that may detect serious conditions at an earlier, more treatable stage. Procedures will be cost-free, including numerous tests (e.g. CD4^+^ cell counts, HIV-1 RNA determinations) for which subjects would otherwise have to pay. PIC partners will learn about HIV-1 and methods to prevent its transmission. Substantial societal benefits will accrue from the knowledge gained from this research. Potential benefits include increased understanding of HIV-1 infection, increased knowledge about treatment strategies for primary HIV-1, and increased knowledge about transmission of drug-resistant HIV-1. This knowledge may help improve the health status of persons with HIV-1, may allow subjects to remain functional and employable for longer, may prolong life or improve its quality, may help to develop strategies to prevent transmission of drug-resistant HIV-1 and help HIV vaccine development.

## 8.d Importance of the knowledge to be gained

HIV-1 and AIDS are a threat to the health of millions of persons worldwide. While improvements in the management of HIV-1 have resulted in a larger number of persons living with HIV-1, ARV therapy has potential for significant side-effects and cost, and transmitted HIV-1 drug resistance may limit the successes that we have had to date. There is a need to improve the management strategies for HIV-1, to increase their effectiveness and decrease their toxicity and cost. By increasing our knowledge about transmitted HIV-1 drug resistance, the studies performed in this proposal have the potential to help improve the management and health status of persons with HIV-1 and help develop strategies to limit the spread of transmitted HIV-1 drug resistance.

**8.e Data safety and monitoring plan**

The monitoring plan for these protocols includes annual summaries that detail study enrollment, withdrawals, reason for withdrawal, adverse events, and complaints and how they were handled. These reports are reviewed by the Principal Investigator and IRB to decide if modifications in the research plan are needed.

All subjects are informed promptly of new information, which might affect the risks of study participation, and are asked to sign the revised IRB-approved consent form or addendum consent form. If subjects do not have upcoming visits scheduled in the near future, they are called to inform them about potentially serious information. Otherwise, they receive the information at their next visit. The mechanism to ensure that each subject signs the revised consent form is to place unsigned copies at the front of each subject’s research chart, to remind the staff to inform the subjects about the issue at their next visit. These procedures are designed to ensure that co-investigators, study participants and staff are fully informed, in order to minimize the exposure of subjects to undue risk without their knowledge and consent.

## OHRP Assurance Numbers

The UW IRB, called the Human Subjects Review Committee (HSRC), is the oversight committee for the protocols conducted at the PIC. The UW HSRC Federal Wide Assurance number is FWA00006878.

## 9. Inclusion of Women and Minorities

**9.a Inclusion of Women**

Our protocols enroll both men and women because the issues that we will address are relevant to both genders. However, women and heterosexual men are under-represented in the cohort compared to individuals who are diagnosed with established HIV-1 infection in Seattle & King County. Reasons for this are multi-factorial, including the targeting of pooled HIV-1 RNA testing to men who have sex with men only and the difficulty of identifying women and heterosexual men who present for clinical care with a classic syndrome in a city where the risk of HIV-1 acquisition is predominantly among men who have sex with men. Examples of outreach efforts for women have included publicity efforts among women at risk for HIV-1 (through sexually transmitted disease and community health clinics and community organizations); defraying the cost of childcare for clinic visits; offering early morning and very late afternoon visit times, as well as times during the day, to maximize convenience of visits for potential participants; and defraying transportation costs for subjects who would otherwise be unable to participate. Women of childbearing potential or who are breastfeeding are eligible for inclusion in the cohort.

Unfortunately, identifying and recruiting women with primary HIV-1 infection and even women with chronic HIV-1 infection in our community has been very difficult. Ten percent of the 324 phone referrals to the PIC over the past 5 years have been women, consistent with the area HIV-1 demographics; one third of these women met our standardized criteria for having an in-person screening visit, 3 were diagnosed with primary HIV-1 infection, and 1 enrolled in the PIC. One of the women who declined enrollment had multiple mental health issues that appropriately took priority over research study participation, and one woman agreed to enrollment but failed multiple follow-up visits. Data from PHSKC surveillance corroborates these findings; from 2001-2004, only 6 (0.3%) of 1841 women tested anonymously at PHSKC HIV-1 testing sites had HIV-1 infection. Four of these 6 underwent screening with a less sensitive enzyme immunoassay (LS-EIA) for HIV to determine if any had recently acquired HIV-1 infection; all tests were reactive, suggesting that none had been recently infected. Among 10,721 women tested confidentially by PHSKC during the same time frame, the prevalence of HIV-1 was 0.2%; 14 of the HIV-1 infected women had a LS-EIA and 3 were found to have early HIV-1 infection. Of note, women are not included in the pooled HIV-1 NAAT program because of the low prevalence and incidence of HIV-1 infection in women in our area. These data illustrate the barriers that we have faced in recruiting women to the studies that will provide specimens for this proposal.

## 9.b Inclusion of Minorities

Our protocols enroll eligible persons of all ethnicities and races. Examples of past outreach efforts for PIC studies included extensive publicity efforts among minority men and women at risk for HIV-1 (through sexually transmitted disease and community health clinics and community organizations including the People of Color Against AIDS Network [POCAAN]), minority publications, community health fairs, and via minority health care provider organizations and providers of care to minority men and women). In order to ensure that financial barriers will not prevent continued participation in SeaPIP studies, we also pay for transportation costs for subjects who would otherwise be unable to participate, and reimburse for time spent during study procedures.

**11. Inclusion of children**

Our ongoing PIC Cohort study is approved to enroll subjects age 16 years and older and the PIC partners study is approved to enroll subjects >18 years. The UW IRB has allowed enrollment of adolescents into these protocols since HIV-1 is increasing in adolescents and information about the disease and HIV-1 transmission in this population is relevant. Inclusion of minors allows the possibility of examining whether HIV-1 could behave differently in people of different age groups. Younger children will be referred to the UW Pediatric AIDS Clinical Trials Unit, which exists to conduct research on children and adolescents with HIV-1 infection.

The study physicians and staff have appropriate expertise to evaluate, monitor and manage persons ages 16-21 years, and the facilities available are appropriate for persons ages 16-21 years. The statistical plans have a sufficient total enrollment to address the primary research questions, but not for age-defined subgroups.

**17. Resource Sharing Plan**

Data obtained during this project will be available at the end of the funding cycle or within a year of publication of major findings from the project, whichever is later. Blood and other specimens may be available as well. Requests for data or specimens must be made in writing, via the Primary Infection Cohort specimen request requisition form. This form asks for detailed information including a lay language summary, study design, laboratory and statistical methods. Human subjects approval must be obtained prior to transfer of specimens or data. For specimen requests, a Material Transfer Agreement will be required from non-SeaPIP investigators. Applications must be approved by a majority vote of active SeaPIP collaborators.

Data will be provided electronically in formatted text or Excel files and will be stripped of identifying information prior to data sharing. Investigators receiving data will be asked to sign an agreement stipulating that they will (1) use the data only for not-for-profit research purposes (unless negotiated otherwise with the investigator and the University of Washington); (2) not attempt to identify any individual participant; (3) secure the data using appropriate computer technology and not share the data with anyone else; and (4) destroy the data after analyses are completed.

**7. Bibliography and References Cited**

1. Tobe VO, Taylor SL, Nickerson DA. **Single-well genotyping of diallelic sequence variations by a two-color ELISA-based oligonucleotide ligation assay**. *Nucleic Acids Res* 1996,24:3728-3732 [PMID#: 8871551].

2. Paredes R, Lalama C, Ribaudo H, Schackman B, Shikuma C, Meyer W, 3rd*, et al.* **Presence of Minor Populations of Y181C Mutants Detected by Allele-specific PCRand Risk of Efavirenz Failure in Treatment-naïve Patients: Results of an ACTG 5095 Case-cohort Study** *15th Conference on Retroviruses and Opportunistic Infections; Boston, MA; February 3-6, 2008 [abstract #83]*.

3. Huppler Hullsiek K, Peng G, Simen B, Simons J, Egholm M, Novak R*, et al.* **Virologic Success of Different Strategies for Initial ART Regimens Is Predicted by the Type and Detection Level of Minor Drug-resistant Variant Detected by Ultra Deep Sequencing: The CPCRA 058 FIRST Study**. *15th Conference on Retroviruses and Opportunistic Infections; Boston, MA; February 3-6, 2008 [abstract #878].*

4. Peuchant O, Thiebaut R, Capdepont S, Lavignolle-Aurillac V, Neau D, Morlat P*, et al.* **Transmission of HIV-1 minority-resistant variants and response to first-line antiretroviral therapy**. *AIDS* 2008,22:1417-1423 [PMID#: 18614864].

5. Johnson JA, Li JF, Wei X, Lipscomb J, Irlbeck D, Craig C*, et al.* **Minority HIV-1 drug resistance mutations are present in antiretroviral treatment-naive populations and associate with reduced treatment efficacy**. *PLoS Med* 2008,5:e158 [PMID#: 18666824].

6. Giulieri S, Knoepfel S, Rauch P, Gunthard HF, Cavassini M, Metzner KJ. **Minority quasispecies of drug-resistant HIV-1 leading to early therapy failure in treatment-naive and adherent patients**. *XII International HIV Drug Resistance Workshop; Sitges, Spain; June 10-14, 2008 [abstract #106]*.

7. Deeks SG. **Transmitted minority drug-resistant HIV variants: a new epidemic?** *PLoS Med* 2008,5:e164 [PMID#: 18666826].

8. **U.S. Department of Health and Human Services. Guidelines for the Use of Antiretroviral Agents in HIV-1-Infected Adults and Adolescents, January 29, 2008; accessed August 1, 2008.** available at http://aidsinfo.nih.gov/contentfiles/adultandadolescentgl.pdf: [PMID#:

9. Little SJ, Frost SD, Wong JK, Smith DM, Pond SL, Ignacio CC*, et al.* **Persistence of transmitted drug resistance among subjects with primary human immunodeficiency virus infection**. *J Virol* 2008,82:5510-5518 [PMID#: 18353964].

10. Buskin SE, Kahle EM, Horwitch C, Pergam SA, Ellis GM, Frenkel LM*, et al.* **HIV-1 drug resistance surveillance in Seattle, WA including multi-class drug resistance and a cluster of four individuals with highly-related highly resistant virus**. *17th ISSTDR and 10th IUSTI; Seattle, WA; July 29-August 1, 2007 [abstract #O-093]*.

11. Larder BA, Kohli A, Kellam P, Kemp SD, Kronick M, Henfrey RD. **Quantitative detection of HIV-1 drug resistance mutations by automated DNA sequencing**. *Nature* 1993,365:671-673 [PMID#: 8413632].

12. Leitner T, Halapi E, Scarlatti G, Rossi P, Albert J, Fenyo EM, Uhlen M. **Analysis of heterogeneous viral populations by direct DNA sequencing**. *Biotechniques* 1993,15:120-127 [PMID#: 8363827].

13. Schuurman R, Demeter L, Reichelderfer P, Tijnagel J, de Groot T, Boucher C. **Worldwide evaluation of DNA sequencing approaches for identification of drug resistance mutations in the human immunodeficiency virus type 1 reverse transcriptase**. *J Clin Microbiol* 1999,37:2291-2296 [PMID#: 10364600].

14. Van Laethem K, Van Vaerenbergh K, Schmit JC, Sprecher S, Hermans P, De Vroey V*, et al.* **Phenotypic assays and sequencing are less sensitive than point mutation assays for detection of resistance in mixed HIV-1 genotypic populations**. *J Acquir Immune Defic Syndr* 1999,22:107-118 [PMID#: 10843523].

15. Schuurman R, Brambilla D, de Groot T, Huang D, Land S, Bremer J*, et al.* **Underestimation of HIV type 1 drug resistance mutations: results from the ENVA-2 genotyping proficiency program**. *AIDS Res Hum Retroviruses* 2002,18:243-248 [PMID#: 11860670].

16. Palmer S, Kearney M, Maldarelli F, Halvas EK, Bixby CJ, Bazmi H*, et al.* **Multiple, linked human immunodeficiency virus type 1 drug resistance mutations in treatment-experienced patients are missed by standard genotype analysis**. *J Clin Microbiol* 2005,43:406-413 [PMID#: 15635002].

17. Chan KC, Galli RA, Montaner JS, Harrigan PR. **Prolonged retention of drug resistance mutations and rapid disease progression in the absence of therapy after primary HIV infection**. *AIDS* 2003,17:1256-1258 [PMID#: 12819530].

18. Brenner B, Routy JP, Quan Y, Moisi D, Oliveira M, Turner D, Wainberg MA. **Persistence of multidrug-resistant HIV-1 in primary infection leading to superinfection**. *AIDS* 2004,18:1653-1660 [PMID#: 15280776].

19. Delaugerre C, Morand-Joubert L, Chaix ML, Picard O, Marcelin AG, Schneider V*, et al.* **Persistence of multidrug-resistant HIV-1 without antiretroviral treatment 2 years after sexual transmission**. *Antivir Ther* 2004,9:415-421 [PMID#: 15259904].

20. Pao D, Andrady U, Clarke J, Dean G, Drake S, Fisher M*, et al.* **Long-term persistence of primary genotypic resistance after HIV-1 seroconversion**. *J Acquir Immune Defic Syndr* 2004,37:1570-1573 [PMID#: 15577410].

21. Brenner BG, Routy JP, Petrella M, Moisi D, Oliveira M, Detorio M*, et al.* **Persistence and fitness of multidrug-resistant human immunodeficiency virus type 1 acquired in primary infection**. *J Virol* 2002,76:1753-1761 [PMID#: 11799170].

22. Neifer S, Somogyi S, Schlote F, Berg T, Poggensee G, Kuecherer C. **Persistence of a sexually transmitted highly resistant HIV-1: pol quasispecies evolution over 33 months in the absence of treatment**. *AIDS* 2006,20:2231-2233 [PMID#: 17086065].

23. Ghosn J, Pellegrin I, Goujard C, Deveau C, Viard JP, Galimand J*, et al.* **HIV-1 resistant strains acquired at the time of primary infection massively fuel the cellular reservoir and persist for lengthy periods of time**. *AIDS* 2006,20:159-170 [PMID#: 16511408].

24. Barbour JD, Hecht FM, Wrin T, Liegler TJ, Ramstead CA, Busch MP*, et al.* **Persistence of primary drug resistance among recently HIV-1 infected adults**. *AIDS* 2004,18:1683-1689 [PMID#: 15280779].

25. Smith DM, Wong JK, Shao H, Hightower GK, Mai SH, Moreno JM*, et al.* **Long-Term Persistence of Transmitted HIV Drug Resistance in Male Genital Tract Secretions: Implications for Secondary Transmission**. *J Infect Dis* 2007,196:356-360 [PMID#: 17597449].

26. Zhu T, Wang N, Carr A, Nam DS, Moor-Jankowski R, Cooper DA, Ho DD. **Genetic characterization of human immunodeficiency virus type 1 in blood and genital secretions: evidence for viral compartmentalization and selection during sexual transmission**. *J Virol* 1996,70:3098-3107 [PMID#: 8627789].

27. Bon I, Gibellini D, Borderi M, Alessandrini F, Vitone F, Schiavone P, Re MC. **Genotypic resistance in plasma and peripheral blood lymphocytes in a group of naive HIV-1 patients**. *J Clin Virol* 2007,38:313-320 [PMID#: 17306618].

28. Van Laethem K, De Munter P, Schrooten Y, Verbesselt R, Van Ranst M, Van Wijngaerden E, Vandamme AM. **No response to first-line tenofovir+lamivudine+efavirenz despite optimization according to baseline resistance testing: Impact of resistant minority variants on efficacy of low genetic barrier drugs**. *J Clin Virol* 2007: [PMID#: 17369083].

29. Mellors J, Palmer S, Nissley D, Kearney M, Halvas E, Bixby C*, et al.* **Low frequency NNRTI-resistant variants contribute to failure of efavirenz-containing regimens**. *11th Conference on Retroviruses and Opportunistic Infections; San Francisco, CA; February 8-11, 2004 [abstract #39]*.

30. Izopet J, Cazabat M, Pasquier C, Sandres-Saune K, Bonnet E, Marchou B*, et al.* **Evolution of total and integrated HIV-1 DNA and change in DNA sequences in patients with sustained plasma virus suppression**. *Virology* 2002,302:393-404 [PMID#: 12441083].

31. Pariente N, Pernas M, de la Rosa R, Gomez-Mariano G, Fernandez G, Rubio A*, et al.* **Long-term suppression of plasma viremia with highly active antiretroviral therapy despite virus evolution and very limited selection of drug-resistant genotypes**. *J Med Virol* 2004,73:350-361 [PMID#: 15170628].

32. Riva E, Pistello M, Narciso P, D'Offizi G, Isola P, Galati V*, et al.* **Decay of HIV type 1 DNA and development of drug-resistant mutants in patients with primary HIV type 1 infection receiving highly active antiretroviral therapy**. *AIDS Res Hum Retroviruses* 2001,17:1599-1604 [PMID#: 11779347].

33. Metzner KJ, Allers K, Rauch P, Harrer T. **Rapid selection of drug-resistant HIV-1 during the first months of suppressive ART in treatment-naive patients**. *AIDS* 2007,21:703-711 [PMID#: 17413691].

34. Frenkel LM, Wang Y, Learn GH, McKernan JL, Ellis GM, Mohan KM*, et al.* **Multiple viral genetic analyses detect low-level human immunodeficiency virus type 1 replication during effective highly active antiretroviral therapy**. *J Virol* 2003,77:5721-5730 [PMID#: 12719565].

35. Leigh Brown AJ, Frost SD, Mathews WC, Dawson K, Hellmann NS, Daar ES*, et al.* **Transmission fitness of drug-resistant human immunodeficiency virus and the prevalence of resistance in the antiretroviral-treated population**. *J Infect Dis* 2003,187:683-686 [PMID#: 12599087].

36. de Mendoza C, Rodriguez C, Corral A, del Romero J, Gallego O, Soriano V. **Evidence for differences in the sexual transmission efficiency of HIV strains with distinct drug resistance genotypes**. *Clin Infect Dis* 2004,39:1231-1238 [PMID#: 15486849].

37. VandeVijver D, Wensing A, Asjo B, Bruckova M, Jorgensen LB, Horban A*, et al.* **Selective transmission of drug resistance mutations**. *14th International HIV Drug Resistance Workshop*. Quebec City, Canada. June 7-11, 2005 [abstract #113].

38. Yerly S, Jost S, Telenti A, Flepp M, Kaiser L, Chave JP*, et al.* **Infrequent transmission of HIV-1 drug-resistant variants**. *Antivir Ther* 2004,9:375-384 [PMID#: 15259900].

39. Blick G, Kagan RM, Coakley E, Petropoulos C, Maroldo L, Greiger-Zanlungo P*, et al.* **The Probable Source of Both the Primary Multidrug-Resistant (MDR) HIV-1 Strain Found in a Patient with Rapid Progression to AIDS and a Second Recombinant MDR Strain Found in a Chronically HIV-1-Infected Patient**. *J Infect Dis* 2007,195:1250-1259 [PMID#: 17396993].

40. Wahlberg J, Fiore J, Angarano G, Uhlen M, Albert J. **Apparent selection against transmission of zidovudine-resistant human immunodeficiency virus type 1 variants**. *J Infect Dis* 1994,169:611-614 [PMID#: 8158034].

41. Fiebig EW, Wright DJ, Rawal BD, Garrett PE, Schumacher RT, Peddada L*, et al.* **Dynamics of HIV viremia and antibody seroconversion in plasma donors: implications for diagnosis and staging of primary HIV infection**. *AIDS* 2003,17:1871-1879 [PMID#: 12960819].

42. Janssen RS, Satten GA, Stramer SL, Rawal BD, O'Brien TR, Weiblen BJ*, et al.* **New testing strategy to detect early HIV-1 infection for use in incidence estimates and for clinical and prevention purposes**. *JAMA* 1998,280:42-48 [PMID#: 9660362].

43. Kothe D, Byers RH, Caudill SP, Satten GA, Janssen RS, Hannon WH, Mei JV. **Performance characteristics of a new less sensitive HIV-1 enzyme immunoassay for use in estimating HIV seroincidence**. *J Acquir Immune Defic Syndr* 2003,33:625-634 [PMID#: 12902808].

44. Stekler J, Coombs RW. **Transmitted HIV-1 Drug Resistance: Are We Seeing Just the Tip of an Epidemiological Iceberg?** *J Infect Dis* 2007,196:336-338 [PMID#: 17597446].

45. Little SJ, Daar ES, D'Aquila RT, Keiser PH, Connick E, Whitcomb JM*, et al.* **Reduced antiretroviral drug susceptibility among patients with primary HIV infection**. *JAMA* 1999,282:1142-1149 [PMID#: 10501117].

46. Hecht FM, Grant RM, Petropoulos CJ, Dillon B, Chesney MA, Tian H*, et al.* **Sexual transmission of an HIV-1 variant resistant to multiple reverse-transcriptase and protease inhibitors**. *N Engl J Med* 1998,339:307-311 [PMID#: 9682043].

47. Yerly S, Kaiser L, Race E, Bru JP, Clavel F, Perrin L. **Transmission of antiretroviral-drug-resistant HIV-1 variants**. *Lancet* 1999,354:729-733 [PMID#: 10475184].

48. Salomon H, Wainberg MA, Brenner B, Quan Y, Rouleau D, Cote P*, et al.* **Prevalence of HIV-1 resistant to antiretroviral drugs in 81 individuals newly infected by sexual contact or injecting drug use. Investigators of the Quebec Primary Infection Study**. *AIDS* 2000,14:F17-23 [PMID#: 10708278].

49. Boden D, Hurley A, Zhang L, Cao Y, Guo Y, Jones E*, et al.* **HIV-1 drug resistance in newly infected individuals**. *JAMA* 1999,282:1135-1141 [PMID#: 10501116].

50. Tamalet C, Pasquier C, Yahi N, Colson P, Poizot-Martin I, Lepeu G*, et al.* **Prevalence of drug resistant mutants and virological response to combination therapy in patients with primary HIV-1 infection**. *J Med Virol* 2000,61:181-186 [PMID#: 10797372].

51. **Analysis of prevalence of HIV-1 drug resistance in primary infections in the United Kingdom**. *BMJ* 2001,322:1087-1088 [PMID#: 11337435].

52. Grant RM, Hecht FM, Warmerdam M, Liu L, Liegler T, Petropoulos CJ*, et al.* **Time trends in primary HIV-1 drug resistance among recently infected persons**. *JAMA* 2002,288:181-188 [PMID#: 12095382].

53. Little SJ, Holte S, Routy JP, Daar ES, Markowitz M, Collier AC*, et al.* **Antiretroviral-drug resistance among patients recently infected with HIV**. *N Engl J Med* 2002,347:385-394 [PMID#: 12167680].

54. Chaix ML, Descamps D, Harzic M, Schneider V, Deveau C, Tamalet C*, et al.* **Stable prevalence of genotypic drug resistance mutations but increase in non-B virus among patients with primary HIV-1 infection in France**. *AIDS* 2003,17:2635-2643 [PMID#: 14685058].

55. Ammaranond P, Cunningham P, Oelrichs R, Suzuki K, Harris C, Leas L*, et al.* **No increase in protease resistance and a decrease in reverse transcriptase resistance mutations in primary HIV-1 infection: 1992-2001**. *AIDS* 2003,17:264-267 [PMID#: 12545090].

56. Shet A, Berry L, Mohri H, Mehandru S, Chung C, Kim A*, et al.* **Tracking the prevalence of transmitted antiretroviral drug-resistant HIV-1: a decade of experience**. *J Acquir Immune Defic Syndr* 2006,41:439-446 [PMID#: 16652051].

57. **Investigation of a new diagnosis of multidrug-resistant, dual-tropic HIV-1 infection--New York City, 2005**. *MMWR Morb Mortal Wkly Rep* 2006,55:793-796 [PMID#: 16874293].

58. Metzner KJ, Rauch P, Walter H, Boesecke C, Zollner B, Jessen H*, et al.* **Detection of minor populations of drug-resistant HIV-1 in acute seroconverters**. *AIDS* 2005,19:1819-1825 [PMID#: 16227789].

59. Johnson J, Li J-F, Brant A, Bennett D, Cong M, Spira T*, et al.* **Multi-drug resistant HIV-1 are transmitted more frequently than current estimates**. *14th International HIV Drug Resistance Workshop*. Quebec City, Canada. June 7-11, 2005 [abstract #111].

60. Turriziani O, Bucci M, Stano A, Scagnolari C, Bellomi F, Fimiani C*, et al.* **Genotypic Resistance of Archived and Circulating Viral Strains in the Blood of Treated HIV-Infected Individuals**. *J Acquir Immune Defic Syndr* 2007,44:518-524 [PMID#: 17224845].

61. Eron JJ, Vernazza PL, Johnston DM, Seillier-Moiseiwitsch F, Alcorn TM, Fiscus SA, Cohen MS. **Resistance of HIV-1 to antiretroviral agents in blood and seminal plasma: implications for transmission**. *AIDS* 1998,12:F181-189 [PMID#: 9814860].

62. Lafeuillade A, Solas C, Chadapaud S, Hittinger G, Poggi C, Lacarelle B. **HIV-1 RNA levels, resistance, and drug diffusion in semen versus blood in patients receiving a lopinavir-containing regimen**. *J Acquir Immune Defic Syndr* 2003,32:462-464 [PMID#: 12640207].

63. Zhang H, Dornadula G, Beumont M, Livornese L, Jr., Van Uitert B, Henning K, Pomerantz RJ. **Human immunodeficiency virus type 1 in the semen of men receiving highly active antiretroviral therapy**. *N Engl J Med* 1998,339:1803-1809 [PMID#:

64. Kemal KS, Burger H, Mayers D, Anastos K, Foley B, Kitchen C*, et al.* **HIV-1 drug resistance in variants from the female genital tract and plasma**. *J Infect Dis* 2007,195:535-545 [PMID#: 17230413].

65. Newstein M, Losikoff P, Caliendo A, Ingersoll J, Kurpewski J, Hanley D*, et al.* **Prevalence and persistence of nonnucleoside reverse transcriptase inhibitor mutations in the female genital tract**. *J Acquir Immune Defic Syndr* 2005,38:364-366 [PMID#: 15735459].

66. Schacker T, Little S, Connick E, Gebhard-Mitchell K, Zhang ZQ, Krieger J*, et al.* **Rapid accumulation of human immunodeficiency virus (HIV) in lymphatic tissue reservoirs during acute and early HIV infection: implications for timing of antiretroviral therapy**. *J Infect Dis* 2000,181:354-357 [PMID#: 10608788].

67. Pilcher CD, Shugars DC, Fiscus SA, Miller WC, Menezes P, Giner J*, et al.* **HIV in body fluids during primary HIV infection: implications for pathogenesis, treatment and public health**. *AIDS* 2001,15:837-845 [PMID#: 11399956].

68. Parisi SG, Mazzi R, Boldrin C, Dal Bello F, Franchin E, Andreoni M, Palu G. **Drug-resistance mutations can be archived very early in HIV primary infection**. *AIDS* 2006,20:1337-1338 [PMID#: 16816567].

69. Parisi SG, Boldrin C, Cruciani M, Nicolini G, Cerbaro I, Manfrin V*, et al.* **Both human immunodeficiency virus cellular DNA sequencing and plasma RNA sequencing are useful for detection of drug resistance mutations in blood samples from antiretroviral-drug-naive patients**. *J Clin Microbiol* 2007,45:1783-1788 [PMID#: 17442799].

70. Vicenti I, Razzolini F, Saladini F, Romano L, Zazzi M. **Use of peripheral blood DNA for genotype antiretroviral resistance testing in drug-naive HIV-infected subjects**. *Clin Infect Dis* 2007,44:1657-1661 [PMID#: 17516412].

71. Zhu T, Mo H, Wang N, Nam DS, Cao Y, Koup RA, Ho DD. **Genotypic and phenotypic characterization of HIV-1 patients with primary infection**. *Science* 1993,261:1179-1181 [PMID#: 8356453].

72. Wolfs TF, Zwart G, Bakker M, Goudsmit J. **HIV-1 genomic RNA diversification following sexual and parenteral virus transmission**. *Virology* 1992,189:103-110 [PMID#: 1376536].

73. Delwart E, Magierowska M, Royz M, Foley B, Peddada L, Smith R*, et al.* **Homogeneous quasispecies in 16 out of 17 individuals during very early HIV-1 primary infection**. *AIDS* 2002,16:189-195 [PMID#: 11807302].

74. Learn GH, Muthui D, Brodie SJ, Zhu T, Diem K, Mullins JI, Corey L. **Virus population homogenization following acute human immunodeficiency virus type 1 infection**. *J Virol* 2002,76:11953-11959 [PMID#: 12414937].

75. Sagar M, Lavreys L, Baeten JM, Richardson BA, Mandaliya K, Chohan BH*, et al.* **Infection with multiple human immunodeficiency virus type 1 variants is associated with faster disease progression**. *J Virol* 2003,77:12921-12926 [PMID#: 14610215].

76. Ritola K, Pilcher CD, Fiscus SA, Hoffman NG, Nelson JA, Kitrinos KM*, et al.* **Multiple V1/V2 env variants are frequently present during primary infection with human immunodeficiency virus type 1**. *J Virol* 2004,78:11208-11218 [PMID#: 15452240].

77. Sagar M, Kirkegaard E, Long EM, Celum C, Buchbinder S, Daar ES, Overbaugh J. **Human immunodeficiency virus type 1 (HIV-1) diversity at time of infection is not restricted to certain risk groups or specific HIV-1 subtypes**. *J Virol* 2004,78:7279-7283 [PMID#: 15194805].

78. Gandhi RT, Wurcel A, Rosenberg ES, Johnston MN, Hellmann N, Bates M*, et al.* **Progressive reversion of human immunodeficiency virus type 1 resistance mutations in vivo after transmission of a multiply drug-resistant virus**. *Clin Infect Dis* 2003,37:1693-1698 [PMID#: 14689353].

79. Deeks SG, Wrin T, Liegler T, Hoh R, Hayden M, Barbour JD*, et al.* **Virologic and immunologic consequences of discontinuing combination antiretroviral-drug therapy in HIV-infected patients with detectable viremia**. *N Engl J Med* 2001,344:472-480 [PMID#: 11172188].

80. Parienti JJ, Massari V, Descamps D, Vabret A, Bouvet E, Larouze B, Verdon R. **Predictors of virologic failure and resistance in HIV-infected patients treated with nevirapine- or efavirenz-based antiretroviral therapy**. *Clin Infect Dis* 2004,38:1311-1316 [PMID#: 15127346].

81. McNabb J, Ross JW, Abriola K, Turley C, Nightingale CH, Nicolau DP. **Adherence to highly active antiretroviral therapy predicts virologic outcome at an inner-city human immunodeficiency virus clinic**. *Clin Infect Dis* 2001,33:700-705 [PMID#: 11486292].

82. Le Moing V, Peytavin G, Journot V, Cottalorda J, Bouvet E, Chene G*, et al.* **Plasma levels of indinavir and nelfinavir at time of virologic response may have a different impact on the risk of further virologic failure in HIV-infected patients**. *J Acquir Immune Defic Syndr* 2003,34:497-499 [PMID#: 14657760].

83. Wu H, Huang Y, Acosta EP, Rosenkranz SL, Kuritzkes DR, Eron JJ*, et al.* **Modeling long-term HIV dynamics and antiretroviral response: effects of drug potency, pharmacokinetics, adherence, and drug resistance**. *J Acquir Immune Defic Syndr* 2005,39:272-283 [PMID#: 15980686].

84. Wood E, Hogg RS, Yip B, Moore D, Harrigan PR, Montaner JS. **Superior virological response to boosted protease inhibitor-based highly active antiretroviral therapy in an observational treatment programme**. *HIV Med* 2007,8:80-85 [PMID#: 17352763].

85. Wong JK, Gunthard HF, Havlir DV, Zhang ZQ, Haase AT, Ignacio CC*, et al.* **Reduction of HIV-1 in blood and lymph nodes following potent antiretroviral therapy and the virologic correlates of treatment failure**. *Proc Natl Acad Sci U S A* 1997,94:12574-12579 [PMID#: 9356491].

86. Panidou ET, Trikalinos TA, Ioannidis JP. **Limited benefit of antiretroviral resistance testing in treatment-experienced patients: a meta-analysis**. *AIDS* 2004,18:2153-2161 [PMID#: 15577648].

87. Wegner SA, Wallace MR, Aronson NE, Tasker SA, Blazes DL, Tamminga C*, et al.* **Long-term efficacy of routine access to antiretroviral-resistance testing in HIV type 1-infected patients: results of the clinical efficacy of resistance testing trial**. *Clin Infect Dis* 2004,38:723-730 [PMID#: 14986258].

88. Markowitz M, Mohri H, Mehandru S, Shet A, Berry L, Kalyanaraman R*, et al.* **Infection with multidrug resistant, dual-tropic HIV-1 and rapid progression to AIDS: a case report**. *Lancet* 2005,365:1031-1038 [PMID#: 15781098].

89. Pillay D, Bhaskaran K, Jurriaans S, Prins M, Masquelier B, Dabis F*, et al.* **The impact of transmitted drug resistance on the natural history of HIV infection and response to first-line therapy**. *AIDS* 2006,20:21-28 [PMID#: 16327315].

90. Bhaskaran K, Pillay D, Walker AS, Fisher M, Hawkins D, Gilson R*, et al.* **Do patients who are infected with drug-resistant HIV have a different CD4 cell decline after seroconversion? An exploratory analysis in the UK Register of HIV Seroconverters**. *AIDS* 2004,18:1471-1473 [PMID#: 15199326].

91. Little SJ, Grant RM, Daar ES, Markowitz M, Hecht FM, Johnson V*, et al.* **Transmitted NNRTI drug resistance is associated with higher steady state viral load measures in untreated subjects with primary HIV infection**. *XIII International HIV Drug Resistance Workshop; Tenerife Sur-Costa Adeje, Canary Islands Spain; June 8-12, 2004 [abstract #49]*.

92. Derdelinckx I, Van Laethem K, Maes B, Schrooten Y, De Wit S, Florence E*, et al.* **Current levels of drug resistance among therapy-naive HIV-infected patients have significant impact on treatment response**. *J Acquir Immune Defic Syndr* 2004,37:1664-1666 [PMID#: 15577426].

93. Fox J, Dustan S, McClure M, Weber J, Fidler S. **Transmitted drug-resistant HIV-1 in primary HIV-1 infection; incidence, evolution and impact on response to antiretroviral therapy**. *HIV Med* 2006,7:477-483 [PMID#: 16925735].

94. Borroto-Esoda K, Harris J, Waters J, Hinkle J, Shaw A, Quinn J, Rousseau F. **Baseline genotype as a predictor of virologic failure in patients receiving Emtricitabine (FTC) once daily or Stavudine (d4T) twice daily in combination with Didanosine (ddI) and Efavirenz (EFV)**. *11th Conference on Retroviruses and Opportunistic Infections; February 8-11, 2004; San Francisco, CA [abstract #672]*.

95. Little S, Frost S, Smith D, May S, Parkin N, Richman D. **Transmission of HIV drug resistance and treatment response**. *14th Conference on Retroviruses and Opportunistic Infections; Los Angeles, CA; February 25-28, 2007 [abstract #60]*.

96. Poggensee G, Kucherer C, Werning J, Somogyi S, Bieniek B, Dupke S*, et al.* **Impact of transmission of drug-resistant HIV on the course of infection and the treatment success. Data from the German HIV-1 Seroconverter Study**. *HIV Med* 2007,8:511-519 [PMID#: 17944684].

97. Kuritzkes DR, Lalama CM, Ribaudo HJ, Marcial M, Meyer WA, 3rd, Shikuma C*, et al.* **Preexisting resistance to nonnucleoside reverse-transcriptase inhibitors predicts virologic failure of an efavirenz-based regimen in treatment-naive HIV-1-infected subjects**. *J Infect Dis* 2008,197:867-870 [PMID#: 18269317].

98. Bannister WP, Cozzi-Lepri A, Clotet B, Mocroft A, Kjaer J, Reiss P*, et al.* **Transmitted drug resistant HIV-1 and association with virologic and CD4 cell count response to combination antiretroviral therapy in the EuroSIDA Study**. *J Acquir Immune Defic Syndr* 2008,48:324-333 [PMID#: 18545152].

99. Berrey MM, Schacker T, Collier AC, Shea T, Brodie SJ, Mayers D*, et al.* **Treatment of primary human immunodeficiency virus type 1 infection with potent antiretroviral therapy reduces frequency of rapid progression to AIDS**. *J Infect Dis* 2001,183:1466-1475 [PMID#: 11319682].

100. Smith D, Berrey MM, Robertson M, Mehrotra D, Markowitz M, Perrin L*, et al.* **Virological and immunological effects of combination antiretroviral therapy with zidovudine, lamivudine, and indinavir during primary human immunodeficiency virus type 1 infection**. *J Infect Dis* 2000,182:950-954 [PMID#: 10950796].

101. Lafeuillade A, Poggi C, Tamalet C, Profizi N, Tourres C, Costes O. **Effects of a combination of zidovudine, didanosine, and lamivudine on primary human immunodeficiency virus type 1 infection**. *J Infect Dis* 1997,175:1051-1055 [PMID#: 9129065].

102. Markowitz M. **Acute HIV-1 infection: early identification and treatment**. *Am Fam Physician* 1999,60:413-414, 417 [PMID#: 10465218].

103. Fidler S, Fraser C, Fox J, Tamm N, Griffin JT, Weber J. **Comparative potency of three antiretroviral therapy regimes in primary HIV infection**. *AIDS* 2006,20:247-252 [PMID#: 16511418].

104. Streeck H, Jessen H, Alter G, Teigen N, Waring MT, Jessen A*, et al.* **Immunological and Virological Impact of Highly Active Antiretroviral Therapy Initiated during Acute HIV-1 Infection**. *J Infect Dis* 2006,194:734-739 [PMID#: 16941338].

105. Kaufmann GR, Zaunders JJ, Cunningham P, Kelleher AD, Grey P, Smith D*, et al.* **Rapid restoration of CD4 T cell subsets in subjects receiving antiretroviral therapy during primary HIV-1 infection**. *AIDS* 2000,14:2643-2651 [PMID#: 11125882].

106. Furtado MR, Callaway DS, Phair JP, Kunstman KJ, Stanton JL, Macken CA*, et al.* **Persistence of HIV-1 transcription in peripheral-blood mononuclear cells in patients receiving potent antiretroviral therapy**. *N Engl J Med* 1999,340:1614-1622 [PMID#: 10341273].

107. Hecht FM, Wang L, Collier A, Little S, Markowitz M, Margolick J*, et al.* **A Multicenter Observational Study of the Potential Benefits of Initiating Combination Antiretroviral Therapy during Acute HIV Infection**. *J Infect Dis* 2006,194:725-733 [PMID#: 16941337].

108. Schacker TW, Hughes JP, Shea T, Coombs RW, Corey L. **Biological and virologic characteristics of primary HIV infection**. *Ann Intern Med* 1998,128:613-620 [PMID#: 9537934].

109. Lockman S, Shapiro RL, Smeaton LM, Wester C, Thior I, Stevens L*, et al.* **Response to antiretroviral therapy after a single, peripartum dose of nevirapine**. *N Engl J Med* 2007,356:135-147 [PMID#: 17215531].

110. Flys TS, Donnell D, Mwatha A, Nakabiito C, Musoke P, Mmiro F*, et al.* **Persistence of K103N-containing HIV-1 variants after single-dose nevirapine for prevention of HIV-1 mother-to-child transmission**. *J Infect Dis* 2007,195:711-715 [PMID#: 17262714].

111. Flys T, Nissley DV, Claasen CW, Jones D, Shi C, Guay LA*, et al.* **Sensitive drug-resistance assays reveal long-term persistence of HIV-1 variants with the K103N nevirapine (NVP) resistance mutation in some women and infants after the administration of single-dose NVP: HIVNET 012**. *J Infect Dis* 2005,192:24-29 [PMID#: 15942890].

112. Loubser S, Balfe P, Sherman G, Hammer S, Kuhn L, Morris L. **Decay of K103N mutants in cellular DNA and plasma RNA after single-dose nevirapine to reduce mother-to-child HIV transmission**. *AIDS* 2006,20:995-1002 [PMID#: 16603851].

113. Simon V, Padte N, Murray D, Vanderhoeven J, Wrin T, Parkin N*, et al.* **Infectivity and replication capacity of drug-resistant human immunodeficiency virus type 1 variants isolated during primary infection**. *J Virol* 2003,77:7736-7745 [PMID#: 12829813].

114. Mammano F, Petit C, Clavel F. **Resistance-associated loss of viral fitness in human immunodeficiency virus type 1: phenotypic analysis of protease and gag coevolution in protease inhibitor-treated patients**. *J Virol* 1998,72:7632-7637 [PMID#: 9696866].

115. Stoddart CA, Liegler TJ, Mammano F, Linquist-Stepps VD, Hayden MS, Deeks SG*, et al.* **Impaired replication of protease inhibitor-resistant HIV-1 in human thymus**. *Nat Med* 2001,7:712-718 [PMID#: 11385509].

116. Miller CJ, Marthas M, Greenier J, Lu D, Dailey PJ, Lu Y. **In vivo replication capacity rather than in vitro macrophage tropism predicts efficiency of vaginal transmission of simian immunodeficiency virus or simian/human immunodeficiency virus in rhesus macaques**. *J Virol* 1998,72:3248-3258 [PMID#: 9525652].

117. Eshleman SH, Lie Y, Hoover DR, Chen S, Hudelson SE, Fiscus SA*, et al.* **Association between the replication capacity and mother-to-child transmission of HIV-1, in antiretroviral drug-naive Malawian women**. *J Infect Dis* 2006,193:1512-1515 [PMID#: 16652278].

118. Cong ME, Heneine W, Garcia-Lerma JG. **The fitness cost of mutations associated with human immunodeficiency virus type 1 drug resistance is modulated by mutational interactions**. *J Virol* 2007,81:3037-3041 [PMID#: 17192300].

119. Bailey JR, O'Connell K, Yang HC, Han Y, Xu J, Jilek B*, et al.* **Transmission of human immunodeficiency virus type 1 from a patient who developed AIDS to an elite suppressor**. *J Virol* 2008,82:7395-7410 [PMID#: 18495769].

120. Dalod M, Salmon-Ceron D, Tachet A, Dulioust E, DeAlmeida M, Finkelsztejn L*, et al.* **Virological and Immunological Characteristics of Couples Serodifferent for Human Immunodeficiency Virus Serostatus**. *37th ICAAC*, September 28-October 1, 1997 1997.

121. Chakraborty H, Sen PK, Helms RW, Vernazza PL, Fiscus SA, Eron JJ*, et al.* **Viral burden in genital secretions determines male-to-female sexual transmission of HIV-1: a probabilistic empiric model**. *AIDS* 2001,15:621-627 [PMID#: 11317000].

122. Quinn TC, Wawer MJ, Sewankambo N, Serwadda D, Li C, Wabwire-Mangen F*, et al.* **Viral load and heterosexual transmission of human immunodeficiency virus type 1. Rakai Project Study Group**. *N Engl J Med* 2000,342:921-929 [PMID#: 10738050].

123. Butler DM, Smith DM, Cachay ER, Hightower GK, Nugent CT, Richman DD, Little SJ. **Herpes simplex virus 2 serostatus and viral loads of HIV-1 in blood and semen as risk factors for HIV transmission among men who have sex with men**. *AIDS* 2008,22:1667-1671 [PMID#: 18670228].

124. Stekler J, Sycks B, Holte S, Maenza J, Stevens C, Dragavon J*, et al.* **HIV dynamics in seminal plasma during primary HIV infection**. *AIDS Research and Human Retroviruses* 2008, in press: [PMID#:

125. Pilcher CD, Joaki G, Hoffman IF, Martinson FE, Mapanje C, Stewart PW*, et al.* **Amplified transmission of HIV-1: comparison of HIV-1 concentrations in semen and blood during acute and chronic infection**. *AIDS* 2007,21:1723-1730 [PMID#: 17690570].

126. Wawer MJ, Gray RH, Sewankambo NK, Serwadda D, Li X, Laeyendecker O*, et al.* **Rates of HIV-1 transmission per coital act, by stage of HIV-1 infection, in Rakai, Uganda**. *J Infect Dis* 2005,191:1403-1409 [PMID#: 15809897].

127. Jacquez JA, Koopman JS, Simon CP, Longini IM, Jr. **Role of the primary infection in epidemics of HIV infection in gay cohorts**. *J Acquir Immune Defic Syndr* 1994,7:1169-1184 [PMID#: 7932084].

128. Koopman JS, Jacquez JA, Welch GW, Simon CP, Foxman B, Pollock SM*, et al.* **The role of early HIV infection in the spread of HIV through populations**. *J Acquir Immune Defic Syndr Hum Retrovirol* 1997,14:249-258 [PMID#: 9117458].

129. Xiridou M, Geskus R, de Wit J, Coutinho R, Kretzschmar M. **Primary HIV infection as source of HIV transmission within steady and casual partnerships among homosexual men**. *AIDS* 2004,18:1311-1320 [PMID#: 15362664].

130. Hollingsworth D, Anderson R, Fraser C. **Has the Role of Primary Infection in the Epidemiology of HIV been Overstated?** . *13th Conference on Retroviruses and Opportunistic Infections*. Denver, CO, February 5-8, 2006 [#913].

131. Schmitz T, Kleinkauf N, Klempa B, Ringe H, Varnholt V, Grosch-Worner I. **Transmission of human immunodeficiency virus type 1 nevirapine resistance mutation K103N from a treatment-naive mother to her child**. *Pediatr Infect Dis J* 2006,25:275-276 [PMID#: 16511398].

132. Romano L, Venturi G, Vivarelli A, Galli L, Zazzi M. **Detection of a drug-resistant human immunodeficiency virus variant in a newly infected heterosexual couple**. *Clin Infect Dis* 2002,34:116-117 [PMID#: 11731954].

133. Brenner BG, Roger M, Routy JP, Moisi D, Ntemgwa M, Matte C*, et al.* **High Rates of Forward Transmission Events after Acute/Early HIV-1 Infection**. *J Infect Dis* 2007,195:951-959 [PMID#: 17330784].

134. Lindstrom A, Ohlis A, Huigen M, Nijhuis M, Berglund T, Bratt G*, et al.* **HIV-1 transmission cluster with M41L 'singleton' mutation and decreased transmission of resistance in newly diagnosed Swedish homosexual men**. *Antivir Ther* 2006,11:1031-1039 [PMID#: 17302373].

135. Drumright L, Little S, Richman D, Frost S. **Age discordance and drug resistance predict clustering of HIV among recently infected MSM in San Diego, Californa**. *14th Conference on Retroviruses and Opportunistic Infections*. Los Angeles, California; February 25-28, 2007 [abstract #654].

136. Graham SM, Holte SE, Peshu NM, Richardson BA, Panteleeff DD, Jaoko WG*, et al.* **Initiation of antiretroviral therapy leads to a rapid decline in cervical and vaginal HIV-1 shedding**. *AIDS* 2007,21:501-507 [PMID#: 17301569].

137. Blower SM, Aschenbach AN, Gershengorn HB, Kahn JO. **Predicting the unpredictable: transmission of drug-resistant HIV**. *Nat Med* 2001,7:1016-1020 [PMID#: 11533704].

138. Blower SM, Aschenbach AN, Kahn JO. **Predicting the transmission of drug-resistant HIV: comparing theory with data**. *Lancet Infect Dis* 2003,3:10-11 [PMID#: 12505024].

139. Stekler J, Swenson PD, Wood RW, Handsfield HH, Golden MR. **Targeted screening for primary HIV infection through pooled HIV-RNA testing in men who have sex with men**. *AIDS* 2005,19:1323-1325 [PMID#: 16052089].

140. Pilcher CD, Fiscus SA, Nguyen TQ, Foust E, Wolf L, Williams D*, et al.* **Detection of acute infections during HIV testing in North Carolina**. *N Engl J Med* 2005,352:1873-1883 [PMID#: 15872202].

141. Patel P, Klausner JD, Bacon OM, Liska S, Taylor M, Gonzalez A*, et al.* **Detection of acute HIV infections in high-risk patients in California**. *J Acquir Immune Defic Syndr* 2006,42:75-79 [PMID#: 16763493].

142. Priddy FH, Pilcher CD, Moore RH, Tambe P, Park MN, Fiscus SA*, et al.* **Detection of Acute HIV Infections in an Urban HIV Counseling and Testing Population in the United States**. *J Acquir Immune Defic Syndr* 2006: [PMID#: 17091022].

143. Halvas EK, Aldrovandi GM, Balfe P, Beck IA, Boltz VF, Coffin JM*, et al.* **Blinded, multicenter comparison of methods to detect a drug-resistant mutant of human immunodeficiency virus type 1 at low frequency**. *J Clin Microbiol* 2006,44:2612-2614 [PMID#: 16825395].

144. Shi C, Eshleman SH, Jones D, Fukushima N, Hua L, Parker AR*, et al.* **LigAmp for sensitive detection of single-nucleotide differences**. *Nat Methods* 2004,1:141-147 [PMID#: 15782177].

145. Edelstein RE, Nickerson DA, Tobe VO, Manns-Arcuino LA, Frenkel LM. **Oligonucleotide ligation assay for detecting mutations in the human immunodeficiency virus type 1 pol gene that are associated with resistance to zidovudine, didanosine, and lamivudine**. *J Clin Microbiol* 1998,36:569-572 [PMID#: 9466779].

146. Beck IA, Mahalanabis M, Pepper G, Wright A, Hamilton S, Langston E, Frenkel LM. **Rapid and sensitive oligonucleotide ligation assay for detection of mutations in human immunodeficiency virus type 1 associated with high-level resistance to protease inhibitors**. *J Clin Microbiol* 2002,40:1413-1419 [PMID#: 11923366].

147. Ellis GM, Mahalanabis M, Beck IA, Pepper G, Wright A, Hamilton S*, et al.* **Comparison of oligonucleotide ligation assay and consensus sequencing for detection of drug-resistant mutants of human immunodeficiency virus type 1 in peripheral blood mononuclear cells and plasma**. *J Clin Microbiol* 2004,42:3670-3674 [PMID#: 15297515].

148. Paredes R, Marconi VC, Campbell TB, Kuritzkes DR. **Systematic evaluation of allele-specific real-time PCR for the detection of minor HIV-1 variants with pol and env resistance mutations**. *J Virol Methods* 2007,146:136-146 [PMID#: 17662474].

149. Cai F, Chen H, Hicks CB, Bartlett JA, Zhu J, Gao F. **Detection of minor drug-resistant populations by parallel allele-specific sequencing**. *Nat Methods* 2007,4:123-125 [PMID#: 17206150].

150. Landegren U, Nilsson M, Kwok PY. **Reading bits of genetic information: methods for single-nucleotide polymorphism analysis**. *Genome Res* 1998,8:769-776 [PMID#: 9724323].

151. **Stanford University HIV Drug Resistance Database, http://hivdb.stanford.edu/pages/algs/HIVdb.html, accessed July 17, 2007**. In. accessed July 17, 2007 ed: .

152. Wallis CL, Mahomed I, Morris L, Chidarikire T, Stevens G, Rekhviashvili N, Stevens W. **Evaluation of an oligonucleotide ligation assay for detection of mutations in HIV-1 subtype C individuals who have high level resistance to nucleoside reverse transcriptase inhibitors and non-nucleoside reverse transcriptase inhibitors**. *J Virol Methods* 2005,125:99-109 [PMID#: 15794978].

153. Vega Y, Perez-Alvarez L, Delgado E, Munoz M, Casado G, Carmona R*, et al.* **Oligonucleotide ligation assay for detection of mutations associated with reverse transcriptase and protease inhibitor resistance in non-B subtypes and recombinant forms of human immunodeficiency virus type 1**. *J Clin Microbiol* 2005,43:5301-5304 [PMID#: 16208003].

154. Beck IA, Crowell C, Kittoe R, Bredell H, Machaba M, Willamson C*, et al.* **Optimization of the Oligonucleotide Ligation Assay, a Rapid and Inexpensive Test for Detection of HIV-1 Drug Resistance Mutations, for Non-North American Variants**. *J Acquir Immune Defic Syndr* 2008,48:418-427 [PMID#: 18614915].

155. Jallow S, Kaye S, Schutten M, Brandin E, Albert J, McConkey SJ*, et al.* **Development and evaluation of an oligonucleotide ligation assay for detection of drug resistance-associated mutations in the human immunodeficiency virus type 2 pol gene**. *J Clin Microbiol* 2007,45:1565-1571 [PMID#: 17329450].

156. Lalonde MS, Troyer RM, Syed AR, Bulime S, Demers K, Bajunirwe F, Arts EJ. **Sensitive oligonucleotide ligation assay for low-level detection of nevirapine resistance mutations in human immunodeficiency virus type 1 quasispecies**. *J Clin Microbiol* 2007,45:2604-2615 [PMID#: 17567789].

157. Micek M, Blanco A, Matediane E, Matunha L, Beck I, Dross S*, et al.* **Nevirapine-resistant HIV-1 among Mozambican Infants Infected in Utero vs Intra-partum or Early Postpartum**. *In 14th Conference on Retroviruses and Opportunistic Infections; Los Angeles, CA; February 25-28, 2007 [abstract #92]*.

158. Troyer RM, Lalonde MS, Fraundorf E, Demers KR, Kyeyune F, Mugyenyi P*, et al.* **A radiolabeled oligonucleotide ligation assay demonstrates the high frequency of nevirapine resistance mutations in HIV type 1 quasispecies of NVP-treated and untreated mother-infant pairs from Uganda**. *AIDS Res Hum Retroviruses* 2008,24:235-250 [PMID#: 18284323].

159. Liu SL, Schacker T, Musey L, Shriner D, McElrath MJ, Corey L, Mullins JI. **Divergent patterns of progression to AIDS after infection from the same source: human immunodeficiency virus type 1 evolution and antiviral responses**. *J Virol* 1997,71:4284-4295 [PMID#: 9151816].

160. Chun TW, Davey RT, Jr., Ostrowski M, Shawn Justement J, Engel D, Mullins JI, Fauci AS. **Relationship between pre-existing viral reservoirs and the re-emergence of plasma viremia after discontinuation of highly active anti-retroviral therapy**. *Nat Med* 2000,6:757-761 [PMID#: 10888923].

161. Chun TW, Engel D, Mizell SB, Hallahan CW, Fischette M, Park S*, et al.* **Effect of interleukin-2 on the pool of latently infected, resting CD4+ T cells in HIV-1-infected patients receiving highly active anti-retroviral therapy**. *Nat Med* 1999,5:651-655 [PMID#: 10371503].

162. Malhotra U, Berrey MM, Huang Y, Markee J, Brown DJ, Ap S*, et al.* **Effect of combination antiretroviral therapy on T-cell immunity in acute human immunodeficiency virus type 1 infection**. *J Infect Dis* 2000,181:121-131 [PMID#: 10608758].

163. Musey L, Hughes J, Schacker T, Shea T, Corey L, McElrath MJ. **Cytotoxic-T-cell responses, viral load, and disease progression in early human immunodeficiency virus type 1 infection**. *N Engl J Med* 1997,337:1267-1274 [PMID#: 9345075].

164. Schacker T, Collier AC, Hughes J, Shea T, Corey L. **Clinical and epidemiologic features of primary HIV infection**. *Ann Intern Med* 1996,125:257-264 [PMID#: 8678387].

165. Zhu T, Corey L, Hwangbo Y, Lee JM, Learn GH, Mullins JI, McElrath MJ. **Persistence of extraordinarily low levels of genetically homogeneous human immunodeficiency virus type 1 in exposed seronegative individuals**. *J Virol* 2003,77:6108-6116 [PMID#: 12743268].

166. Zhu T, Muthui D, Holte S, Nickle D, Feng F, Brodie S*, et al.* **Evidence for human immunodeficiency virus type 1 replication in vivo in CD14(+) monocytes and its potential role as a source of virus in patients on highly active antiretroviral therapy**. *J Virol* 2002,76:707-716 [PMID#: 11752161].

167. Holte SE, Melvin AJ, Mullins JI, Tobin NH, Frenkel LM. **Density-dependent decay in HIV-1 dynamics**. *J Acquir Immune Defic Syndr* 2006,41:266-276 [PMID#: 16540927].

168. Troyer RM, Collins KR, Abraha A, Fraundorf E, Moore DM, Krizan RW*, et al.* **Changes in human immunodeficiency virus type 1 fitness and genetic diversity during disease progression**. *J Virol* 2005,79:9006-9018 [PMID#: 15994794].

169. Horton H, Frank I, Baydo R, Jalbert E, Penn J, Wilson S*, et al.* **Preservation of T cell proliferation restricted by protective HLA alleles is critical for immune control of HIV-1 infection**. *J Immunol* 2006,177:7406-7415 [PMID#: 17082660].

170. Stekler J, Collier A. **Treatment of Primary HIV**. *Curr Infect Dis Rep* 2002,4:81-87 [PMID#: 11853661].

171. Stekler J, Collier AC. **Primary HIV Infection**. *Curr HIV/AIDS Rep* 2004,1:68-73 [PMID#: 16091225].

172. Stekler J, Maenza J, Stevens C, Holte S, Malhotra U, McElrath MJ*, et al.* **Abacavir hypersensitivity reaction in primary HIV infection**. *AIDS* 2006,20:1269-1274 [PMID#: 16816555].

173. Stekler J, Maenza J, Stevens CE, Swenson PD, Coombs RW, Wood RW*, et al.* **Screening for acute HIV infection: lessons learned**. *Clin Infect Dis* 2007,44:459-461 [PMID#: 17205460].

174. Liu Y, McNevin J, Cao J, Zhao H, Genowati I, Wong K*, et al.* **Selection on the human immunodeficiency virus type 1 proteome following primary infection**. *J Virol* 2006,80:9519-9529 [PMID#: 16973556].

175. Liu Y, Mullins JI, Mittler JE. **Waiting times for the appearance of cytotoxic T-lymphocyte escape mutants in chronic HIV-1 infection**. *Virology* 2006,347:140-146 [PMID#: 16387340].

176. Malhotra U, Nolin J, Mullins JI, McElrath MJ. **Comprehensive epitope analysis of cross-clade Gag-specific T-cell responses in individuals with early HIV-1 infection in the US epidemic**. *Vaccine* 2007,25:381-390 [PMID#: 17112643].

177. Malhotra U, Li F, Nolin J, Allison M, Zhao H, Mullins JI*, et al.* **Enhanced detection of human immunodeficiency virus type 1 (HIV-1) Nef-specific T cells recognizing multiple variants in early HIV-1 infection**. *J Virol* 2007,81:5225-5237 [PMID#: 17329342].

178. Chun TW, Justement JS, Moir S, Hallahan CW, Maenza J, Mullins JI*, et al.* **Decay of the HIV reservoir in patients receiving antiretroviral therapy for extended periods: implications for eradication of virus**. *J Infect Dis* 2007,195:1762-1764 [PMID#: 17492591].

179. Cao J, McNevin J, McSweyn M, Liu Y, Mullins JI, McElrath MJ. **Novel cytotoxic T-lymphocyte escape mutation by a three-amino-acid insertion in the human immunodeficiency virus type 1 p6Pol and p6Gag late domain associated with drug resistance**. *J Virol* 2008,82:495-502 [PMID#: 17942528].

180. Mei Y, Wang L, Holte SE. **A comparison of methods for determining HIV viral set point**. *Stat Med* 2008,27:121-139 [PMID#: 17787030].

181. **Public Health - Seattle & King County, http://www.metrokc.gov/health/apu/epi/epistats.htm, accessed August 15, 2008**. In: .

182. Stekler J, Swenson PD, Coombs RW, Dragavon J, Wood RW, Golden MR. **Anonymous testing and rapid testing in screening for acute HIV infection**. *14th Conference on Retroviruses and Opportunistic Infections, Los Angeles, CA.*, February 25-28, 2007 [abstract #340].

183. Stekler J, Wood RW, Swenson PD, Golden M. **Negative rapid HIV antibody testing during early HIV infection**. *Ann Intern Med* 2007,147:147-148 [PMID#: 17638724].

184. Stekler J, Holte S, Maenza J, Stevens CE, Collier AC. **Clinical outcomes of antiretroviral treatment initiated during primary HIV infection**. *14th Conference on Retroviruses and Opportunistic Infections; Los Angeles, CA; February 25-28, 2007 [abstract #346]*.

185. Gulick RM, Ribaudo HJ, Shikuma CM, Lustgarten S, Squires KE, Meyer WA, 3rd*, et al.* **Triple-nucleoside regimens versus efavirenz-containing regimens for the initial treatment of HIV-1 infection**. *N Engl J Med* 2004,350:1850-1861 [PMID#: 15115831].

186. Truong HM, Berrey MM, Shea T, Diem K, Corey L. **Concordance between HIV source partner identification and molecular confirmation in acute retroviral syndrome**. *J Acquir Immune Defic Syndr* 2002,29:232-243 [PMID#: 11873072].

187. Liu Y, Curlin ME, Diem K, Zhao H, Ghosh AK, Zhu H*, et al.* **Env length and N-linked glycosylation following transmission of human immunodeficiency virus Type 1 subtype B viruses**. *Virology* 2008,374:229-233 [PMID#: 18314154].

188. Booth CL, Garcia-Diaz AM, Youle MS, Johnson MA, Phillips A, Geretti AM. **Prevalence and predictors of antiretroviral drug resistance in newly diagnosed HIV-1 infection**. *J Antimicrob Chemother* 2007,59:517-524 [PMID#: 17213262].

189. Colfax GN, Vittinghoff E, Grant R, Lum P, Spotts G, Hecht FM. **Frequent methamphetamine use is associated with primary non-nucleoside reverse transcriptase inhibitor resistance**. *AIDS* 2007,21:239-241 [PMID#: 17197817].

190. Kassutto S, Maghsoudi K, Johnston MN, Robbins GK, Burgett NC, Sax PE*, et al.* **Longitudinal analysis of clinical markers following antiretroviral therapy initiated during acute or early HIV type 1 infection**. *Clin Infect Dis* 2006,42:1024-1031 [PMID#: 16511771].

191. Paredes R, Mocroft A, Kirk O, Lazzarin A, Barton SE, van Lunzen J*, et al.* **Predictors of virological success and ensuing failure in HIV-positive patients starting highly active antiretroviral therapy in Europe: results from the EuroSIDA study**. *Arch Intern Med* 2000,160:1123-1132 [PMID#: 10789605].

192. Demeter LM, Hughes MD, Coombs RW, Jackson JB, Grimes JM, Bosch RJ*, et al.* **Predictors of virologic and clinical outcomes in HIV-1-infected patients receiving concurrent treatment with indinavir, zidovudine, and lamivudine. AIDS Clinical Trials Group Protocol 320**. *Ann Intern Med* 2001,135:954-964 [PMID#: 11730396].

193. Smith CJ, Staszewski S, Sabin CA, Nelson M, Dauer B, Gute P*, et al.* **Use of viral load measured after 4 weeks of highly active antiretroviral therapy to predict virologic outcome at 24 weeks for HIV-1-positive individuals**. *J Acquir Immune Defic Syndr* 2004,37:1155-1159 [PMID#: 15319675].

194. Reichelderfer PS, Coombs RW, Wright DJ, Cohn J, Burns DN, Cu-Uvin S*, et al.* **Effect of menstrual cycle on HIV-1 levels in the peripheral blood and genital tract. WHS 001 Study Team**. *AIDS* 2000,14:2101-2107 [PMID#: 11061650].

195. Li CC, Seidel KD, Coombs RW, Frenkel LM. **Detection and quantification of human immunodeficiency virus type 1 p24 antigen in dried whole blood and plasma on filter paper stored under various conditions**. *J Clin Microbiol* 2005,43:3901-3905 [PMID#: 16081929].

196. Coombs RW, Speck CE, Hughes JP, Lee W, Sampoleo R, Ross SO*, et al.* **Association between culturable human immunodeficiency virus type 1 (HIV-1) in semen and HIV-1 RNA levels in semen and blood: evidence for compartmentalization of HIV-1 between semen and blood**. *J Infect Dis* 1998,177:320-330 [PMID#: 9466517].

197. Fiscus SA, Brambilla D, Coombs RW, Yen-Lieberman B, Bremer J, Kovacs A*, et al.* **Multicenter evaluation of methods to quantitate human immunodeficiency virus type 1 RNA in seminal plasma**. *J Clin Microbiol* 2000,38:2348-2353 [PMID#: 10835001].

198. Delwart EL, Shpaer EG, Louwagie J, McCutchan FE, Grez M, Rubsamen-Waigmann H, Mullins JI. **Genetic relationships determined by a DNA heteroduplex mobility assay: analysis of HIV-1 env genes**. *Science* 1993,262:1257-1261 [PMID#: 8235655].

199. Beck IA, Drennan KD, Melvin AJ, Mohan KM, Herz AM, Alarcon J*, et al.* **Simple, sensitive, and specific detection of human immunodeficiency virus type 1 subtype B DNA in dried blood samples for diagnosis in infants in the field**. *J Clin Microbiol* 2001,39:29-33 [PMID#: 11136743].

200. Gottlieb GS, Nickle DC, Jensen MA, Wong KG, Grobler J, Li F*, et al.* **Dual HIV-1 infection associated with rapid disease progression**. *Lancet* 2004,363:619-622 [PMID#: 14987889].

201. Lalonde MS, Troyer RM, Syed AR, Bulime S, Demers K, Bajunirwe F, Arts EJ. **A sensitive oligonucleotide ligation assay for low-level detection of nevirapine resistance mutations in HIV-1 quasispecies**. *J Clin Microbiol* 2007 in press: [PMID#:

202. Johnson JA, Rompay KK, Delwart E, Heneine W. **A rapid and sensitive real-time PCR assay for the K65R drug resistance mutation in SIV reverse transcriptase**. *AIDS Res Hum Retroviruses* 2006,22:912-916 [PMID#: 16989618].

203. Johnson JA, Li JF, Morris L, Martinson N, Gray G, McIntyre J, Heneine W. **Emergence of drug-resistant HIV-1 after intrapartum administration of single-dose nevirapine is substantially underestimated**. *J Infect Dis* 2005,192:16-23 [PMID#: 15942889].

204. Johnson JA, Li JF, Wei X, Lipscomb J, Bennett D, Brant A*, et al.* **Simple PCR assays improve the sensitivity of HIV-1 subtype B drug resistance testing and allow linking of resistance mutations**. *PLoS ONE* 2007,2:e638 [PMID#: 17653265].

205. Chohan B, Lavreys L, Rainwater SM, Overbaugh J. **Evidence for frequent reinfection with human immunodeficiency virus type 1 of a different subtype**. *J Virol* 2005,79:10701-10708 [PMID#: 16051862].

206. Smith DM, Wong JK, Hightower GK, Ignacio CC, Koelsch KK, Daar ES*, et al.* **Incidence of HIV superinfection following primary infection**. *JAMA* 2004,292:1177-1178 [PMID#: 15353529].

207. Hu DJ, Subbarao S, Vanichseni S, Mock PA, Ramos A, Nguyen L*, et al.* **Frequency of HIV-1 dual subtype infections, including intersubtype superinfections, among injection drug users in Bangkok, Thailand**. *AIDS* 2005,19:303-308 [PMID#: 15718841].

208. Branson BM, Handsfield HH, Lampe MA, Janssen RS, Taylor AW, Lyss SB, Clark JE. **Revised recommendations for HIV testing of adults, adolescents, and pregnant women in health-care settings**. *MMWR Recomm Rep* 2006,55:1-17; quiz CE11-14 [PMID#: 16988643].

209. Holtgrave DR, Pinkerton SD. **Can increasing awareness of HIV seropositivity reduce infections by 50% in the United States?** *J Acquir Immune Defic Syndr* 2007,44:360-363 [PMID#: 17159653].

210. Yerly S, Vora S, Rizzardi P, Chave JP, Vernazza PL, Flepp M*, et al.* **Acute HIV infection: impact on the spread of HIV and transmission of drug resistance**. *AIDS* 2001,15:2287-2292 [PMID#: 11698702].

211. Pao D, Fisher M, Hue S, Dean G, Murphy G, Cane PA*, et al.* **Transmission of HIV-1 during primary infection: relationship to sexual risk and sexually transmitted infections**. *AIDS* 2005,19:85-90 [PMID#: 15627037].
